# Concurrent Subcellular Delivery of Hydrogen Sulfide and a Payload with Near-Infrared Light

**DOI:** 10.1021/jacsau.4c00445

**Published:** 2024-07-05

**Authors:** Katarzyna Hanc, Hana Janeková, Peter Štacko

**Affiliations:** Department of Chemistry, University of Zurich, Winterthurerstrasse 190, CH-8057 Zurich, Switzerland

**Keywords:** cyanine, hydrogen sulfide, photocage, near-infrared, uncaging, mitochondria, concurrent

## Abstract

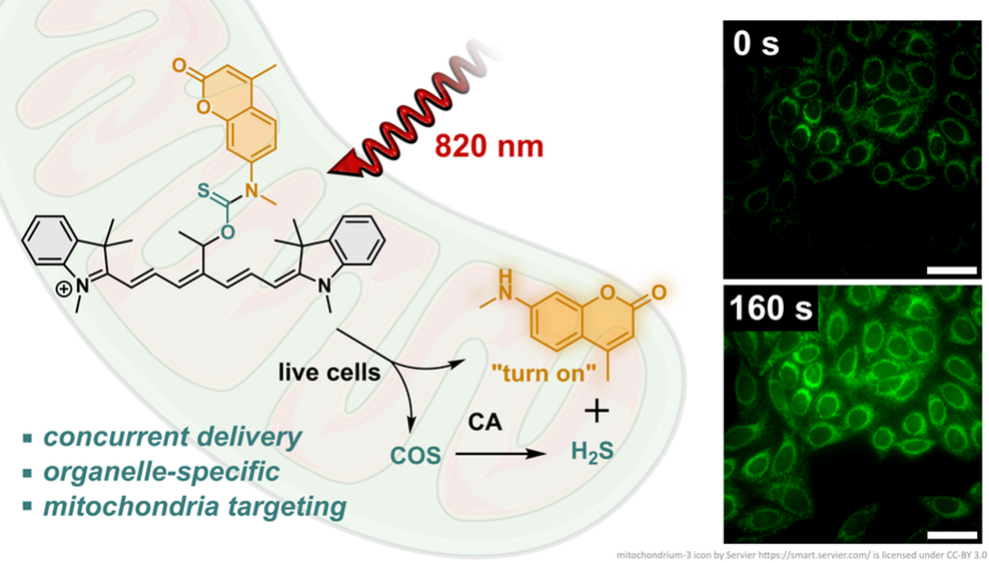

Hydrogen sulfide (H_2_S) is a gaseous signaling molecule, exerting crucial regulatory functions in organelles and cellular environments. H_2_S exhibits high therapeutic potential and synergistic effects with other drugs, and its potency is notably enhanced through organelle-specific targeting. Yet, the navigation of light-activated H_2_S donors to specific organelles remains absent. Here, we report the first organelle-specific photocage that simultaneously delivers H_2_S and a payload with subcellular precision to mitochondria of live human cells using tissue-penetrating near-infrared light as a trigger. The fluorogenic payload enables real-time monitoring of the process, and we demonstrate the concurrent uncaging in mitochondria through a combination of fluorescence microscopy and mitochondria-specific fluorescent probes. We anticipate that these photocages will permit the precise delivery of H_2_S-drug combinations with exceptional spatiotemporal control, thereby driving the harnessing of known synergistic effects and the discovery of novel therapeutic strategies.

## Introduction

Hydrogen sulfide (H_2_S), produced enzymatically from cysteine, is an endogenous signaling molecule which plays a crucial role in regulating organelle function and stress.^1−3^ Its elevated levels exhibit anti-inflammatory, vasorelaxant,^3^ and cytoprotective effects in cardiovascular and nervous systems,^4,5^ and its anticancer effects in melanoma,^6^ gastric^7^ and colon^8,9^ cancer, or osteocarcinoma^10^ are also well documented. Major drawbacks associated with the delivery of H_2_S by inorganic salts led to the emergence of H_2_S donors^2,3^ activated by reactive oxygen species,^11^ cysteine,^12^ pH changes,^13,14^ or enzymes.^15,16^ Strong synergistic effects between H_2_S and nonsteroidal anti-inflammatory drugs (NSAID) motivate the development of H_2_S-releasing NSAID derivatives with many of them undergoing clinical trials.^17,18^

**Figure 1:**
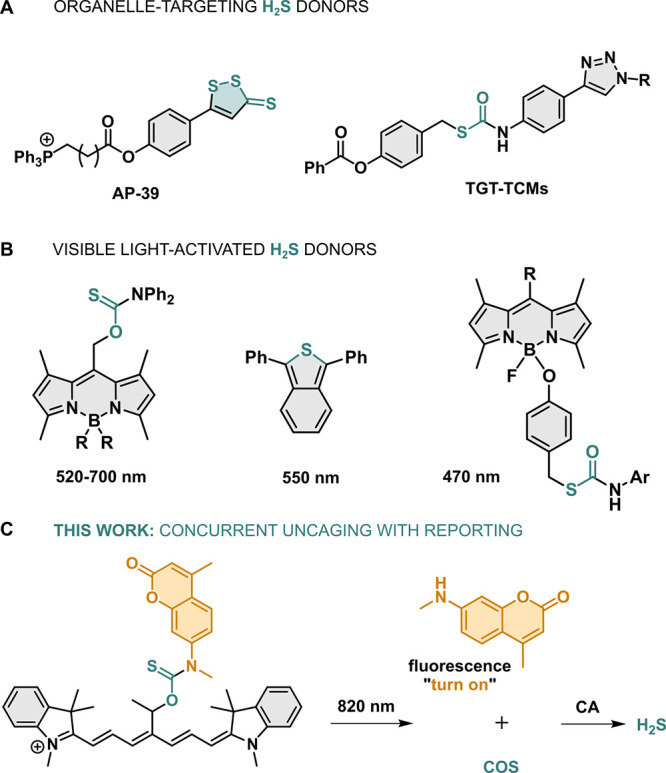
(A) Selected organelle-targeting and (B) light-activated H_2_S donors. (C) Targeted concurrent delivery of H_2_S and an amine payload from Cy7 photocages with near-infrared light.

Evidence suggests that the enzymes involved in the H_2_S production can be actively localized in various organelles—e.g., cystathionine-β-synthase accumulates in the mitochondria in liver ischemia.^19^ Since dysfunction of organelles can result in diseases, such selective directing of therapeutic agents to specific organelles might be part of the defense mechanism in our bodies to mitigate them.^20^ Malfunctional mitochondria result in oxidative stress linked to diseases such as Parkinson’s disease,^21^ cardiovascular diseases,^22^ or type 2 diabetes.^23^ Defects in endoplasmic reticulum (ER) or the Golgi apparatus have been connected to Alzheimer’s disease, amyotrophic lateral sclerosis, and Creutzfeldt-Jakob disease.^24,25^ Consequently, donors that target H_2_S delivery to organelles appeared to mimic the localization of its production (Figure 1). AP39, targeting mitochondria, has been successful in alleviating ischemia injury,^26^ protecting against myocardial injury,^27^ or exhibiting neuroprotective activity in brain ischemia model.^28^ Mitochondria-targeting HSD-B provided cytoprotection in hypoxia injury model in H9c2 cardiomyocytes,^29^ whereas UTS-2 was shown to localize in lysosomes and release H_2_S in the presence of thiols.^30^ Recent work of Pluth and Gilbert corroborates these reports by demonstrating that organelle-specific thiocarbamate (TCM) donors significantly enhance the therapeutic effects of H_2_S.^20^

Light-activated organic H_2_S donors have recently emerged to advance the spatiotemporal resolution of H_2_S delivery. Yet the majority of these systems, based on *o*-nitrobenzyl,^31^ ketoprofanate,^32^ or xanthone,^33^ relies on biologically adverse UV light. Only two examples based on BODIPY scaffold extended the operating wavelength to the visible^34^ and edge of the near-infrared (700 nm) region,^35^ and none of the photocage-based donors have been targeted to specific organelles. Surprisingly, the organic fragment (amine/alcohol) that accompanies carbonyl sulfide (COS) release from the thiocarbamate/thiocarbonate linker employed in TCM donors continues to be seriously neglected. Despite evidence of synergistic effects between H_2_S and drugs,^36−38^ efforts to deliver such species concurrently remains largely absent. Recognizing the importance of these needs, we report H_2_S donors that are activated with tissue-penetrating, near-infrared light (>800 nm), and concurrently deliver amine payload within the same cellular compartment.

We re-engineered Cy7-based photocages that release amines, alcohols or phenols upon activation with NIR light.^39−41^ We reasoned that *S*-/*O*-thiocarbamates, leaving groups of comparable quality, should also be cleaved by light and subsequently fragmented into COS and the amine. The ubiquitous distribution of different carbonic anhydrase (CA) isoforms throughout the cell then enables the conversion of COS into H_2_S,^42,43^ and its concurrent delivery with the amine payload. We anticipated that the COS fragmentation would also promote the direct uncaging pathway,^40^ rendering these dual-action photocages not reliant on oxygen. The rationale for employing 7-aminocoumarin **2** as a model payload was twofold: (a) it serves as a model payload that can be substituted for a drug of choice, and (b) it acts as a direct fluorogenic reporter of the H_2_S-uncaging, inspired by other systems.^44−47^ Connection via the thiocarbamate linker disturbs the push–pull electronic system, producing a blueshift of the absorption maxima, and at the same time, its fluorescence is quenched by the appending cyanine. Both properties are restored upon uncaging, resulting in a strong turn-on fluorescence response.

In our design, we took advantage of well-established propensity of cyanines to localize in mitochondria due to their cationic nature.^48,49^ However, different targeting moieties which have been realized in cyanines, such as for ER,^50^ Golgi apparatus,^51^ or lysosomes,^52,53^ hold a promise to extend this strategy to other organelles as well.

## Results and Discussion

**Scheme 1:**
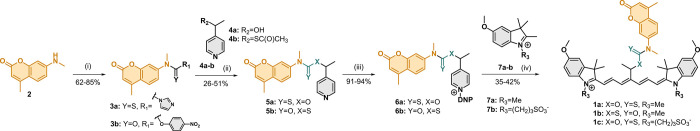
Synthesis of the Dual-Action Photocages i) **3a**: TCDI, THF, 60 °C; **3b**: 4-nitrophenyl chloroformate, DIPEA, MeCN, reflux; (ii) **5a**: NaH, DMF, −5 °C to rt; **5b**: NaOMe, MeOH, 0 °C to rt; (iii) MeCN, 35 °C; (iv) AcOK, MeCN, rt. Caged **2** and COS are depicted in orange and blue colors, respectively. Counter anions are omitted for clarity.

The synthesis of photocages **1a**–**c** started by the activation of the payload **2**(54) with thiocarbonyldiimidazole (TCDI) or 4-nitrophenyl chloroformate to provide activated (thio)carbamates **3a**–**b** in excellent yields (Scheme 1). Reaction of **3a** with the alcohol **4a** in the presence of NaH led to *O*-thiocarbamate-decorated pyridine **5a**, which was subsequently converted into Zincke salt **6a** by a reaction with dinitrophenyl triflate (DNP-OTf) in excellent yield. One-pot ring-opening of the pyridinium ring^55^ and condensation with the heterocycles **7a** or **7b** provided the target photocages **1a** and **1c**, respectively, in moderate yields. The synthesis of the *S*-thiocarbamate isomer required the preparation of 1-(pyridin-4-yl)ethane-1-thiol **4b**. Although its preparation has been described,^56^ we found it highly susceptible to oxidation during its isolation. We therefore opted for its in situ alcoholysis, followed by a substitution of carbamate **3b**, affording *S*-thiocarbamate **5b** in a moderate yield. Its analogous reaction with DNP-OTf produced Zincke salt **6b** which was smoothly converted into the target photocage **1b** bearing the *S*-thiocarbamate payload. *N*-Methyl substituent on payload **2** was installed because analogous carbamates derived from primary amines are not compatible with the final step of the cyanine synthesis, presumably due to hydrogen abstraction by the acetate. Contrary to our recent work,^40^^1^H NMR spectroscopy of thiocarbamate photocages **1a**–**c** showed only single species without any indication of rotamers.

We next investigated photocages **1a**–**b** by UV–vis absorption and emission spectroscopies (Table 1). Both derivatives in *N*-(2-hydroxyethyl)piperazine-*N*′-ethanesulfonic acid (HEPES) [pH 7.4, 20 mM, 10% dimethylformamide (DMF)] display a strong absorption band located in the center of the NIR region at λ_max_ = 809–818 nm typical for cyanines. The photocages exhibit weak emission (Φ_F_ < 0.02) with Stokes-shifts of ∼500 cm^–1^, comparable to previously reported red-shifted cyanines.^57^

**Table 1:** Photophysical Properties of Photocages **1a–b**

	λ_abs_/nm^a^	λ_em_/nm^a^	ε^a^^,^^b^	yield of 2 (aer)/%^c^	yield of 2 (deg)/%^d^	yield of H_2_S/%^e^
**1a**	812	838	80 500	43 ± 1 (52^f^)	47	47 ± 2
**1b**	805	832	92 200	11 ± 2 (12^f^)	<2	15 ± 3

a Determined in HEPES (pH 7.4, 20 mM) with 10% DMF.

b The molar absorption coefficient, ε_max_/mol^–1^ dm^3^ cm^–1^.

c Chemical yield of the payload **2** uncaged with 810 nm light in HEPES with 10% DMF determined by emission spectroscopy.

d Chemical yield of the payload **2** uncaged with 810 nm light in CD_3_OD under oxygen-free conditions.

e Chemical yield of H_2_S uncaged with 810 nm light in HEPES with 1% MeOH or 5% MeCN determined by methylene blue assay.

f Determined by ^1^H NMR spectroscopy in DMSO-*d*_6_.

Interestingly, **1b** shows much lower solubility in aqueous media and consequently a prominent blue-shifted band attributed to its H-aggregates, manifested also by the partitioning coefficients (log *P* values of 0.9 and 1.65 for **1a** and **1b**, respectively). For this reason, we employed DMF as a cosolvent in the studies. On the other hand, **1c** containing appending sulfonated chains was fully soluble even without additional cosolvents.

**Figure 2:**
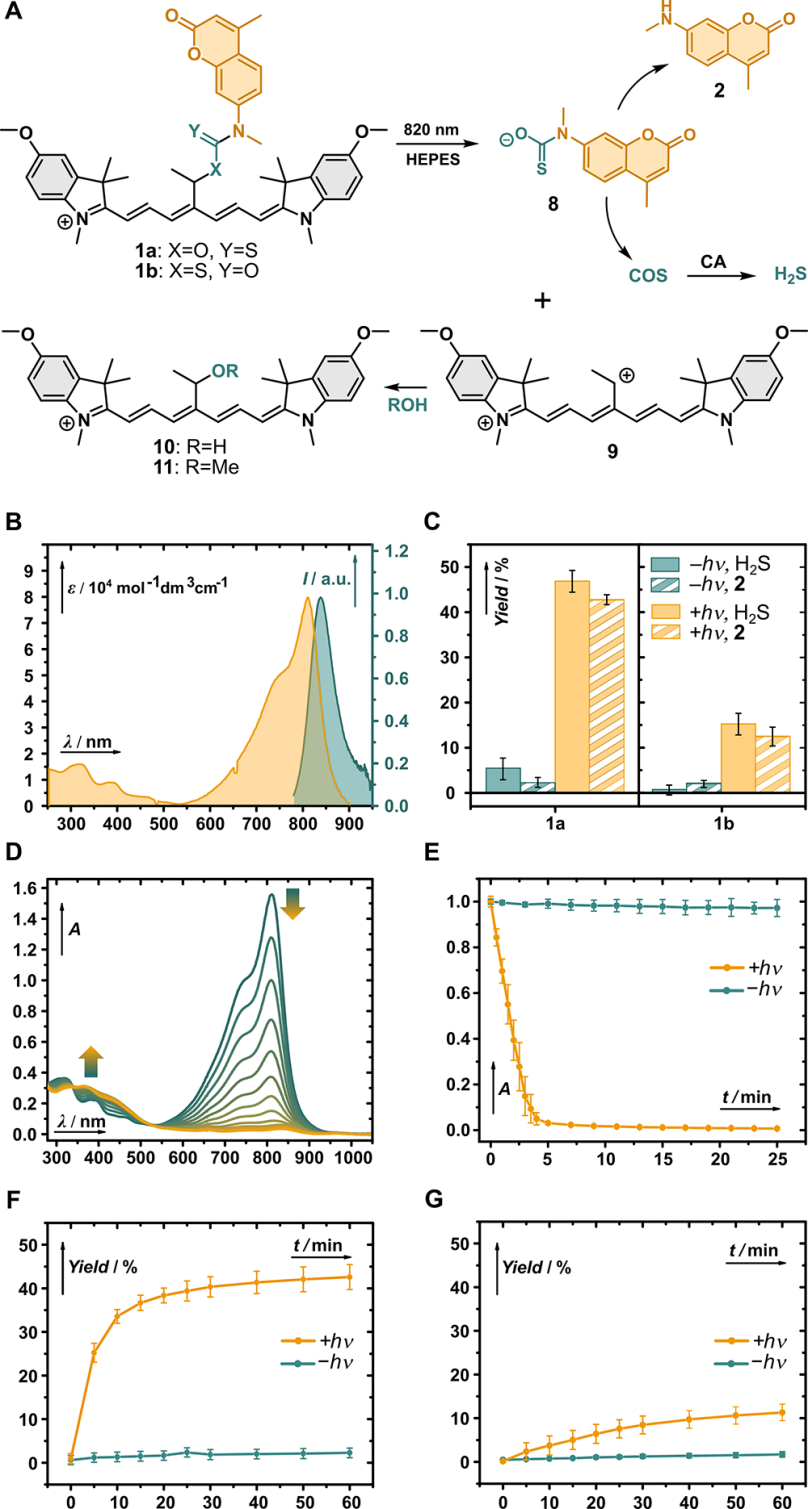
(A) Schematic representation of uncaging of **2** and H_2_S from **1a** through the thiocarbamate **8** intermediate and formation of **9**. (B) Absorption (orange) and emission (blue) spectra of **1a** in HEPES (with 10% DMF) and methanol, respectively. (C) Chemical yields of **2** (striped) and H_2_S (solid) released from **1a**–**b** upon irradiation (orange) and in the dark (blue). (D) Irradiation of **1a** in HEPES (with 10% DMF) at 820 nm followed by UV–vis spectroscopy at 60 s intervals (blue to orange). (E) Kinetic trace at absorption maxima of **1a** upon irradiation with 820 nm (orange) and in the dark (blue). Time profile of **2** uncaging from **1a** (F) and **1b** (G) under 820 nm irradiation (orange) and in the dark (blue).

To our great surprise, the two isomers also exhibited highly contrasting photochemical behavior. The irradiation of **1a** in HEPES (pH 7.4, 20 mM, 10% DMF) at 820 nm (∼40 mW cm^–2^) led to the disappearance of the cyanine band, accompanied by a formation of a band at ∼365 nm attributed to the liberated payload **2** (Figure 2D). On the contrary, irradiation of **1b** under identical conditions showed only a minor increase in this range; instead, a different band at ∼438 nm was formed (Figure S38). The rates of the processes were contrasting—a 20 μM solution of **1a** fully photolyzed in <5 min (Figure 2E), whereas the conversion of **1b** required >30 min of irradiation (Figures S37 and S38). No changes in the spectra were observed when the samples were kept in the dark. Interestingly, when irradiation of **1b** was conducted at higher concentrations, we also observed formation of a weak band at ∼640 nm (Figure S39), reminiscent of the phototruncated Cy5 species reported recently.^58^ These observations already indicated that the photochemistry of **1a** and **1b** likely proceeds through different mechanisms.

As anticipated, the fluorescence of coumarin payload **2** was quenched when it was caged within **1a**–**b**. Upon irradiation with NIR light in HEPES (pH 7.4, 20 mM, 10% DMF), 12 and 10-fold increase of the fluorescence intensity at ∼453 nm was observed for **1a** and **1b**, respectively, which perfectly matched the emission spectrum of the free payload **2** (Figures S40–S42). The control samples left in the dark show no such increase. We then quantified the chemical yield of the uncaging of **2** and the two isomers showed distinct behavior, as observed previously by absorption spectroscopy (Figure 2C,F–G). While **1a** released **2** in 43 ± 1% yield, with the majority of the release being finished after 10 min, **1b** liberated payload **2** only sluggishly in 11 ± 2% chemical yield.

The emission profile of the Cy7 skeleton upon photouncaging from **1a** was subsequently examined to gain insights into its fate. In all solvents systems [MeOH, HEPES with or without bovine serum albumin, Dulbecco’s modified Eagle’s medium (DMEM)], the uncaging is accompanied by a blueshift of the emission maxima of the Cy7 scaffold and a concomitant decrease of the intensity (Figures S43–S48). The latter process depends on the concentration of oxygen and corresponds to the photooxidation of the Cy7 scaffold. The observed blueshift of the emission spectrum matched that of the hypothesized photoproduct **10** formed by direct bond scission between **1a** and the payload (Figures 2A, S65, and S66). Interestingly, in DMEM cell culture media bubbled by argon, we initially observed an increase in Cy7 emission followed by its rapid decrease. This suggests that in a complex biological environment, the formation of **10** can outcompete the photooxidation of the Cy7 scaffold.

We further corroborated the uncaging of **2** by NIR light by ^1^H NMR spectroscopy (Figures S49–S55) and the chemical yields are in great agreement with those determined by emission spectroscopy. Irradiation of **1b** in CD_3_OD under ambient and oxygen-free conditions led to uncaging of **2** only in 12 and <2%, respectively. This reaffirms the earlier observation that *S*-thiocarbamate **1b** exhibits completely different photochemistry from *O*-thiocarbamate **1a** and shows that it proceeds only in the presence of oxygen. In contrast, the irradiation of **1a** at 810 nm under ambient and oxygen-free conditions led to the liberation of payload **2** in 52 and 47% chemical yields, respectively. In the former case, the uncaging was accompanied by the complete disappearance of the cyanine scaffold, consistent with ^1^O_2_-mediated uncaging, whereas in the absence of oxygen a concomitant formation of another cyanine photoproduct **11** was observed. This photoproduct, formed by capturing the putative cation **9** by CD_3_OD, confirms that the expulsion of COS from thiocarbamate **8** produced from **1a** is sufficient to drive the direct bond scission of the C–O bond. Consequently, our results indicate that the uncaging of payload **2** occurs likely through a combination of photooxidative and direct uncaging mechanisms (Scheme S4), aligning with our recent report.^40^ As a result, the operation of **1a** does not strictly rely on oxygen, rendering it suitable for application in both normoxic and hypoxic biological environments.

Next, we determined the amount of H_2_S produced by photouncaging using the established methylene blue (MB) assay (Figures 2C, S59, and S60).^59,60^ H_2_S is known to be oxidized by ROS species such as ^1^O_2_ generated by a self-sensitization, resulting in a peaking H_2_S concentration^61,62^ which complicates its quantification under irradiation. To ameliorate this, we performed the MB analysis using deaerated solutions containing zinc acetate which traps the H_2_S as ZnS. The irradiation of **1a**–**b** (50–150 μM) in HEPES (20 mM, pH 7.4, with 1% of MeOH and 5% of MeCN, respectively) in the presence of CA (1 μg/mL) at 810 nm generated H_2_S in 47 and 15% chemical yields, respectively, whereas no uncaging of H_2_S was observed for the control samples kept in the dark, or in the absence of CA. These values are in excellent agreement with the chemical yields of uncaged **2** determined by the emission and ^1^H NMR spectroscopies (Figure 2C), strongly suggesting that the uncaging of both species is linked to the same photochemical process. Unfortunately, the construction of a time profile of uncaged **2** and H_2_S from the very same sample was not possible due to different concentrations required for the emission spectroscopy studies (∼1.6–1.8 μM) and MB assay (∼50–150 μM). Moreover, since we cannot directly quantify COS, and the rate of its conversion into H_2_S depends on the concentration of CA, such a direct comparison would paint an inaccurate picture. We anticipated that both **1a** and **1b** would localize in mitochondria due to their lipophilic cationic nature, but because of the modest uncaging performance of *S*-thiocarbamate isomer **1b**, we focused on the *O*-thiocarbamate **1a** in the subsequent biological studies.

Both derivatives, *N*-methyl-substituted **1a** and *N*-alkyl sulfonate **1c**, as well as the corresponding photoproducts generated by their exhaustive irradiation showed minimal toxicity in HeLa cells up to 2.5 and 100 μM concentrations, respectively (Figures S61 and S62). Given the concentrations of H_2_S in mammalian tissue is typically lower (10 nM–3 μM),^63,64^ these photocages are suitable for the concurrent delivery of H_2_S and drug-like payloads in a biological context.

## Bioimaging Experiments

**Figure 3:**
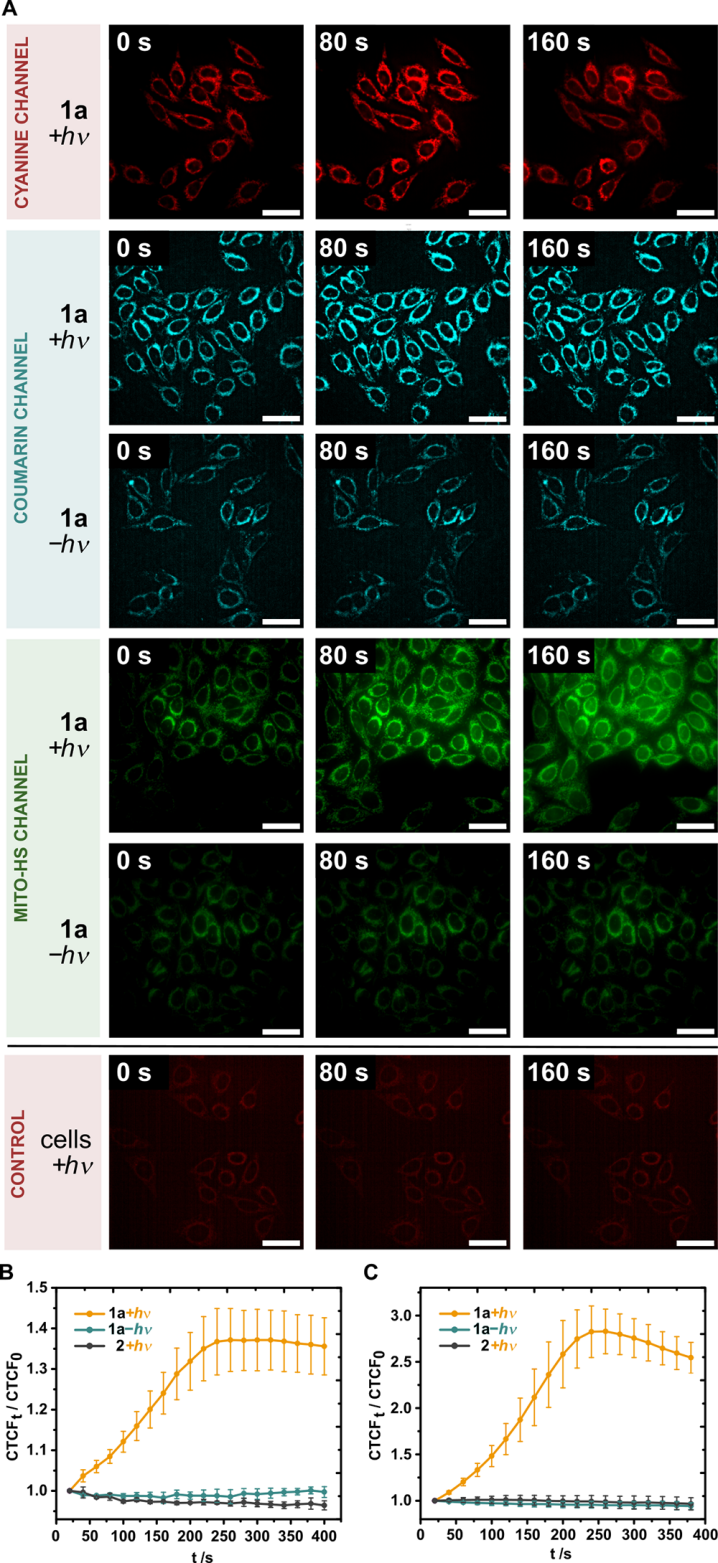
(A) Live-cell imaging of HeLa cells treated with **1a** (2 μM) in the presence or absence of irradiation at 747 nm at indicated time intervals, and the control experiment with nontreated HeLa cells (cyanine channel). (B,C) Plot of CTCF enhancement in HeLa cells in the presence (orange) or absence (blue) of irradiation at 747 nm in the coumarin payload **2** (B) and Mito-HS (C) channels. Average of 20–40 cells and a standard deviation of the mean are given. Scale bar is 40 μm.

Encouraged by these results, we set forth to investigate the internalization and photouncaging performance of **1a** within live HeLa cells using fluorescence microscopy. Our objective was twofold: (a) to establish that both H_2_S and the payload are liberated upon exposure of the cells to the NIR light and (b) confirm that it happens concurrently at a subcellular level within a specific organelle. To accomplish these goals, we used MitoTracker Deep Red and established Mito-HS^65^ as localization and mitochondria-targeted H_2_S probes, respectively. HeLa cells were incubated with photocage **1a** and subsequently irradiated at 747 nm with the excitation light source of the microscope (Figure 3A). Only low intensities of excitation light sources were used for the imaging to exclude potential phototoxicities. The evolution of the fluorescence intensity over time was quantified by relative corrected total cell fluorescence (CTCF, Figure 3B,C).

Indeed, a significant increase of the fluorescence intensity in the payload **2** (∼1.4-fold) and Mito-HS (∼2.7-fold) channels was observed in HeLa cells upon irradiation with NIR light, confirming the concurrent uncaging of **2** and H_2_S. The magnitude of the fluorescence increase of **2** depended on the initial concentration of photocage **1a** (Figure S63), clearly demonstrating that its concentration delivered to the mitochondria can be tuned by the concentration used in incubation. On the other hand, irradiation of **1a** with light at 477 or 555 nm did not trigger significant uncaging of payload **2** or H_2_S (Figures S64 and S65). In control experiments, in the absence of irradiation or for cells containing only free **2** or Mito-HS without photocage **1a**, no significant increase in fluorescence was observed (Figure S66).

Interestingly, we also observed an initial increase in fluorescence intensity in the Cy7 channel (∼1.6-fold), followed by its gradual bleaching (Figure S67). This is in excellent agreement with the increase of emission observed upon irradiation of **1a** in DMEM cell culture media made by emission spectroscopy (vide supra, Figures S44 and S45). We hypothesize that in the biological settings, the payload **2** is uncaged mostly via direct uncaging pathway which likely outcompetes the photooxidative pathway,^40^ resulting in the initial formation of the more emissive Cy7 photoproduct **10**. Subsequent decrease in the emission is due to the photobleaching of **1a** and **10**.

**Figure 4:**
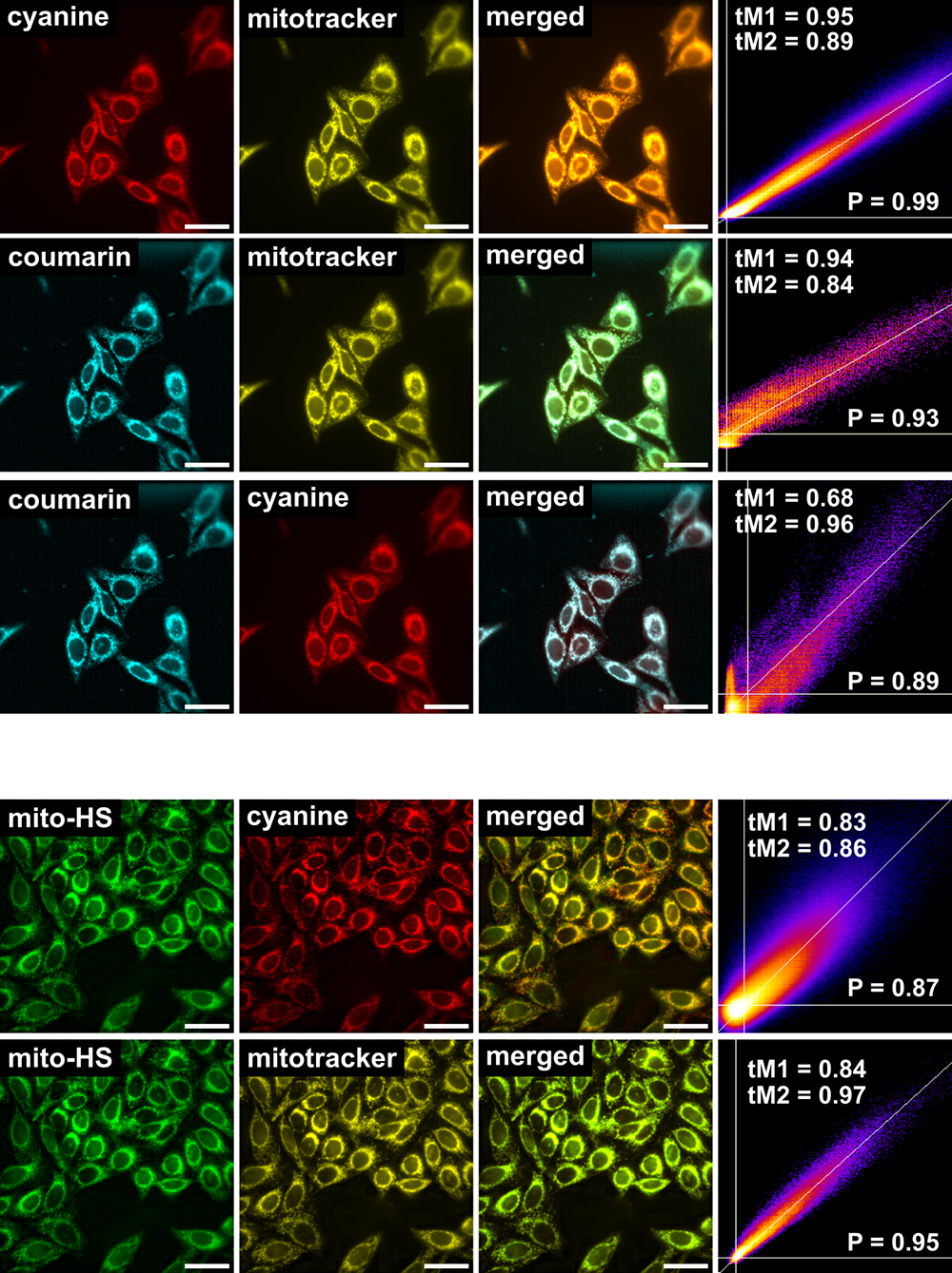
Live-cell imaging of mitochondria-localized, concurrent uncaging of **2** and H_2_S in HeLa cells with NIR light. (Top) Co-localization of the photocage **1a** (2 μM), uncaged payload **2**, and MitoTracker DR (50 nM). (bottom) Co-localization of **1a** (2 μM), Mito-HS (5 μM), and MitoTracker DR (50 nM). The cells were incubated with **1a**, MitoTracker DR, and with or without Mito-HS and imaged after the uncaging was complete (irradiation at 747 nm for 180 s). Scale bar represents 40 μm.

Yet to accomplish the second goal, the challenge lay in having a total of four fluorophores (including coumarin payload **2** and Cy7 photocage scaffold), significantly complicating multiplexed imaging. Because the emission of payload **2** and absorption of Mito-HS significantly overlap, we decided to image them separately into two distinct experiments, i.e., with and without Mito-HS (Figure 4). We first tracked photocage **1a** which was colocalized with MitoTracker DR with an excellent Pearson’s correlation coefficient (PCC) of 0.99. The fluorescence of the uncaged payload **2** was then imaged in the absence of Mito-HS, and it colocalized with both **1a** and the MitoTracker DR with high PCCs (0.89 and 0.93, respectively). Subsequently, we assessed only the fluorescence increase of Mito-HS originating from uncaged H_2_S, which also displayed high overlap with both parent **1a** and the MitoTracker with high PCCs (0.87 and 0.95, respectively). Through the colocalization with a MitoTracker DR as probe common to both experiments, we conclusively confirm that **2** and H_2_S are uncaged concurrently in mitochondria using NIR light as a trigger.

## Conclusions

In conclusion, we present a dual-action photocage capable of concurrently delivering H_2_S and an amine payload with subcellular precision in mitochondria of live human cells using tissue-penetrating light as a trigger. This system features a self-reporting mechanism for H_2_S release, utilizing the fluorescence of the coumarin payload as a proxy reporter, and its substitution with a drug payload will allow a simultaneous delivery of H_2_S–drug pairs with high resolution in time and space. We believe this approach can be extended to target other cellular compartments by exploiting the versatile chemistry of cyanines and incorporating groups that specifically target organelles, such as the ER or Golgi apparatus. We anticipate that these dual-action photocages will enable the harnessing and exploration of synergistic effects between H_2_S and drugs, paving the way for innovative therapeutic strategies.

## Methods

### Photolysis and Stability in the Dark

A solution of photocage **1a**–**b** (*c* ∼ 2 × 10^–5^ M, 3000 μL, *A* < 1.5) in aerated HEPES (pH 7.4, 20 mM with 10% DMF) was stirred and left to equilibrate for 2–3 min at 20 °C. Afterward, the sample was irradiated with light-emitting diodes (LEDs) at 820 nm (∼50 mW/cm^2^), and the progress of the irradiation was monitored at the given time intervals by UV–vis spectrometry using a diode-array spectrophotometer. The total irradiation time was selected to reach >95% conversion and to obtain minimum of 15 experimental points. The procedure was repeated three times. The dark stability of **1a**–**b** was recorded by using the same procedure with the exclusion of the irradiation source.

### Chemical Yield of H_2_S Release with Methylene Blue Assay

Cyanine **1a**: A solution of cyanine **1a** (1 mL, *c* = 150 × 10^–6^ M) in HEPES buffer (pH 7.4, 20 mM) with 1% MeOH was placed in a 1.0 cm quartz PTFE screw-cap cuvette equipped with a stirring bar. A solution of CA (from bovine erythrocytes, 3500 U/mg, 1 mg/mL, and 150 μL) was added to the sample solution. Zinc acetate (300 μL, 1% w/v) was added to prevent oxidation of the H_2_S formed and precipitate H_2_S as ZnS, respectively. The solution was stirred and irradiated with LED array at 820 nm (∼300 mW/cm^2^) for 1 h and then *N*,*N*-dimethyl-*p*-phenylenediamine sulfate (200 μL, 20 mM in 7.2 M aq. HCl), FeCl_3_ (200 μL, 30 mM in 1.2 M aq. HCl), and water (1.45 mL) were added. The samples were incubated at room temperature for 1 h, and the absorbance at 670 nm was recorded and used for H_2_S concentration calculation using a calibration curve for NaSH (Figure S59).

### Imaging of the Uncaging in Live HeLa Cells

The cells (prepared according to the Supporting Information) were placed in a light microscope and kept at 37 °C under a 5% CO_2_ atmosphere throughout the duration of experiments. The cells were visualized using Leica DMi8 fully motorized, inverted microscope with a fluorescence light source Leica LED8 at either 40× (for statistical calculations in Figure 3B,C) or at 63× (Figures 3A and 4) magnification, using bright field channel (40 ms), coumarin fluorescence channel (exc. 390 nm, 18% intensity, exposure 150 ms, 420–450 nm detection), Mito-HS channel (exc. 440 nm, 7% intensity, exposure 100 ms, 462–484 nm detection), MitoTracker Deep Red channel (exc. 635 nm, 3% intensity, exposure 100 ms, 666–724 nm detection), and NIR channel (exc. 747 nm, 10% intensity, exposure 1 s, 770–850 nm detection) in 20 cycles. Procedure was repeated with three other excitation wavelengths: 475 nm (10% intensity, exposure 1 s, 506–532 nm detection), 555 nm (10% intensity, exposure 1 s, 581–607 nm detection), and 635 nm (10% intensity, exposure 1 s, 666–724 nm detection) in 20 cycles. The images were processed using ImageJ. The CTCF was calculated from the fluorescence intensity by subtraction of the background intensity in the vicinity of the cells. This was performed for all channels and each time point between 0 and 400 s. Each time point was calculated using 30–50 living cells for experiments with **1a** under irradiation or in the dark, and 20< cells for control experiment involving no incubation or incubation with free coumarin **2** or only Mito-HS. The experiments were performed using three technical and biological replicates. The enhancement was calculated as a ratio of CTCF_t_ in time (*t*), and the initial CTF_0_ was plotted against time. The average of the enhancement and standard deviation of the mean are depicted.

## Supporting Information Available

Synthetic routes; characterization of the intermediates and final molecules; photophysical and photochemical studies and experiments; quantification of the uncaging; cell viability studies; and in vitro uncaging experiments followed by fluorescence microscopy (PDF)
Live-cell imaging of the Cy7 channel (MP4)
Live-cell imaging of the payload **2** channel (MP4)
Live-cell imaging of the Mito-HS channel (MP4)
