# Supplementary material for: Concurrent Subcellular Delivery of Hydrogen Sulfide and a Payload with Near-Infrared Light
Source: JACS Au. 2024 Jul 5;4(7):2687–94. doi: 10.1021/jacsau.4c00445 (PMC11267537; doi:10.1021/jacsau.4c00445)
Supplement: Supplementary file 1 — au4c00445_si_001.pdf [file au4c00445_si_001.pdf]

Supporting information

Concurrent Subcellular Delivery of Hydrogen Sulfide and A Payload with Near-Infrared Light

Katarzyna Hanc,[1] Hana Janeková,[1] Peter Štacko[1,]*

[1] Department of Chemistry, University of Zurich, Winterthurerstrasse 190, CH-8057 Zurich, Switzerland

*Email: peter.stacko@uzh.ch

Table of Contents

Materials and Methods	S2
Synthesis	S2-S7
Photophysical and Photochemical Measurements Methodology	S7-S8
Methodology of Biological Experiments	S9-S10
NMR Spectroscopy	S11-S36
UV-Vis Absorption and Emission Spectroscopy	S37
Photophysical and Photochemical Measurements	S46–52
Biological experiments	S53
References	S57

Materials and Methods

Reagents and solvents of the highest purity available were used as purchased, or they were purified/dried using standard methods when necessary. The intermediates **7a-b** were synthesized according to the published procedures[1] or purchased from standard suppliers (Merck, TCI, Across Organics, etc.).

Column chromatography was performed using silica gel (230−400 mesh). ^{1}H NMR spectra were recorded on 400 or 500 MHz spectrometers; ^{13}C NMR spectra were obtained on 125 MHz instruments in $CDCl_3$, CD_3OD, and d_6-DMSO. ^{1}H chemical shifts are reported in ppm relative to $CDCl_3$ (δ = 7.26 ppm), CD_3OD (δ = 3.31 ppm) and d_6-DMSO (δ = 2.50 ppm) as an internal reference. ^{13}C chemical shifts are reported in ppm with $CDCl_3$ (δ = 77.67 ppm), CD_3OD (δ = 49.30 ppm) and d_6-DMSO (δ = 39.52 ppm) as internal references. Deuterated solvents were kept under nitrogen atmosphere.

Absorption spectra and molar absorption coefficients were obtained on a UV-vis spectrometer with matched 1.0-cm quartz cells. Fluorescence and excitation spectra were measured using a fluorescence spectrometer in a 1.0 cm quartz fluorescence cuvette at 20 °C. The sample concentrations were adjusted to keep the absorbance below 0.2 at the corresponding excitation wavelength. Each sample was measured five times, and the spectra were averaged. Emission and excitation spectra were normalized and corrected by the photomultiplier sensitivity function using correction files supplied by the manufacturer.

The exact masses of the synthesized compounds were obtained using a triple quadrupole electrospray ionization mass spectrometer in a positive or negative mode coupled with direct-inlet.

Synthesis of the Intermediates and Photocages

Benzyl (4-methyl-2-oxo-2*H*-chromen-7-yl)carbamate (12)

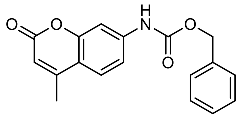

To the mixture of 7-amino-4-methylcoumarin (1.5 g, 8.56 mmol, 1 eq) in THF (20 mL) at 0-5°C, benzyl chloroformate (2.4 mL, 17.1 mmol, 2 eq) and $NaHCO_3$ (2.16 g, 25.7 mmol) were added, and mixture was then stirred for 48 h at room temperature. The solvent was evaporated under reduced pressure and EtOAc (20 mL) was added. Organic phase was washed with sat. aq. solution of $NaHCO_3$ (2×20mL) and brine (20mL). Organic phase was separated, and the undissolved solids were filtered off. Filtrate was dried with $MgSO_4$ and the volatiles evaporated under reduced pressure. The crude product was triturated with CH_2Cl_2 to obtain the final product. Yield: 1.97 g (74%). Colorless solid. ^{1}H NMR (400 MHz, d_6-DMSO) δ 10.28 (s, 1H), 7.69 (d, J = 8.7 Hz, 1H), 7.55 (d, J = 2.1 Hz, 1H), 7.51 – 7.26 (m, 6H), 6.23 (t, J = 1.3 Hz, 1H), 5.19 (s, 2H), 2.38 (d, J = 1.3 Hz, 3H). The analytical data are in agreement with the reported values.[2]

Benzyl methyl(4-methyl-2-oxo-2H-chromen-7-yl)carbamate (13).

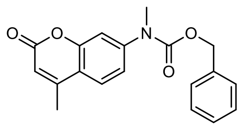

NaH (306 mg, 7.64 mmol, 1.2 eq) was added to a solution of **12** (1.97 g, 6.37 mmol, 1 eq) in DMF (20 mL) at 0-5°C. The mixture was stirred for 20 min at room temperature, cooled back to 0-5°C and MeI (0.6 mL, 9.55 mmol, 1.5 eq) was added. After stirring for 1.5h at room temperature water (20 mL) and EtOAc (20 mL) were added and separated organic phase was washed with brine (20 mL) and dried with anhydrous $MgSO_4$. The organic phase was filtered, and the solvents

were evaporated under reduced pressure. The crude product was purified by column chromatography (SiO_2, isocratic pentane/EtOAc/ CH_2Cl_2 – 6:2:2, then pentane/EtOAc/ CH_2Cl_2/acetone – 8:5:5:2) to afford the product. Yield: 1.94 g (94%). Yellowish solid. ^{1}H NMR (400 MHz, d_6-DMSO) δ (ppm) 7.74 (d, J = 8.6 Hz, 1H), 7.44 (d, J = 2.1 Hz, 1H), 7.40 (dd, J = 8.6, 2.2 Hz, 1H), 7.38 – 7.28 (m, 5H), 6.36 (d, J = 1.4 Hz, 1H), 5.16 (s, 2H), 3.33 (s, 3H), 2.42 (d, J = 1.3 Hz, 3H). ^{13}C NMR (400 MHz, d_6-DMSO) δ (ppm) 159.80, 154.20, 153.07, 152.92, 146.01, 136.35, 128.45, 127.99, 127.72, 125.39, 121.02, 116.81, 113.53, 112.30, 67.04, 36.99, 18.04. HRMS (ESI+) calcd. for [$C_{19}H_{18}NO_4^+$] 324.1231, found 324.1245.

4-Methyl-7-(methylamino)-2*H*-chromen-2-one (2)

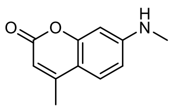

Pd/C (10%, 96 mg, 0.09 mol, 0.015 eq) was added to the solution of **9** (1.94 g, 4.79 mmol, 1 eq) in EtOAc (30 mL) and reaction mixture was stirred under H_2 atmosphere for 3 h at room temperature. MeOH (25 mL) was added, and reaction mixture was slightly heated up to dissolve all the precipitated solid. The mixture was filtered through celite pad and solvent was concentrated under reduced pressure to obtain the product. Yield: 1.09 g (96%). Colorless solid. ^{1}H NMR (400 MHz, d_6-DMSO) δ (ppm) 7.44 (d, J = 8.7 Hz, 1H), 6.86 – 6.64 (m, 1H), 6.58 (dd, J = 8.8, 2.3 Hz, 1H), 6.35 (d, J = 2.3 Hz, 1H), 6.07 – 5.81 (m, 1H), 2.74 (d, J = 4.9 Hz, 3H), 2.31 (d, J = 1.1 Hz, 3H). The analytical data are in agreement with the reported values.[3]

***N*-Methyl-*N*-(4-methyl-2-oxo-2*H*-chromen-7-yl)-1*H*-imidazole-1-carbothioamide (3a).**

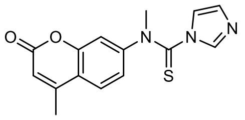

Coumarin **2** (250 mg, 1.32 mmol, 1 eq) and TCDI (706 mg, 3.96 mmol, 3 eq) were dissolved in THF (10 mL) and reaction mixture was stirred at 60°C for 20 h. The solvent was evaporated under reduced pressure and the crude product was purified by column chromatography (SiO_2, isocratic CH_2Cl_2Acetone – 9:1) to obtain the product. Yield: 34 mg (85%). Yellowish solid. ^{1}H NMR (400 MHz, d_6-DMSO) δ (ppm) 7.83 (t, J = 1.1 Hz, 1H), 7.73 (d, J = 8.5 Hz, 1H), 7.48 (d, J = 2.1 Hz, 1H), 7.22 (dd, J = 8.5, 2.2 Hz, 1H), 6.77 (t, J = 1.2 Hz, 1H), 6.43 (q, J = 1.3 Hz, 1H), 5.75 (s, 1H), 3.80 (s, 3H), 2.40 (d, J = 1.4 Hz, 3H). ^{13}C NMR (500 MHz, d_6-DMSO) δ (ppm) 178.30, 159.30, 153.18, 152.66, 147.07, 137.62, 128.84, 126.53, 121.18, 120.19, 118.79, 114.77, 113.47, 46.19, 18.05. HRMS (ESI+) calcd. For [$C_{15}H_{14}N_3O_2S^+$] 300.0802, found 300.0805.

4-Nitrophenyl methyl(4-methyl-2-oxo-2*H*-chromen-7-yl)carbamate (3b).

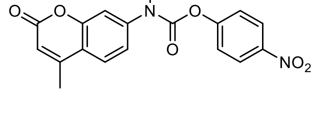

Coumarin **2** (150 mg, 0.79 mmol, 1 eq) was dissolved in anhydrous acetonitrile (5 mL), DIPEA (0.4 mL, 2.38 mmol, 3 eq) and 4-nitrophenyl chloroformate (310 mg, 2.38 mmol, 3 eq) were added and the reaction mixture was refluxed for 2h. Solvent was evaporated under reduced pressure, the residue was dissolved in CH_2Cl_2 (10 mL), washed with brine (2×10 mL) and dried with $MgSO_4$. Solvent was evaporated under reduced pressure and crude product was washed with MeOH/ EtOAc – 1:1 (3×3mL) to obtain the product. Yield: 329 mg (62%). Colorless solid. ^{1}H NMR (400 MHz, d_6-DMSO) δ (ppm) 8.30 (d, J = 8.9 Hz, 2H), 7.82 (d, J = 8.6 Hz, 1H), 7.69 – 7.60 (m, 1H), 7.56 (dd, J = 8.6, 2.2 Hz, 1H), 7.52 (d, J = 8.8 Hz, 2H), 6.41 (s, 1H), 3.45 (s, 3H), 2.45 (s, 3H). ^{13}C NMR (100 MHz, d_6-DMSO) δ (ppm) 159.74, 155.82, 153.14, 152.91, 151.85, 145.31, 144.77, 125.81, 125.19, 123.08, 122.76, 121.75, 117.76, 114.02, 113.31, 37.79, 18.12. HRMS (ESI+). calcd. for [$C_{18}H_{15}N_2O_6^+$] 355.0925, found 355.0923.

1-(Pyridin-4-yl)ethyl ethanethioate (4b).

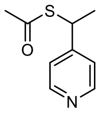

Alcohol **4a** (1 g, 7.96 mmol, 1 eq) was dissolved in chloroform (10 mL) and $SOCl_2$ (1.46 mL, 19.9 mmol, 2.5 eq) was added dropwise at 0°C. Ice bath was removed, and reaction mixture was refluxed for 6 h. Reaction mixture was cooled down, quenched by sat. aq. $NaHCO_3$ (10 mL) and the organic phase was washed with brine (10mL). The organic phase was separated, dried with anhydrous $MgSO_4$, filtered and solvent evaporated under reduced pressure. Crude compound was purified on a short column (SiO_2, isocratic CH_2Cl_2/MeOH – 95:5) to obtain the intermediate chloride which was unstable and therefore used immediately without further purification. Yield 938 mg (83%). Brownish oil. The chloride (930 mg, 6.57 mmol, 1eq) was dissolved in acetone (10 mL), potassium thioacetate (2.25 g, 19.7 mmol, 3.0 eq) was added and the reaction mixture was refluxed for 4 h. The solution was cooled to room temperature, water (10 mL) was added, and the mixture was extracted with EtOAc (3×10mL). Organic phase was separated, dried with anhydrous $MgSO_4$, filtered, and concentrated under reduced pressure to obtain 1.07 g of the crude compound. Crude compound was used later without further purification. Yield: 1.07 g (90%). Orange oil. ^{1}H NMR (400 MHz, d_6-DMSO) δ (ppm) 8.57 – 8.44 (m, 2H), 7.43 – 7.29 (m, 2H), 4.63 (q, J = 7.2 Hz, 1H), 2.31 (s, 3H), 1.57 (d, J = 7.2 Hz, 3H). ^{13}C NMR (100 MHz, d_6-DMSO) δ (ppm) 194.13, 151.44, 149.85, 122.36, 41.23, 30.34, 20.88. HRMS (ESI+) calcd. for [$C_9H_{12}NOS^+$] 182.0635, found 182.0628.

***O*-(1-(Pyridin-4-yl)ethyl) methyl(4-methyl-2-oxo-2*H*-chromen-7-yl)carbamothioate (5a).**

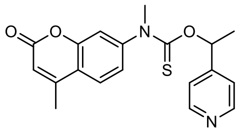

4a (200 mg, 0.68 mmol, 1 eq) was dissolved in anhydrous DMF (3 mL) and solution was cooled down to -5°C in ice/NaCl bath. NaH (27 mg, 0.67 mmol, 1 eq) was added and the reaction mixture was stirred for 20 min at room temperature. A solution of **3a** (200 mg, 0.67 mmol, 1 eq) in anhydrous DMF (3 mL) prepared in advance was then added, and the reaction mixture was stirred at -5°C for 2 h, followed by 18 h at room temperature. Sat. aq. solution of $NaHCO_3$ (5 mL) was added and mixture was extracted with EtOAc (3×10 mL). Organic phase was separated, dried over anhydrous $MgSO_4$, filtered and solvent was evaporated under reduced pressure. The crude product was purified by column chromatography (SiO_2, gradient MeOH in CH_2Cl_2 from 1 to 3%) to obtain the product. Yield: 121 mg (51%). Greenish amorphous solid. ^{1}H NMR (400 MHz, d_6-DMSO) δ (ppm) 8.51 (d, J = 5.0 Hz, 2H), 7.85 (d, J = 8.5 Hz, 1H), 7.51 (d, J = 2.1 Hz, 1H), 7.40 (dd, J = 8.5, 2.1 Hz, 1H), 7.21 (s, 2H), 6.45 (d, J = 1.4 Hz, 1H), 6.38 (q, J = 6.6 Hz, 1H), 3.59 (s, 3H), 2.46 (d, J = 1.3 Hz, 3H), 1.47 (d, J = 6.0 Hz, 3H). ^{13}C NMR (125 MHz, d_6-DMSO) δ (ppm) 186.62, 159.60, 153.22, 152.86, 150.09, 149.74, 126.05, 122.80, 120.52, 118.68, 114.52, 77.43, 21.72, 18.14. HRMS (ESI+) calcd. for [$C_{19}H_{19}N_2O_3S^+$] 355.1111, found 355.1108.

***S*-(1-(Pyridin-4-yl)ethyl) methyl(4-methyl-2-oxo-2*H*-chromen-7-yl)carbamothioate (5b).**

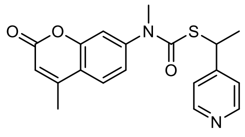

4b (250 mg, 1.38 mmol, 1 eq) was dissolved in anhydrous MeOH (3 mL). The solution was cooled down to 0 °C and NaOMe (71 mg, 1.24 mmol, 0.9 eq) was added. The mixture was stirred at room temperature under N_2 atmosphere for 30 min, and then added dropwise to a suspension of the **3b** (350 mg, 1.00 mmol, 0.73 eq) in anhydrous MeOH (2 mL). After stirring for 2 h at room temperature, pentane was added and precipitated solid was filtered off. Filtrate was evaporated under reduced pressure and the crude product was purified by column chromatography (SiO_2, isocratic CH_2Cl_2/MeOH – 95:5) to obtain the product. Yield: 127 mg (26%). Yellowish solid. ^{1}H NMR (400 MHz, d_6-DMSO) δ (ppm) 8.53 – 8.43 (m, 2H), 7.82 (d, J = 8.5 Hz, 1H), 7.50 (d, J = 2.1 Hz, 1H), 7.38 (dd, J = 8.4, 2.1 Hz, 1H), 7.39 – 7.33 (m, 2H), 6.44 (d, J = 1.4 Hz, 1H), 4.57 (q, J = 7.2 Hz, 1H), 3.27 (s, 3H), 2.44 (d, J = 1.3 Hz, 3H), 1.54 (d, J = 7.2 Hz, 3H). ^{13}C

NMR (126 MHz, d_4-MeOH) δ (ppm) 168.56, 162.32, 155.61, 155.03, 154.61, 150.16, 146.25, 127.29, 125.13, 124.18, 121.04, 117.22, 115.93, 44.83, 38.30, 21.68, 18.61. HRMS (ESI+) calcd. For $[C_{19}H_{19}N_2O_3S^+]$ 355.1111, found 355.1089.

1-(2,4-Dinitrophenyl)-4-(1-((methyl(4-methyl-2-oxo-2*H*-chromen-7-yl)carbamothioyl)oxy)ethyl)5yridine-1-ium trifluoromethanesulfonate (6a).

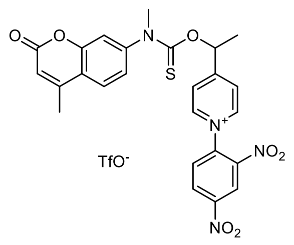

5a (80 mg, 0.23 mmol, 1 eq) was dissolved in anhydrous acetonitrile (2 mL), flask was covered with aluminum foil, dinitrophenyl triflate (71 mg, 0.23 mmol, 1 eq) was added and mixture was stirred at 30 °C for 18 h under N_2 atmosphere under exclusion of light. The solvent was evaporated, Et_2O (5 mL) was added and precipitated solid was washed with Et_2O (3×5 mL) filtered and dried under reduced pressure to obtain the product. Yield: 137 mg (91%). Beige powder. ^1H NMR (400 MHz, d_6-DMSO) δ (ppm) 9.29 (d, J = 6.5 Hz, 2H), 9.12 (d, J = 2.6 Hz, 1H), 8.92 (dd, J = 8.7, 2.5 Hz, 1H), 8.39 (d, J = 8.7 Hz, 1H), 8.24 (d, J = 6.4 Hz, 2H), 7.87 (d, J = 8.4 Hz, 1H), 7.55 (d, J = 2.1 Hz, 1H), 7.45 (dd, J = 8.5, 2.1 Hz, 1H), 6.72 (q, J = 6.8 Hz, 1H), 6.42 (d, J = 1.5 Hz, 1H), 3.67 (s, 3H), 2.47 (d, J = 1.3 Hz, 3H), 1.66 (d, J = 6.7 Hz, 3H). ^{13}C NMR (125 MHz, d_6-DMSO) δ (ppm) 163.93, 159.56, 153.26, 152.82, 149.16, 146.03, 143.13, 138.51, 131.92, 130.18, 126.17, 124.52, 124.22, 121.96, 121.45, 119.39, 118.88, 116.83, 114.64, 76.90, 64.92, 21.29, 18.14, 15.17. HRMS (ESI+) calcd. for $[C_{25}H_{21}N_4O_7S^+]$ 521.1126, found 521.1122.

1-(2,4-Dinitrophenyl)-4-(1-((methyl(4-methyl-2-oxo-2*H*-chromen-7-yl)carbamoyl)thio)ethyl)pyridin-1-ium trifluoromethanesulfonate (6b).

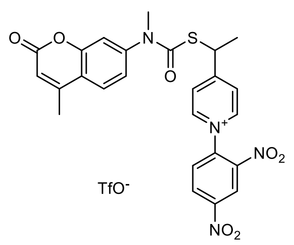

5b (127 mg, 0.36 mmol, 1 eq) was dissolved in anhydrous acetonitrile (2 mL), flask was covered with aluminum foil and dinitrophenyl triflate (125 mg, 0.23 mmol, 1.1 eq) was added and mixture was stirred at 40°C for 18 h under N_2 atmosphere. Solvent was evaporated, Et_2O was added and precipitated solid was washed with Et_2O (3 x 5 mL), filtered, and dried under reduced pressure to obtain the product. Yield: 226 mg (94%). Beige powder. ^1H NMR (400 MHz, d_6-DMSO) δ (ppm) 9.27 (d, J = 6.5 Hz, 2H), 9.12 (d, J = 2.5 Hz, 1H), 8.96 (dd, J = 8.7, 2.6 Hz, 1H), 8.42 (t, J = 7.7 Hz, 3H), 7.87 (d, J = 8.5 Hz, 1H), 7.55 (d, J = 2.1 Hz, 1H), 7.43 (dd, J = 8.4, 2.1 Hz, 1H), 6.48 (d, J = 1.5 Hz, 1H), 4.96 (q, J = 7.2 Hz, 1H), 3.30 (s, H), 2.46 (d, J = 1.3 Hz, 3H), 1.66 (d, J = 7.3 Hz, 3H). ^{13}C NMR (125 MHz, d_6-DMSO) δ (ppm) 166.56, 165.09, 159.41, 153.16, 152.71, 149.12, 145.78, 143.90, 143.12, 138.54, 131.95, 130.23, 130.14, 126.50, 126.37, 123.77, 121.95, 121.44, 119.51, 119.39, 115.82, 114.87, 43.23, 37.86, 19.87, 18.13. HRMS (ESI+) calcd. for $[C_{25}H_{21}N_4O_7S^{+]}$ 521.1126, found 521.1122.

General Procedure for Synthesis of Cyanines1a-c.

The corresponding Zincke salt **5a-b** (1 eq.) and heterocycle **7a-b** (3 eq) were suspended in a solution of AcOK (6 eq) in MeCN (15 mL/mmol). The reaction mixture was stirred at rt for 1 8h in a flask covered with aluminum foil. The volatiles were evaporated under reduced pressure, CH_2Cl_2 (3 mL/mmol) was added, and the organic phase was washed with H_2O (3×4 mL/mmol). Solvent was evaporated under reduced pressure and the crude product was purified by column chromatography (SiO_2, isocratic CH_2Cl_2/MeOH – 97:3, unless stated differently).

5-Methoxy-2-((1*E*,3*Z*,5*E*)-7-((*E*)-5-methoxy-1,3,3-trimethylindolin-2-ylidene)-4-(1-((methyl(4-methyl-2-oxo-2*H*-chromen-7-yl)carbamothioyl)oxy)ethyl)hepta-1,3,5-trien-1-yl)-1,3,3-trimethyl-3*H*-indol-1-ium iodide (1a)

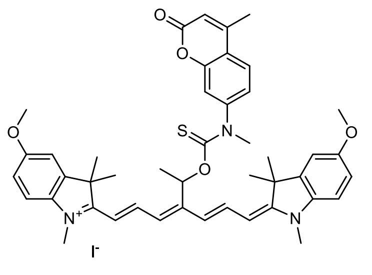

Prepared according to the general procedure from **6a** (120 mg, 0.18 mmol, 1 eq) and **7a** (178 mg, 0.54 mmol, 3 eq). Yield: 66 mg (42%). Dark green solid. ^{1}H NMR (400 MHz, *d*$_6$-DMSO) δ (ppm) 7.91 (t, J = 13.6 Hz, 2H), 7.86 (d, J = 8.5 Hz, 1H), 7.45 (d, J = 2.0 Hz, 1H), 7.39 (dd, J = 8.4, 2.1 Hz, 1H), 7.31 (d, J = 8.7 Hz, 2H), 7.25 (d, J = 2.5 Hz, 2H), 6.97 (dd, J = 8.7, 2.5 Hz, 2H), 6.69 (q, J = 6.7 Hz, 1H), 6.52 – 6.37 (m, 3H), 6.27 (d, J = 13.3 Hz, 2H), 3.81 (s, 6H), 3.61 (s, 3H), 3.57 (s, 6H), 2.45 (d, J = 1.3 Hz, 3H), 1.61 (d, J = 9.3 Hz, 12H), 1.51 (d, J = 6.4 Hz, 3H). ^{13}C NMR (125 MHz, *d*$_6$-DMSO) δ (ppm) 186.93, 170.46, 159.58, 157.61, 156.30, 153.15, 152.87, 142.67, 141.40, 136.50, 126.06, 122.53, 119.54, 119.39, 118.63, 114.48, 114.39, 113.41, 111.71, 109.07, 105.08, 75.70, 55.85, 54.91, 48.78, 31.47, 27.35, 27.24, 20.80, 18.10. HRMS (ESI+) calcd. for [$C_{45}H_{50}N_3O_5S^+$] 744.3466, found 744.3468.

Potassium (3-(5-methoxy-2-((1*E*,3*Z*,5*E*)-7-((*E*)-5-methoxy-3,3-dimethyl-1-(3-sulfonato-propyl)indolin-2-ylidene)-4-(1-((methyl(4-methyl-2-oxo-2*H*-chromen-7-yl)carbamothio-yl)oxy)ethyl)hepta-1,3,5-trien-1-yl)-3,3-dimethyl-3*H*-indol-1-ium-1-yl)propane-1-sulfona-te) monoiodide (1c)

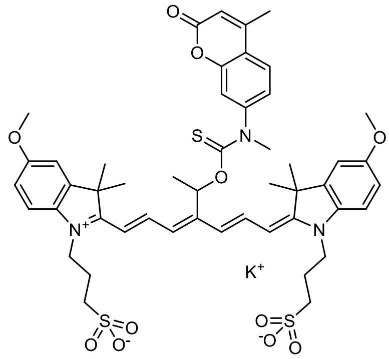

Prepared according to the general procedure from **6a** (50 mg, 0.08 mmol, 1 eq) and **7b** (70 mg, 0.22 mmol, 3 eq). Crude product was purified by column chromatography (SiO_2, isocratic CH_2Cl_2/MeOH – 8:2. Yield: 30 mg (42%). Dark green solid. ^{1}H NMR (500 MHz, *d*$_6$-DMSO) δ (ppm) 8.03 – 7.90 (m, 3H), 7.45 – 7.43 (m, 1H), 7.43 – 7.39 (m, 3H), 7.25 – 7.18 (m, 2H), 6.96 (dd, J = 8.7, 2.4 Hz, 2H), 6.69 (q, J = 6.7 Hz, 1H), 6.53 (d, J = 13.4 Hz, 2H), 6.48 (d, J = 13.2 Hz, 2H), 6.43 – 6.39 (m, 1H), 4.37 – 4.13 (m, 4H), 3.83 – 3.79 (m, 6H), 3.63 – 3.60 (m, 3H), 2.58 (t, J = 6.9 Hz, 4H), 2.46 (d, J = 1.6 Hz, 3H), 2.02 (p, J = 7.7 Hz, 4H), 1.63 (s, 6H), 1.61 (s, 6H), 1.53 (d, J = 6.0 Hz, 3H). ^{13}C NMR (126 MHz, *d*$_6$-DMSO) δ (ppm) δ 186.97, 169.95, 159.62, 157.55, 156.65, 153.15, 153.05, 142.82, 141.83, 135.81, 126.37, 122.80, 120.50, 119.71, 118.60, 114.34, 114.31, 113.54, 111.92, 109.08, 105.02, 75.73, 55.83, 54.91, 48.88, 47.90, 42.93, 27.56, 27.47, 23.63, 20.82, 18.17. HRMS (ESI+) calcd. for [$C_{49}H_{57}N_3O_{11}S_3$]: 959.3155, found 959.3156.

5-Methoxy-2-((1*E*,3*Z*,5*E*)-7-((*E*)-5-methoxy-1,3,3-trimethylindolin-2-ylidene)-4-(1-((methyl(4-methyl-2-oxo-2*H*-chromen-7-yl)carbamoyl)thio)ethyl)hepta-1,3,5-trien-1-yl)-1,3,3-trimethyl-3*H*-indol-1-ium iodide (1b)

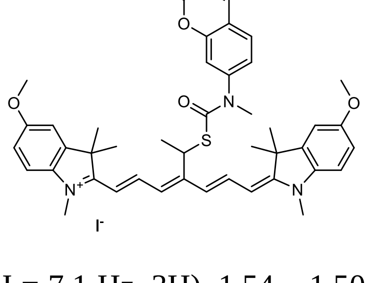

Prepared according to the general procedure from **6b** (100 mg, 0.15 mmol, 1 eq) and **7a** (148 mg, 0.45 mmol, 3 eq). Column chromatography SiO_2 isocratic CH_2Cl_2/MeOH 95:5. Yield: 46 mg (35%). Dark green solid. ^{1}H NMR (400 MHz, *d*$_6$-DMSO) δ (ppm) 7.94 – 7.80 (m, 3H), 7.53 (d, J = 2.1 Hz, 1H), 7.40 (dd, J = 8.5, 2.1 Hz, 1H), 7.30 (d, J = 8.7 Hz, 2H), 7.24 (d, J = 2.5 Hz, 2H), 6.97 (dd, J = 8.7, 2.5 Hz, 2H), 6.54 – 6.41 (m, 3H), 6.30 (d, J = 13.3 Hz, 2H), 5.04 (q, J = 6.9 Hz, 1H), 3.81 (s, 6H), 3.56 (s, 6H), 3.34 (s, 3H), 2.43 (d, J = 1.3 Hz, 3H), 1.62 (s, 6H), 1.60 (d, J = 7.1 Hz, 3H), 1.54 – 1.50 (m, 6H). ^{13}C NMR (125 MHz, *d*$_6$-DMSO) δ (ppm) 170.51, 166.14, 159.42, 158.91, 157.59,

153.09, 152.76, 144.03, 142.68, 141.72, 136.50, 126.42, 124.04, 121.32, 119.44, 116.09, 114.74, 113.42, 111.67, 109.00, 104.64, 55.86, 54.91, 48.69, 37.92, 31.36, 27.27, 27.07, 22.67, 18.08. HRMS (ESI+) calcd. for [$C_{45}H_{50}N_3O_5S^+$] 744.3466, found 744.3467.

2-((1*E*,3*Z*,5*E*)-4-(1-Hydroxyethyl)-7-((*E*)-5-methoxy-1,3,3-trimethylindolin-2-ylidene)hepta-1,3,5-trien-1-yl)-5-methoxy-1,3,3-trimethyl-3*H*-indol-1-ium iodide (10)

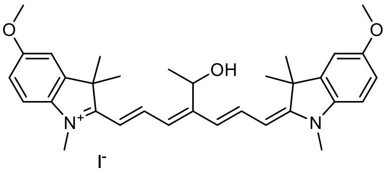

Compound obtained as a side product (scheme below), isolated in a small amount from a reaction performed according to the general procedure from the corresponding Zincke salt (20 mg, 0.035 mmol, 1 eq) and **7a** (35 mg, 0.11 mmol, 3 eq). Column chromatography SiO_2 MeOH in CH_2Cl_2 from 2 to 5%. Yield: 2 mg (11%). Dark green solid. **Note:** Unfortunately, managed to isolate this side product only on a single occasion and its small amount precluded its further purification and characterization, and therefore only ^{1}H NMR, HRMS and UV-vis spectra are provided. ^{1}H NMR (400 MHz, *d*$_4$-MeOH) δ (ppm) 8.32 (t, *J* = 13.5 Hz, 2H), 7.18 (d, *J* = 8.7 Hz, 2H), 7.10 (d, *J* = 2.5 Hz, 2H), 6.96 (dd, *J* = 8.7, 2.5 Hz, 2H), 6.52 (d, *J* = 13.4 Hz, 2H), 6.21 (d, *J* = 13.5 Hz, 2H), 5.18 (q, *J* = 6.8 Hz, 1H), 3.84 (s, 6H), 3.58 (s, 6H), 1.69 (d, J = 2.8 Hz, 12H), 1.55 (d, *J* = 6.6 Hz, 3H). HRMS (ESI+) calcd. for [$C_{33}H_{41}N_2O_3^+$] 513.3112, found 513.3106.

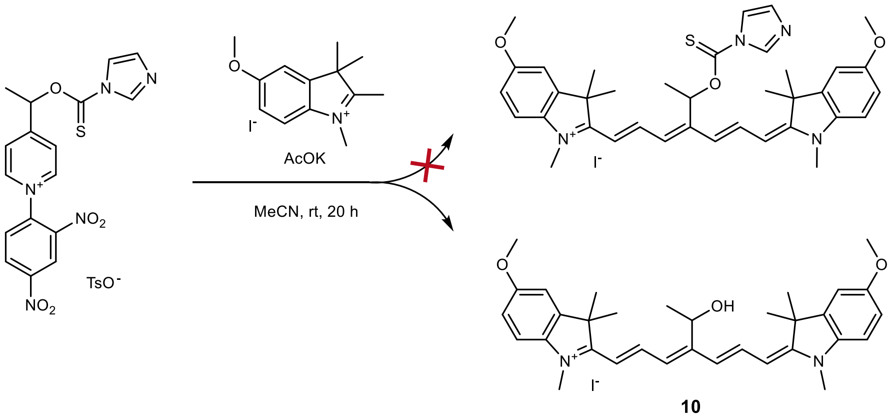

Scheme S1. Synthetic scheme of cyanine **10**.

Photophysical and Photochemical Measurements

Fluorescence Measurements and Quantum Yields

Emission spectra were measured in HEPES (pH 7.4, 20 mM with 10% DMF), MeOH or DMF using a fluorescence spectrometer in a 1.0 cm quartz fluorescence cuvette at 20°C. The sample concentrations were adjusted to keep the absorbance <0.15 at the corresponding excitation wavelength. Each sample was measured five times, and the spectra were averaged. Emission spectra were normalized and corrected by the photomultiplier sensitivity function using correction files supplied by the manufacturer. The fluorescence quantum yields(Φ_F) were determined using integration sphere, each sample was measured five times using independent solutions keeping A<0.15, and the values were averaged.

Photolysis and Dark Stability

A solution of photocage **1a–b** (*c* ~2 × 10^{-5} M, 3000 µL, A < 1.5) in aerated HEPES (pH 7.4, 20 mM with 10% DMF) was stirred and left to equilibrate for 2–3 min at 20°C. Afterward, the sample was irradiated with LEDs at 820 nm (~50 mW/cm^2) and the progress of the irradiation was monitored at the given time intervals by UV−vis spectrometry using a diode-array

spectrophotometer. The total irradiation time was selected to reach >95% conversion and to obtain minimum of 15 experimental points. The procedure was repeated three times. The dark stability of **1a–b** was recorded using the same procedure with exclusion of the irradiation source.

NMR Irradiation Experiments

Photocage **1a–b** (~0.5–1 mg) was dissolved in aerated or degassed methanol (0.5 mL). Methanol for experiments in oxygen-free conditions was extensively degassed by 3–4 cycles of sonication under vacuum (~3 min each) followed by 3–4 cycles of sonication under N_2 overpressure (~3 min each) in acetone-dry ice bath and the samples were prepared in a glovebox. The NMR tubes were then irradiated with LEDs at 810 nm (~300 mW/cm^2) at a distance of ~3 cm, with active cooling by a fan and ^{1}H NMR spectra were recorded after indicated time intervals. For the experiment with compound **1a**, the NMR sample after irradiation with exclusion of oxygen was diluted and measured using HRMS.

Determination of Chemical Yield of H_2S Release with Methylene Blue Assay

Cyanine **1a**: A solution of cyanine **1a** (1 mL, c = 150 × 10^{-6} M) in HEPES buffer (pH 7.4, 20 mM) with 1% MeOH was placed in a 1.0 cm quartz PTFE screw-cap cuvette equipped with a stirring bar. A solution of CA (from bovine erythrocytes, 3500 U/mg, 1 mg/mL, 150 μL) was added to the sample solution. Zinc acetate (300 μL, 1% w/v) added to prevent oxidation of the H_2S formed and precipitate H_2S as ZnS, respectively. The solution was stirred and irradiated with LED array at 820 nm (~300 mW/cm^2) for 1 h then *N*,*N*-dimethyl-*p*-phenylene diamine sulfate (200 μL, 20 mM in 7.2 M aq. HCl), $FeCl_3$ (200 μL, 30 mM in 1.2 M aq. HCl) and water (1.45 mL) were added. The samples were incubated at room temperature for 1 h, the absorbance at 670 nm was recorded and used for H_2S concentration calculation using a calibration curve for NaSH (Figure S33).

Cyanine **1b**: A solution of cyanine **1b** (1 mL, c = 50 × 10^{-6} M) in HEPES buffer (pH 7.4, 20 mM) with 5% MeCN was placed in a 1.0 cm quartz PTFE screw-cap cuvette equipped with a stirring bar. A solution of CA (from bovine erythrocytes, 3500 U/mg, 1 mg/mL, 150 μL) was added to the sample solution. Zinc acetate (300 μL, 1% w/v) added to prevent oxidation of the H_2S formed and precipitate H_2S as ZnS, respectively. The solution was stirred and irradiated with a LED array at 820 nm (300 mW/cm^2) for 3 h then *N*,*N*-dimethyl-*p*-phenylene diamine sulfate (200 μL, 20 mM in 7.2 M aq. HCl), $FeCl_3$ (200 μL, 30 mM in 1.2 M aq. HCl) were added. Samples were incubated at room temperature for 1 h and water (1.15 mL) was added. Samples were filtered through a syringe filter, absorbance at 670 nm was recorded and used for H_2S concentration calculation using a calibration curve for NaSH (Figure S34)

Determination of the Yield of Coumarin Release by Emission Spectroscopy.

A solution of **1a–b** (1.6-18 × 1.0^{-6} M, 3 mL) in HEPES (pH = 7.4, 20 mM, with 10% DMF) adjusted to A_{812} and A_{805} <0.15 respectively in a matched 1.0 cm quartz was stirred and irradiated with LED array at 820 nm (~20 mW cm^{-2}). The progress of the photolysis was monitored periodically at the given time intervals simultaneously by emission spectroscopy (λ_{exc} = 365 nm). Each spectrum was recorded once to minimize exposure of the sample to the excitation light source. The emission spectra were recorded and used for calculation of the yield of cargo release using a calibration curve for coumarin cargo (Figure S30–32). Emission spectra were smotthed (Method: Savitzky-Golay; Points of Window: 10, Boundary condition: None; Polynominal order: 2).

Methodology of Biological experiments

Cell Viability Assays.
HeLa cells were seeded in 96 well plates at a density of 4×10^3 cells per well for 72 h viability assay and 8×10^3 cells per well for 24 h viability assay and grown to 80% confluency for 24 or 72 h 37°C at 5% CO_2 atmosphere. Media was removed and substituted with media containing different concentrations of **1a** and **1c** their photoproducts of **1a** and **1c** or cargo **2** (DMSO stock solution with c ~ 1 × 10^{-2} M of parent compound was diluted with DMEM (4.5g/L glucose, L-glutamine, 1% pyruvate, 10% FBS, 1% P/S) to obtain stock solution with 1% DMSO, which was further diluted with DMEM (4.5 g glucose, L-glutamine, 1% pyruvate, 10% FBS, 1% P/S) to reach the final concentration of compound). The amount of DMSO in well was kept stable at 0.1%. The photoproducts were prepared by irradiation of solution of **1a** and **1c** (c ~ 1 × 10^{-2} M) in DMSO at 810 nm light (50 mW/cm^2) for 20 hours. The cells were incubated for 24 h or 72 h at 37°C at 5% CO_2 atmosphere after the addition of compound. The cells were then left to equilibrate to room temperature for 10 minutes and a half of the media (50 µL) media was removed. 50 µL of CellTitre-Glo (prepared according to Promega protocol) was added and the cells were mixed with the reagent using rocking shaker at maximum speed for 15 minutes. The luminescence was detected using plate reader in luminescence mode as an integral over all wavelengths (1000 ms integration time), and a plot of the cell viability was obtained from three replicates under each condition. All experiments were repeated three times using cells from different passages. The cell viability was calculated using cells with 0 µM concentration of compound as a refence.

Widefield Fluorescence Microscopy Studies.
HeLa cells were seeded in 8 well microscopy slides (ibidi) at density of 2.2×10^4 cells per well and grown to 80% confluency for 24 hours at 37°C at 5% CO_2 atmosphere. Media was removed and substituted with DMEM (4.5 g/L glucose, L-glutamine, 1% pyruvate, 10% FBS, 1% P/S, w/o phenol red) containing commercial organelle-targeted dye MitoTracker Deep Red (c ~ 50 nM) and H_2S-responsive fluorescent probe Mito-HS[4] (c ~ 5 µM). The amount of DMSO in well was kept stable at 0.1%. After 1h of incubation at 37°C at 5% CO_2 atmosphere media was removed, wells were washed with DMEM (4.5 g/L glucose, L-glutamine, 1% pyruvate, 10% FBS, 1% P/S, w/o phenol red) and solution of **1a** (c ~ 2 or 4 µM) or **2** (c ~ 2 µM) in DMEM (4.5 g/L glucose, L-glutamine, 1% pyruvate, 10% FBS, 1% P/S, w/o phenol red) was added. The amount of DMSO in well was kept stable at 0.1%. After 30 min of incubation at 37°C at 5% CO_2 atmosphere media was removed and washing procedure was repeated 5 times to eliminate residues of the compounds used for incubation. A sample without MitoTracker Deep Red and Mito-HS, containing only the solution of **1a** served as a control.

The cells were placed in light microscope and kept at 37°C at 5% CO_2 atmosphere throughout the duration of experiments. The cells were visualized using Leica DMi8 fully motorized, inverted microscope with a fluorescence light source Leica LED8 at either 40× (for statistical calculations in Figure 3B–C) or at 63× (Figure 3A and Figure 4) magnification, using brighfield channel (40 ms), coumarin fluorescence channel (exc. 390 nm, 18% intensity, exposure 150 ms, 420–450nm detection), Mito-HS channel (exc. 440 nm, 7% intensity, exposure 100 ms, 462–484 nm detection), MitoTracker Deep Red channel (exc. 635 nm, 3% intensity, exposure 100 ms, 666–724 nm detection) and NIR channel (exc. 747 nm, 10% intensity, exposure 1 s, 770–850 nm detection) in 20 cycles. Procedure was repeated with three other excitation wavelengths: 475 nm (10% intensity, exposure 1 s, 506-532 nm detection), 555 nm (10% intensity, exposure 1 s, 581-607 nm detection) and 635 nm (10% intensity, exposure 1 s, 666-724 nm detection) in 20 cycles. The images were processed using ImageJ. The corrected total cell fluorescence

(CTCF) was calculated from the fluorescence intensity by subtraction of the background intensity in the vicinity of the cells. This was performed for all channels and each time point between 0 and 400 seconds. Each time point was calculated using 30–50 living cells for experiments with **1a** under irradiation or in the dark, and 20< cells for control experiment involving no incubation or incubation with free coumarin **2**, or only Mito-HS. The experiments were performed using 3 technical and biological replicates. The enhancement was calculated as a ratio of $CTCF_t$ in time (*t*) and the initial CTF_0 was plotted against time. Average of the enhancement and standard deviation of the mean are depicted.

The data were processed using ImageJ and Fiji. For the representative Figure 3 the following post-processing was applied. Deconvolution using DeconvolutionLab2 plugin[5] and a Richardson-Lucy protocol using a generated PSF function[6] was applied to the cyanine and coumarin channels. Additional background subtraction with a rolling ball radius of 50 pixels was applied for the coumarin channel. Mito-HS channel was used without post-processing. The experiments under irradiation and the control dark experiment within the respective channels were processed using an identical protocol and brightness/contrast settings. Background correction with a rolling ball radius of 50-100 pixels and no deconvolution was used in Figure 4. Pearson's correlation coefficients were calculated using Coloc2 plugin bundled with Fiji.

Scheme S2. Activation of mito-HS probe by H_2S.[4]

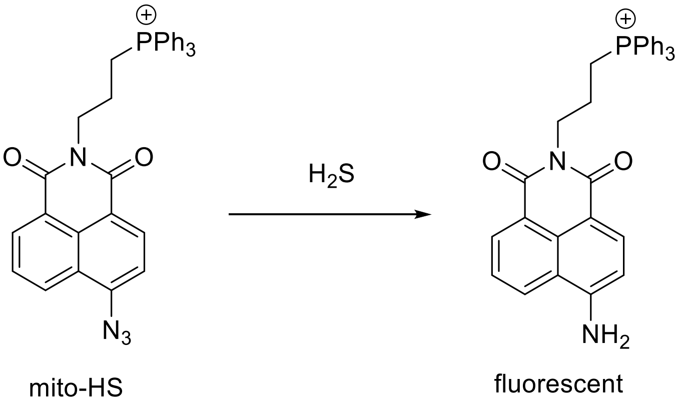

Partition Coefficient

To a vial containing 1-octanol (475 µL) and water (475 µL) a solution (50 µL) of photocage in MeOH was added. The mixture was shaken and then left to stand to let the fractions separate. 150 µL of the octanol fraction was collected and diluted with MeOH (2.85 mL) and the absorption of the sample at the maximum was measured. The same procedure was repeated for water fraction.

Photocage	*P*	log *P*
**1a**	7.95	0.90
**1b**	45.12	1.65
**1c**	1.15	0.06

NMR spectroscopy

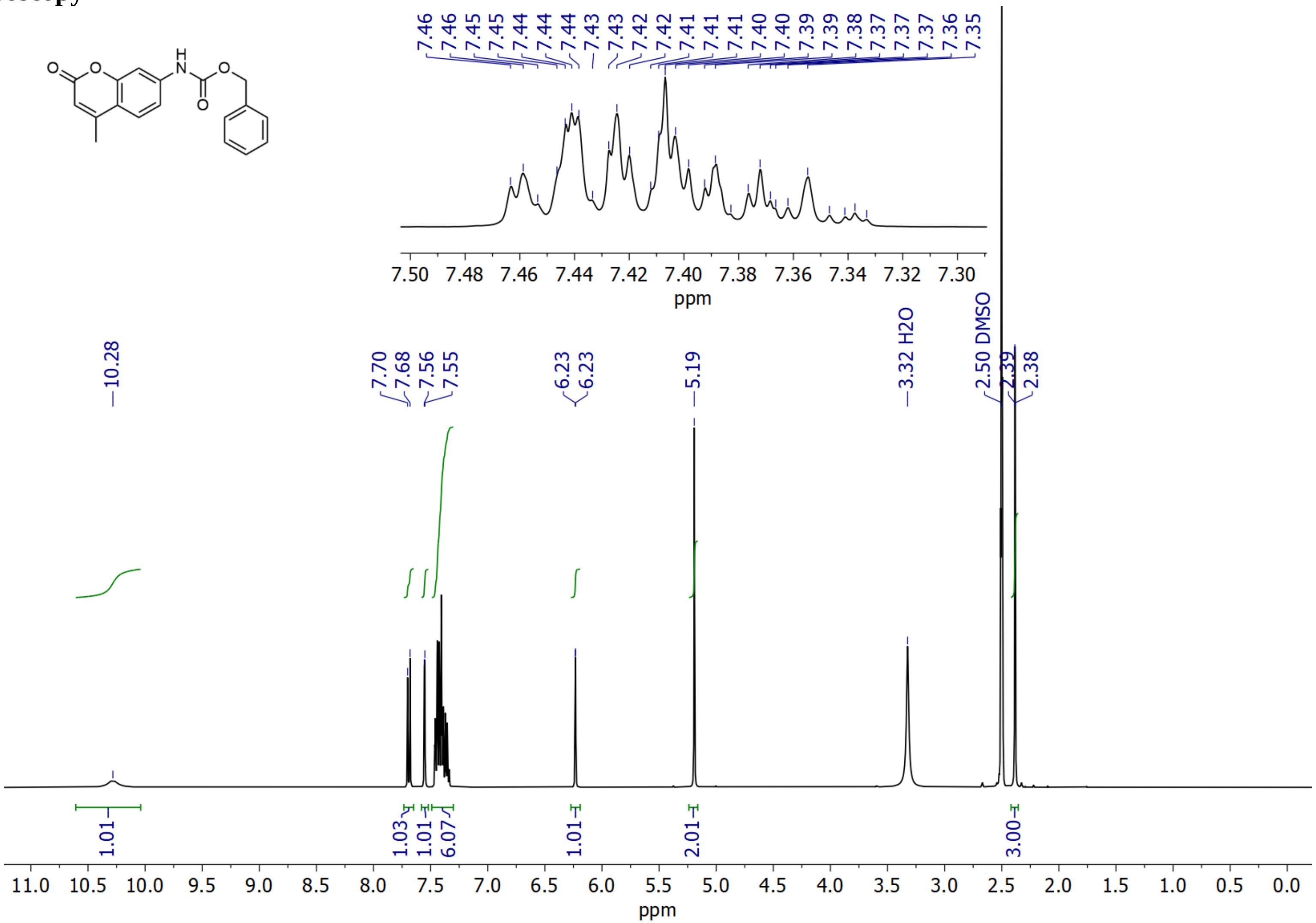

Figure S1. ^{1}H NMR (400 MHz, d_6-DMSO): **9**

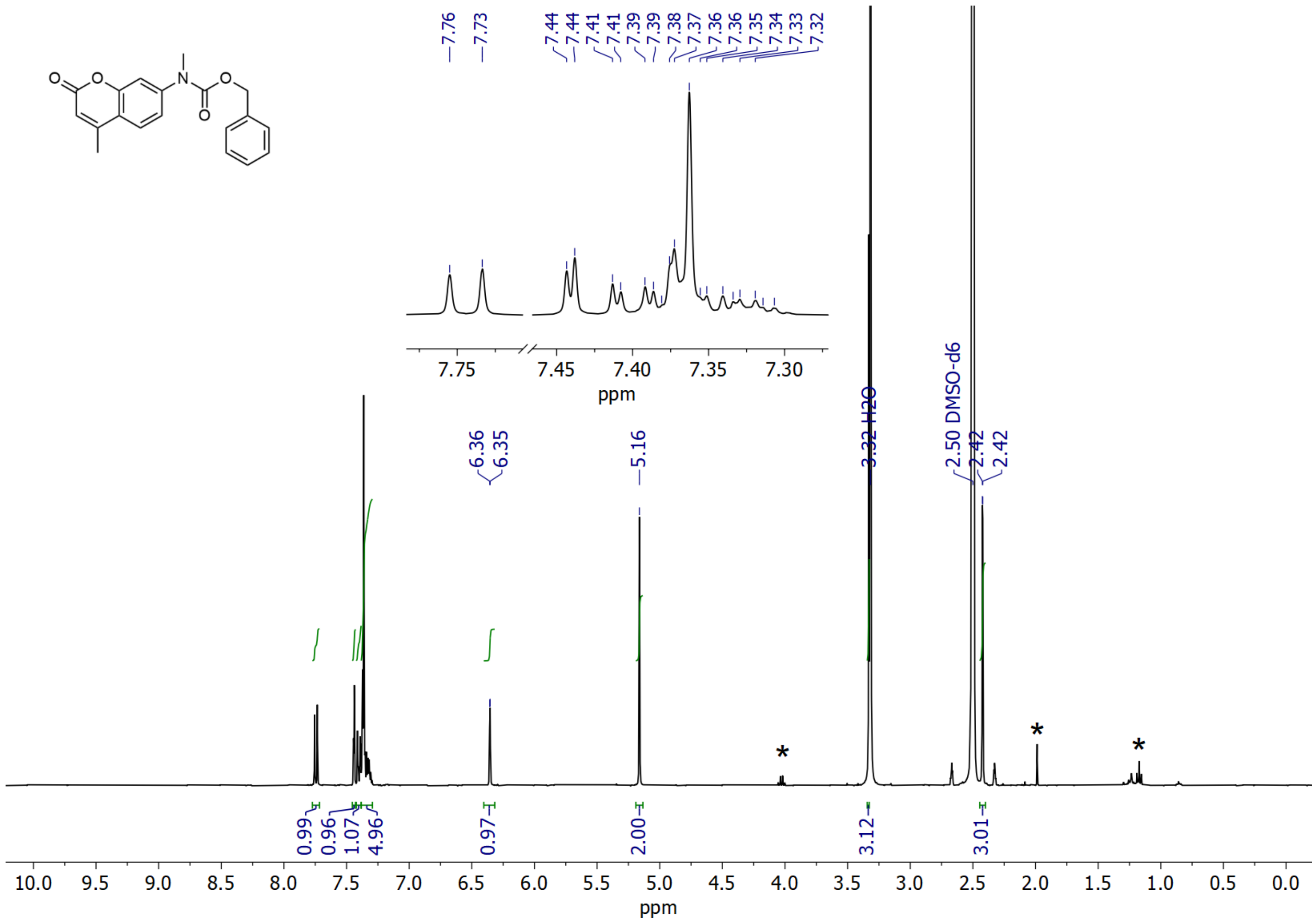

Figure S2. ^{1}H NMR (400 MHz, d_6-DMSO): **10**, ***EtOAc δ (ppm) 4.03 (q) 1.99 (s), 1.17 (t).

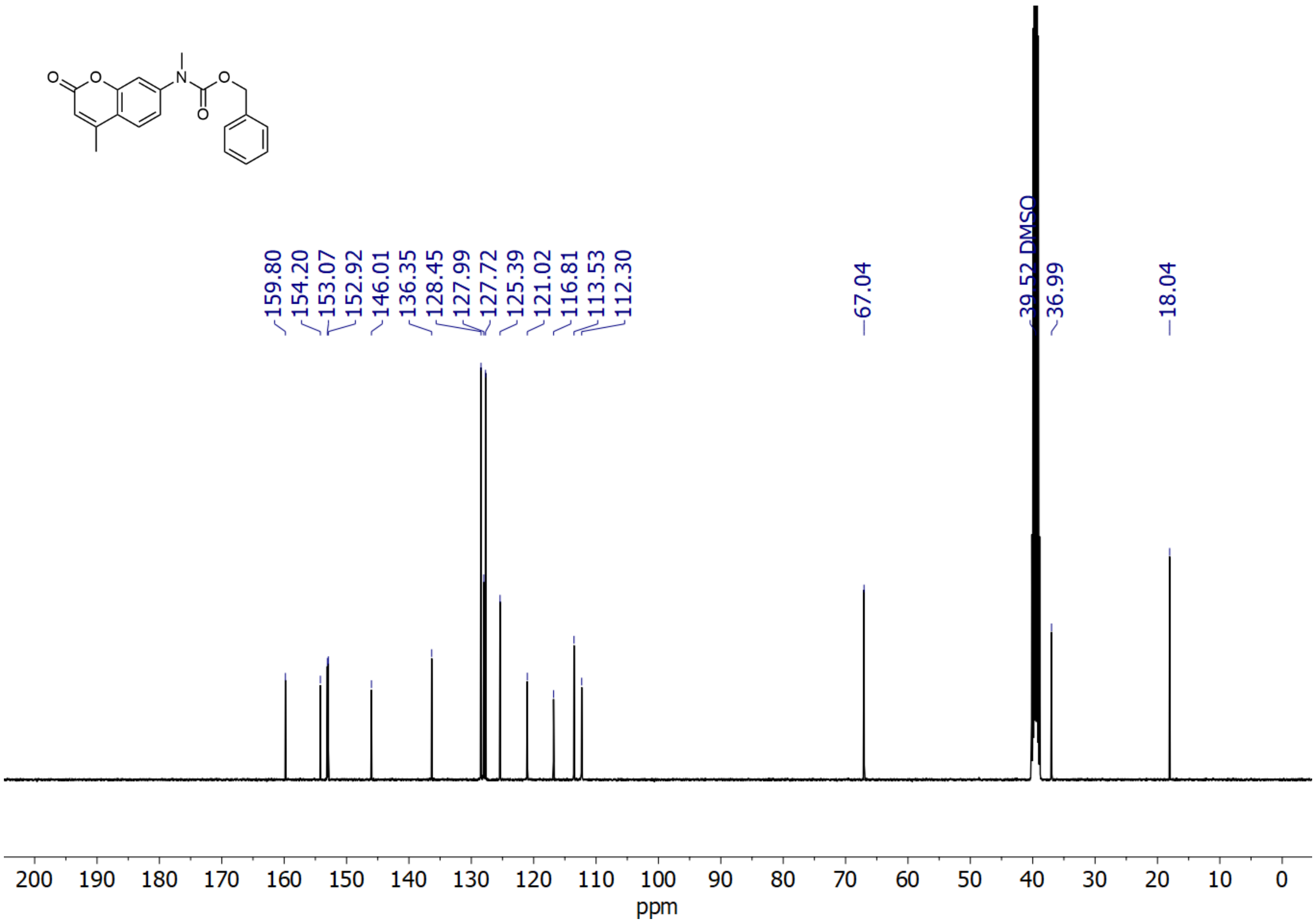

Figure S3. ^{13}C NMR (125 MHz, d_6-DMSO): **10**

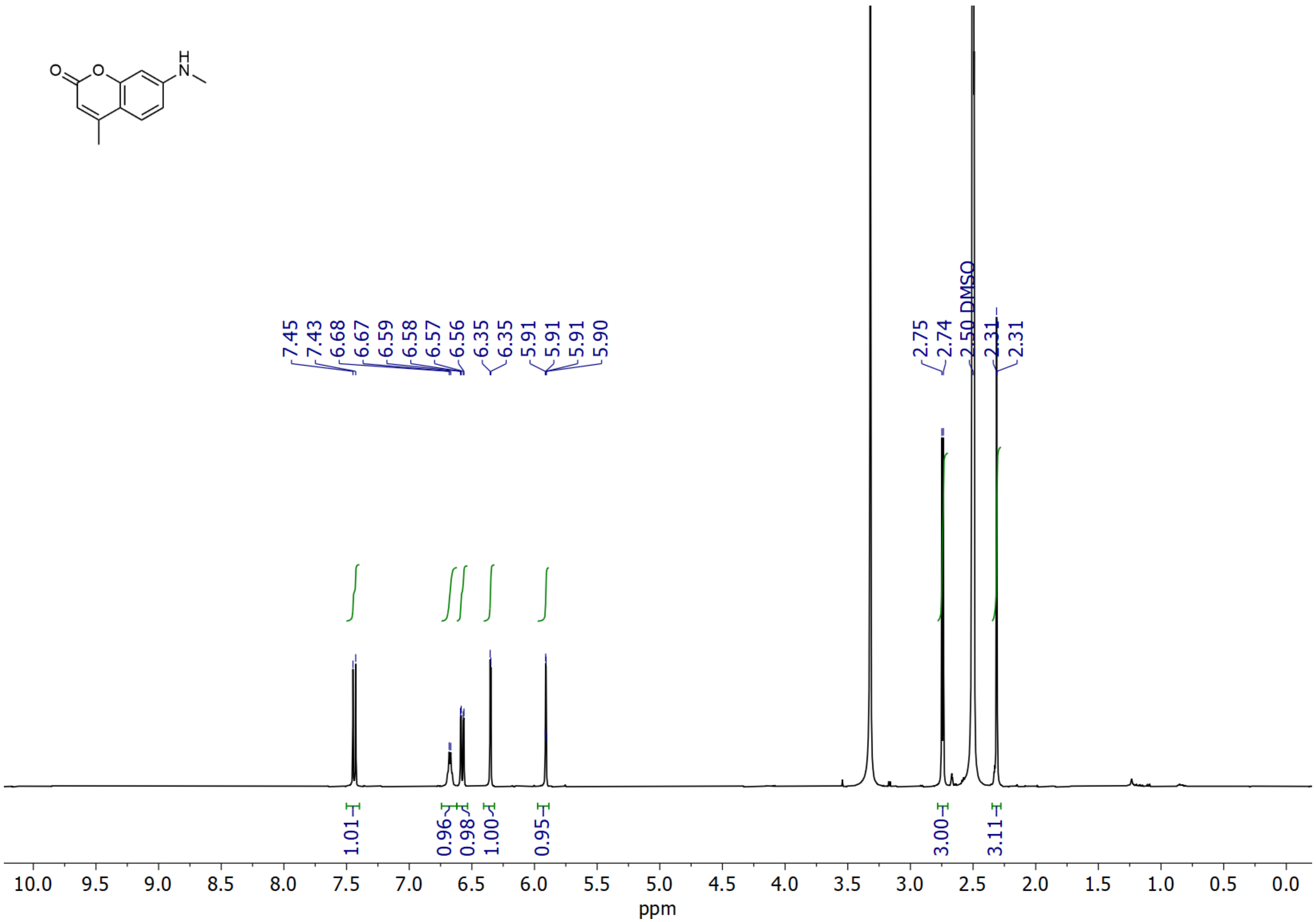

Figure S4. ^{1}H NMR (400 MHz, d_6-DMSO): **2**

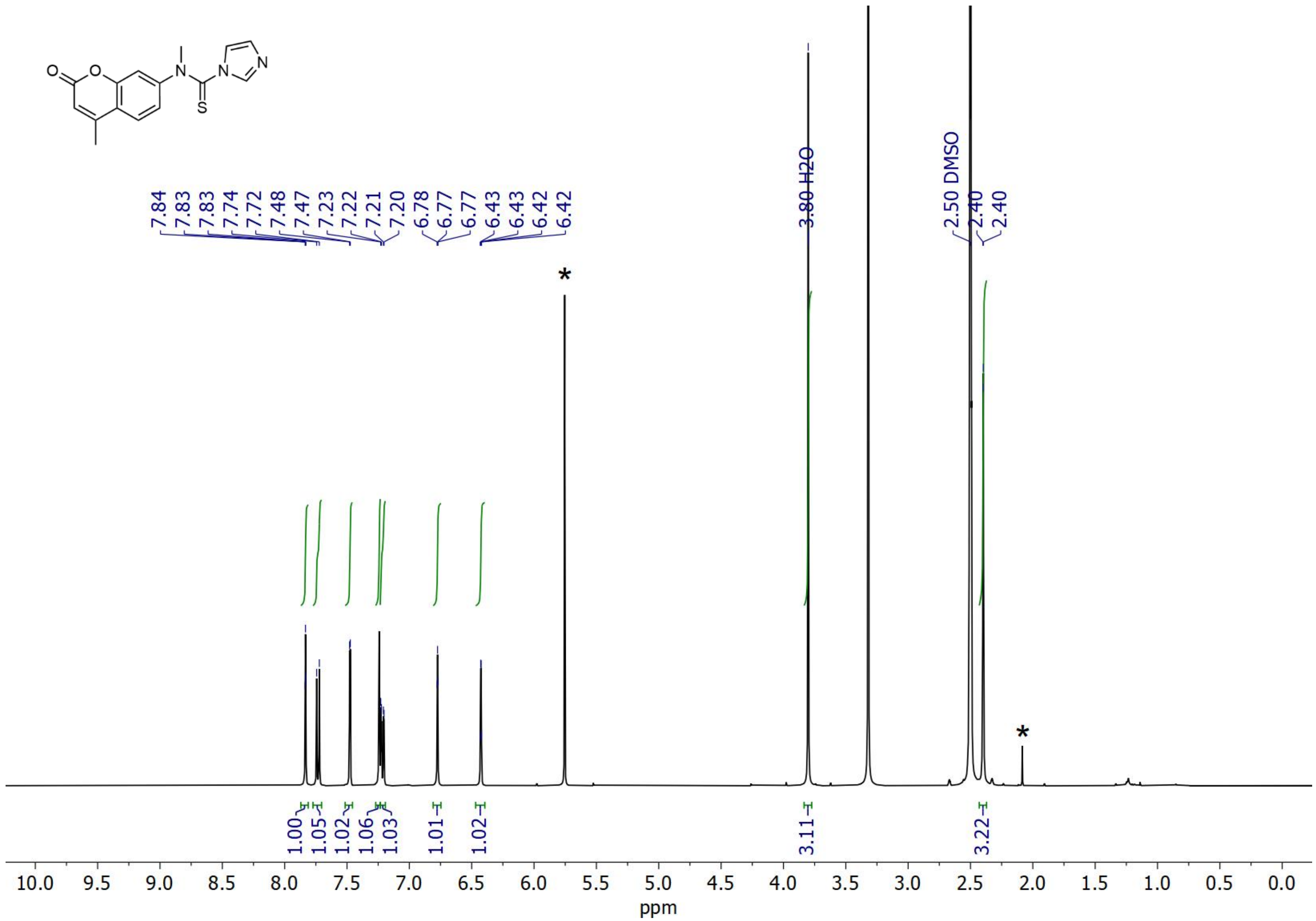

Figure S5. ^{1}H NMR (400 MHz, d_6-DMSO): **3a**, * CH_2Cl_2 δ (ppm) 5.75 (s), Me_2CO δ (ppm) 2.08 (s).

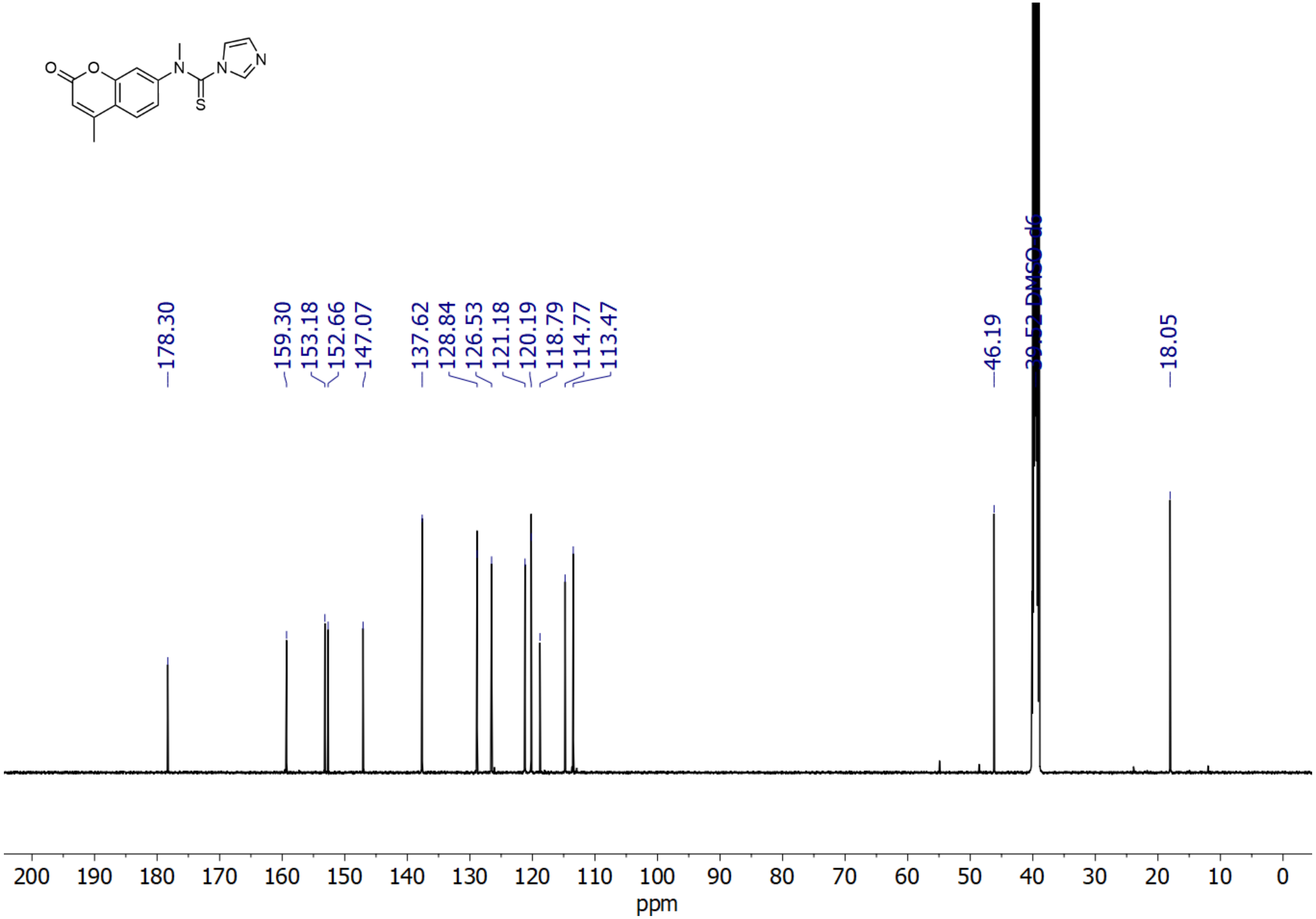

Figure S6. ^{13}C NMR (125 MHz, d_6-DMSO): **3a**

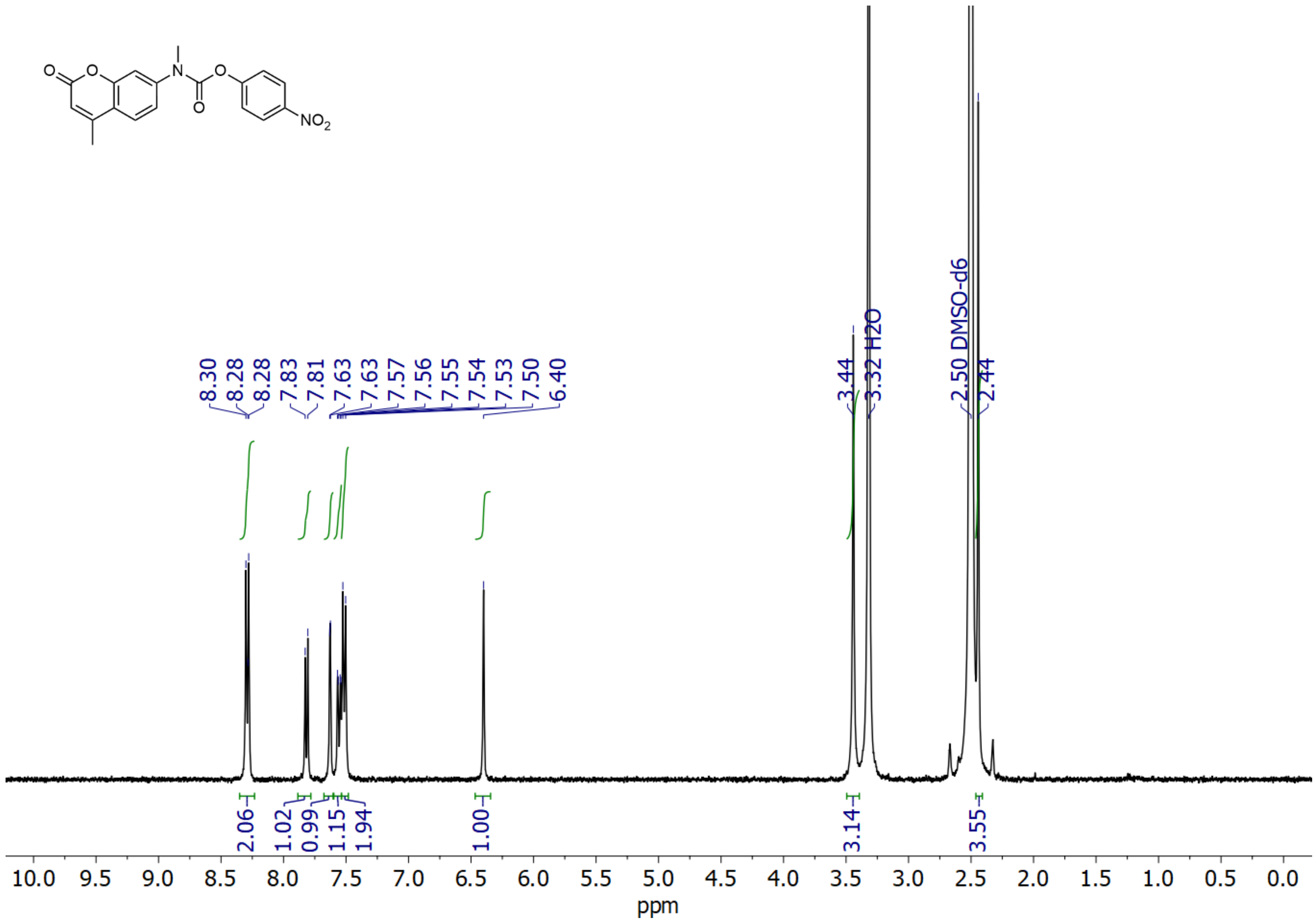

Figure S7. ^{1}H NMR (125 MHz, d_6-DMSO): **3b**

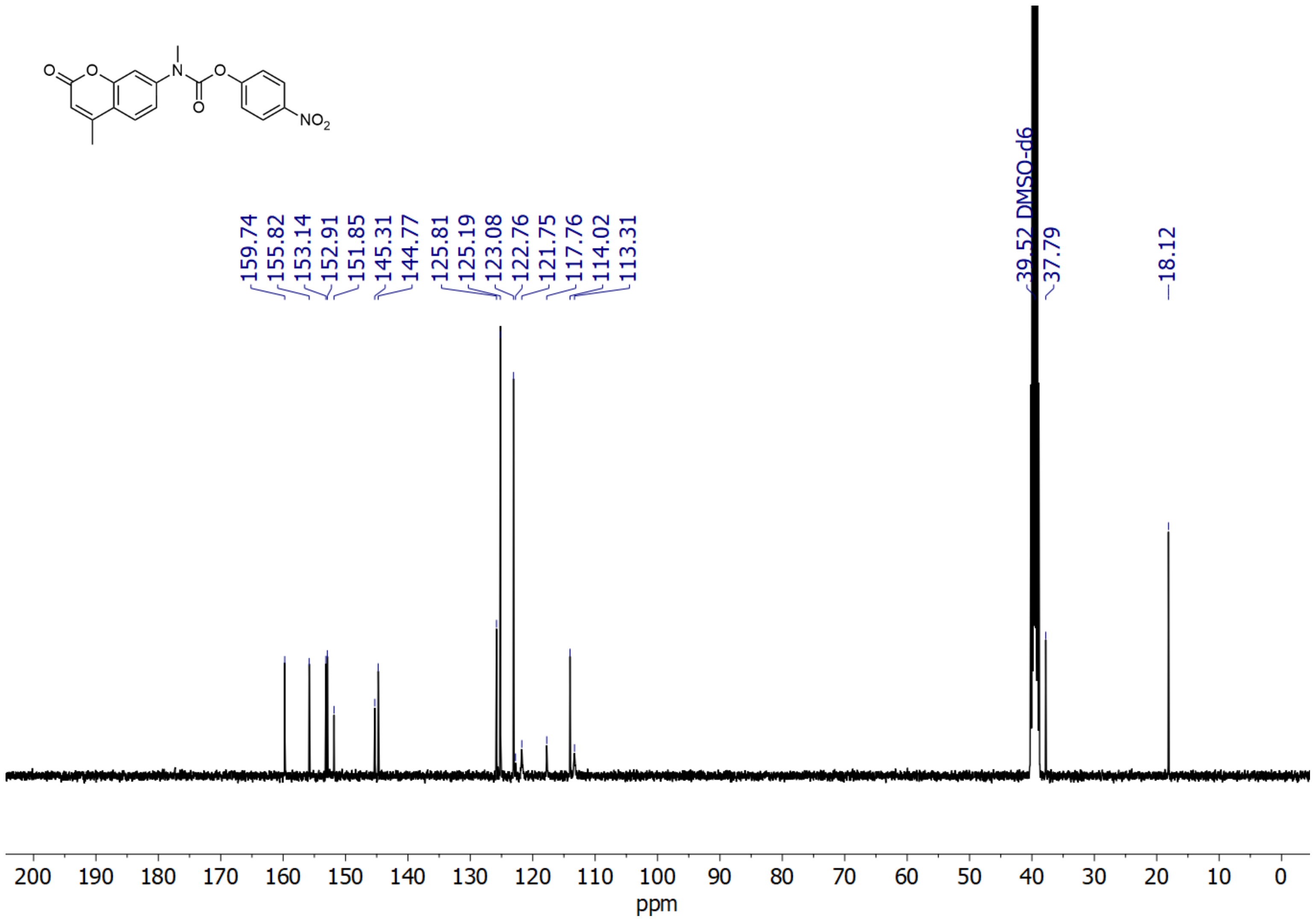

Figure S8. ^{13}C NMR (125 MHz, d_6-DMSO): **3b**

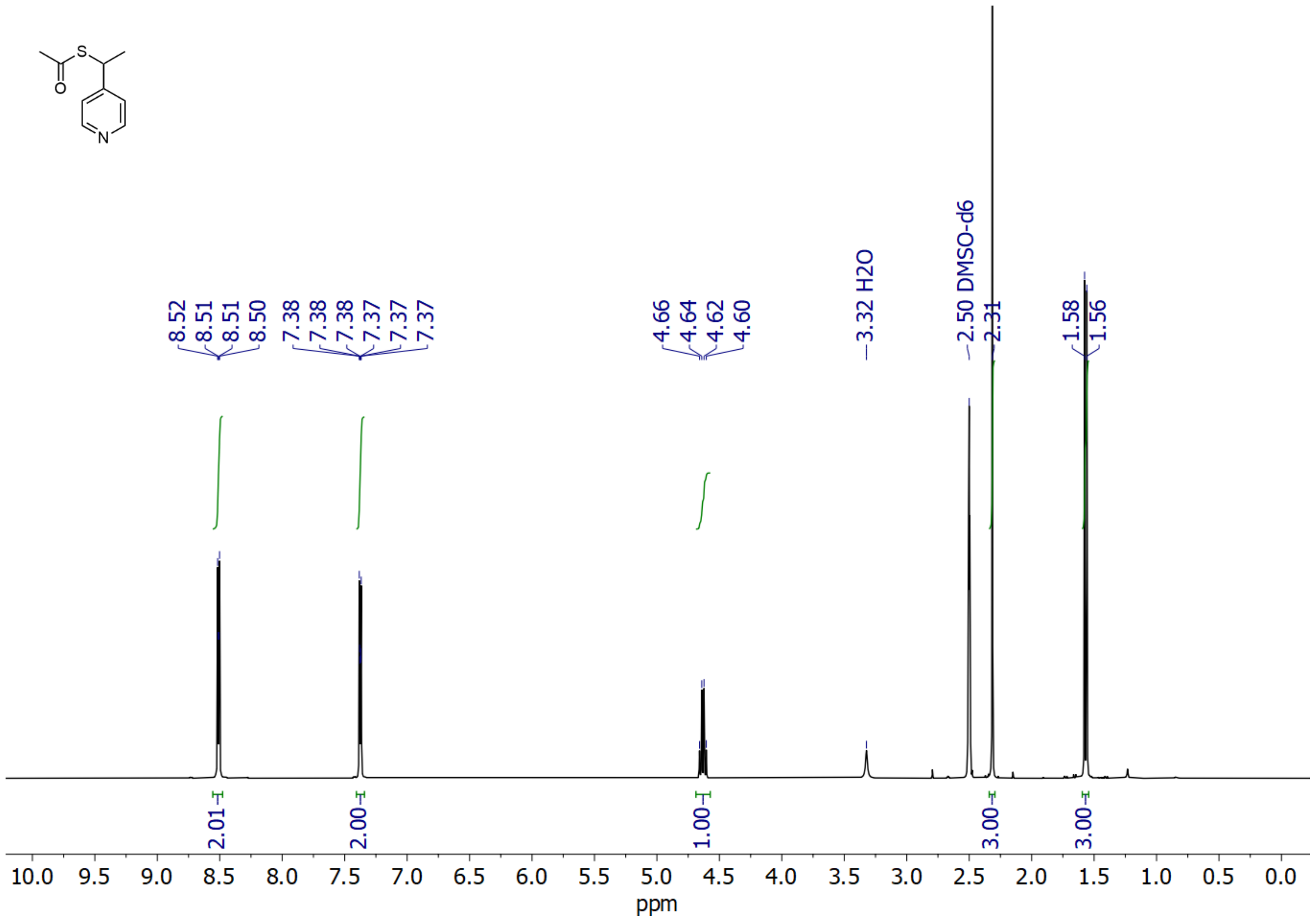

Figure S9. ^{1}H NMR (400 MHz, d_6-DMSO): **4b**

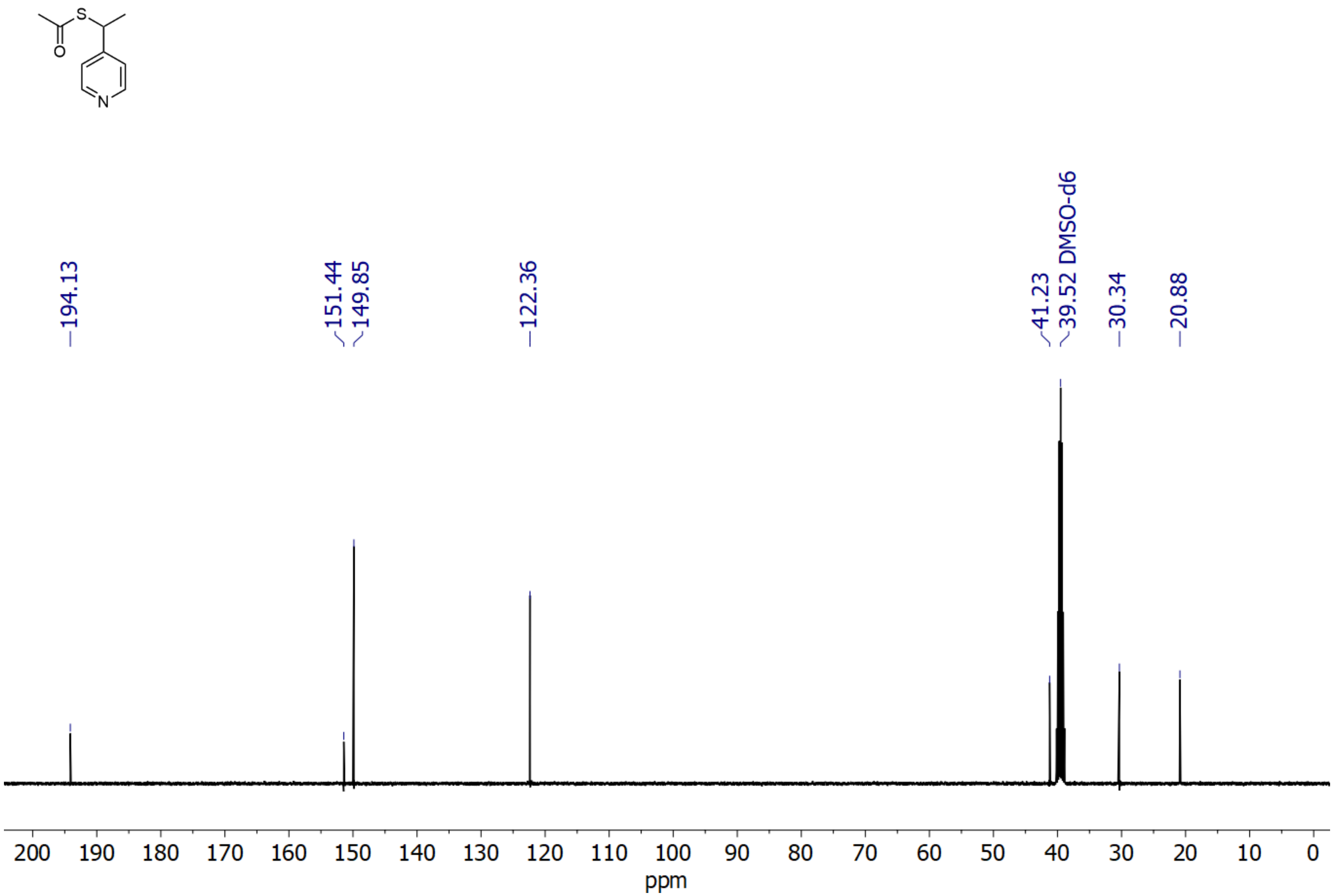

Figure S10. ^{13}C NMR (125 MHz, d_6-DMSO): **4b**

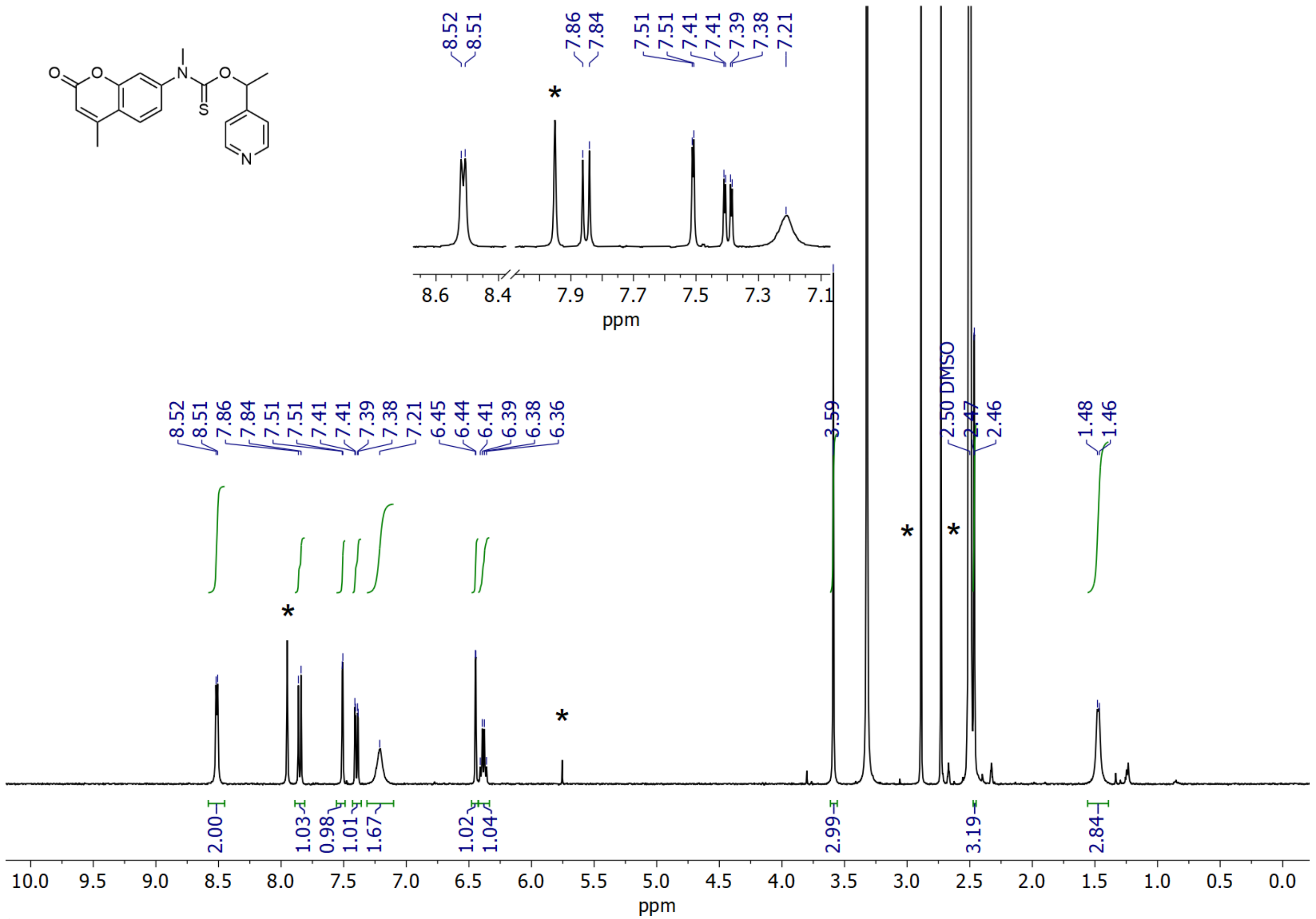

Figure S11. ^{1}H NMR (400 MHz, *d*$_6$-DMSO): **5a**, *DMF δ (ppm) 7.95 (s), 2.89 (s), 2.73 (s), CH_2Cl_2 δ (ppm) 5.75 (s).

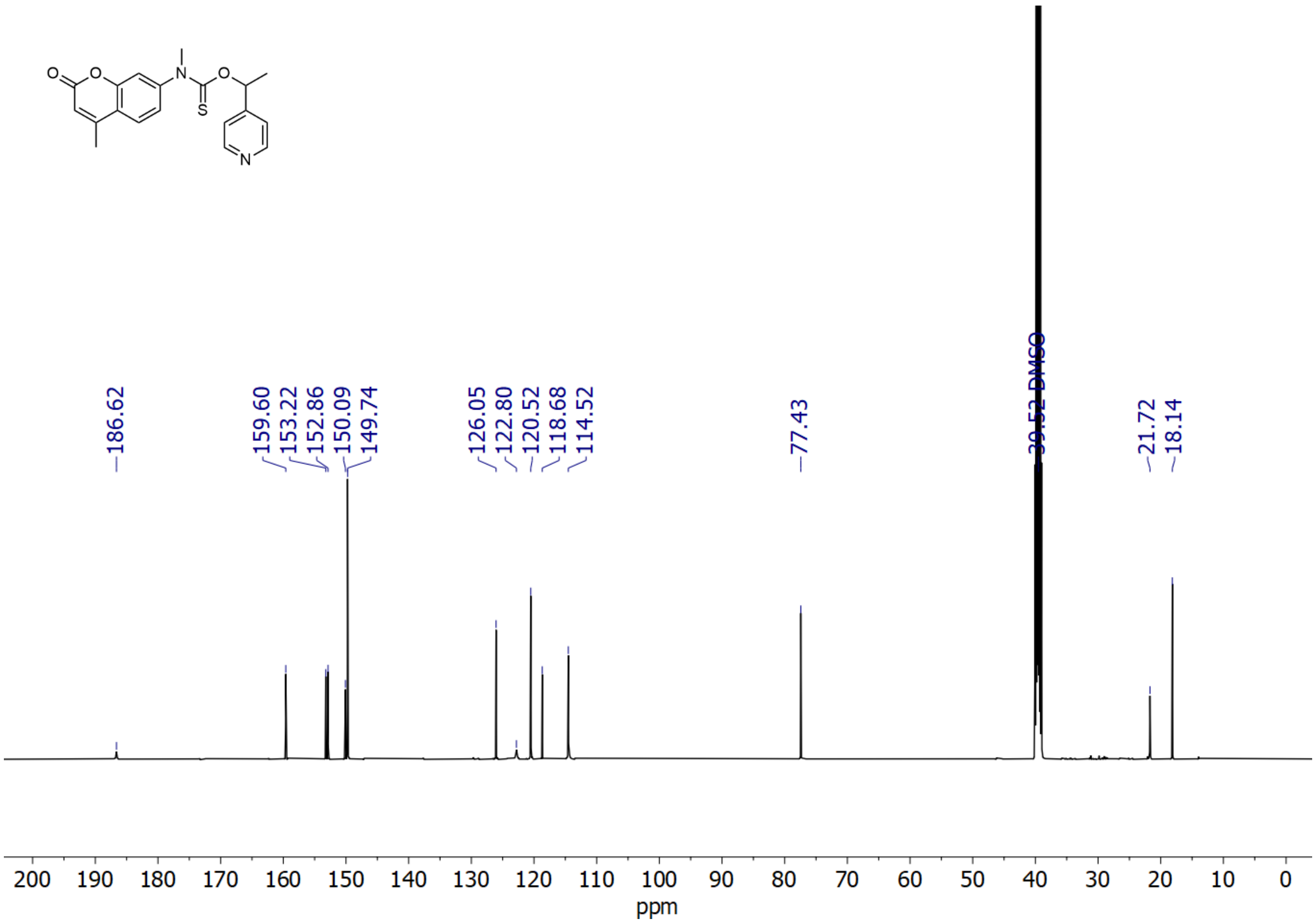

Figure S12. ^{13}C NMR (125 MHz, d_6-DMSO): **5a**

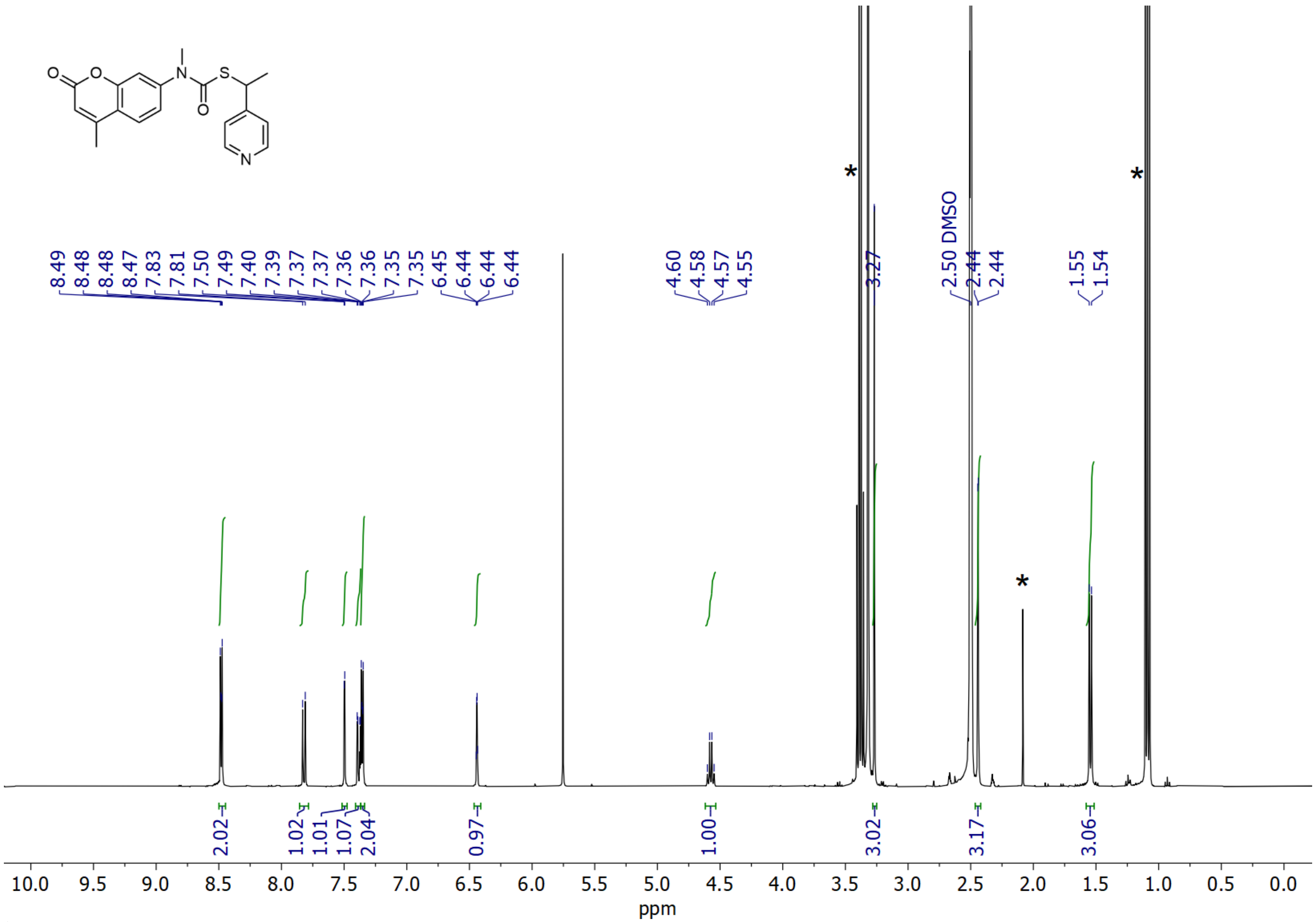

Figure S13. ^{1}H NMR (400 MHz, *d*$_6$-DMSO): **5b**, *Et_2O δ (ppm) 3.37 (q), 1.09 (t), Me_2CO δ (ppm) 2.08 (s).

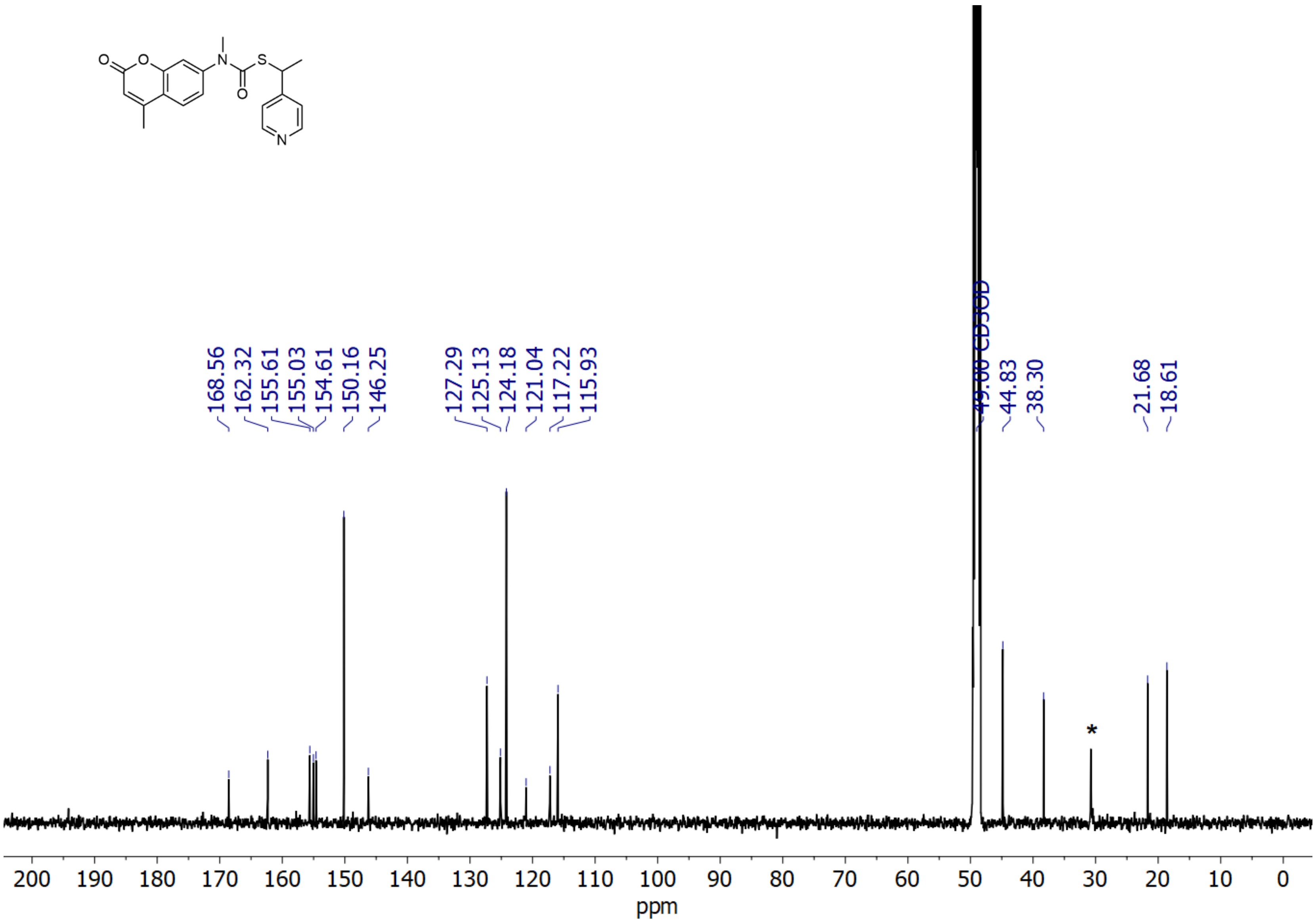

Figure S14. ^{13}C NMR (126 MHz, d_4-MeOH): **5b** *acetone residue at δ (ppm) 30.76.

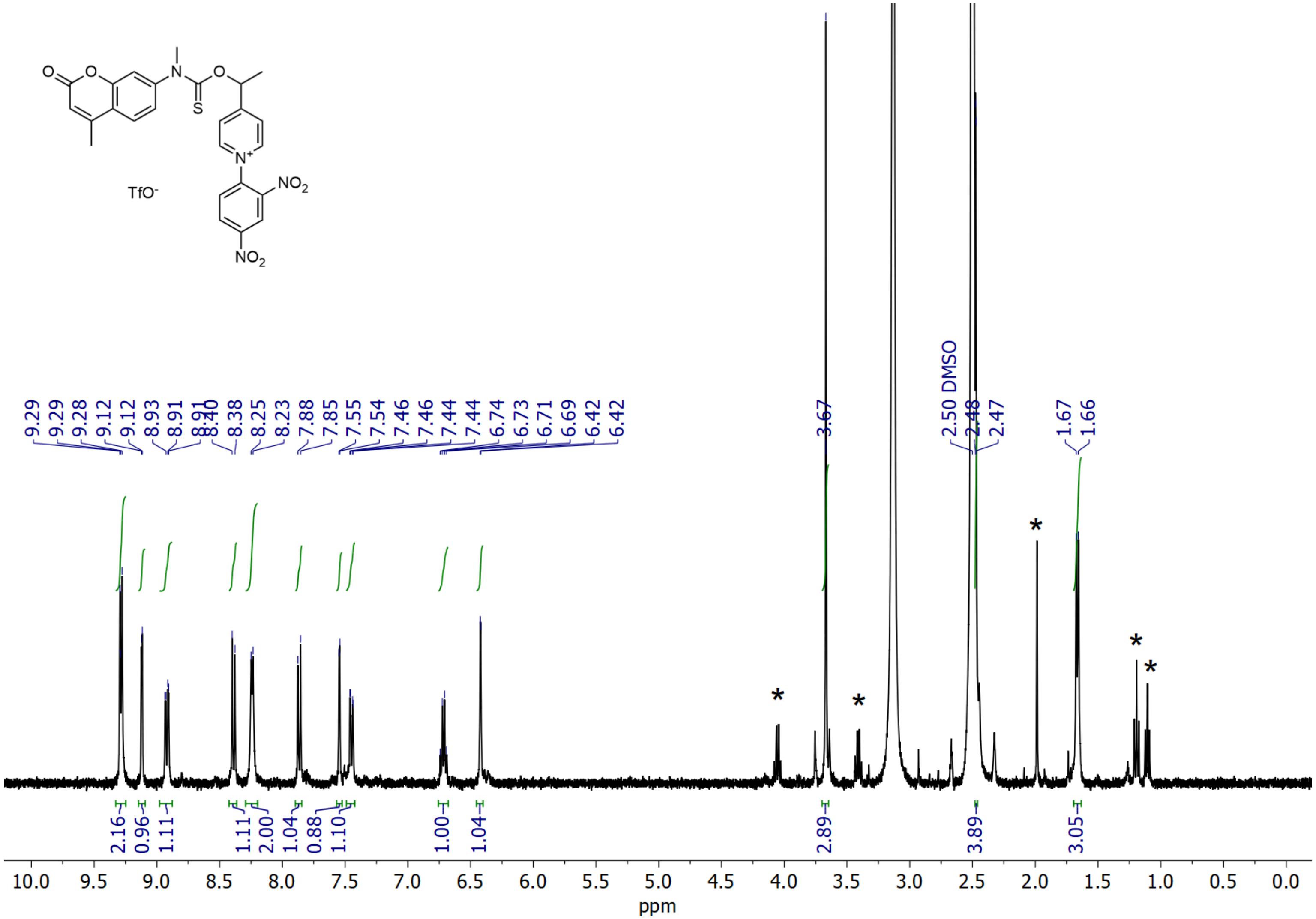

Figure S15. ^{1}H NMR (400 MHz, d_6-DMSO): **6a**, *Et_2O δ (ppm) 3.41 (q), 1.11 (t), EtOAc δ (ppm) 4.05 (q) 1.99 (s), 1.19 (t).

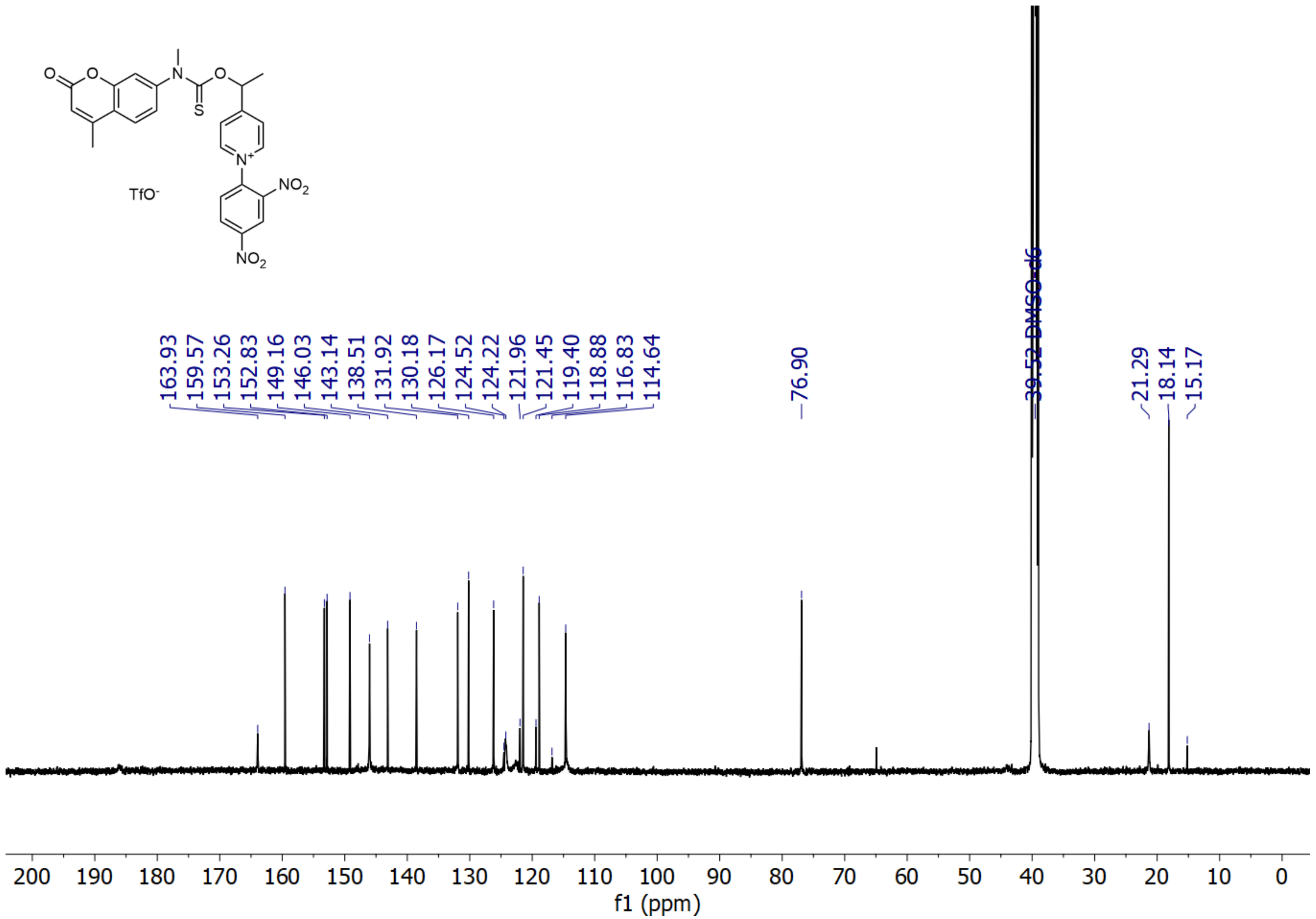

Figure S16. ^{13}C NMR (125 MHz, d_6-DMSO): **6a**

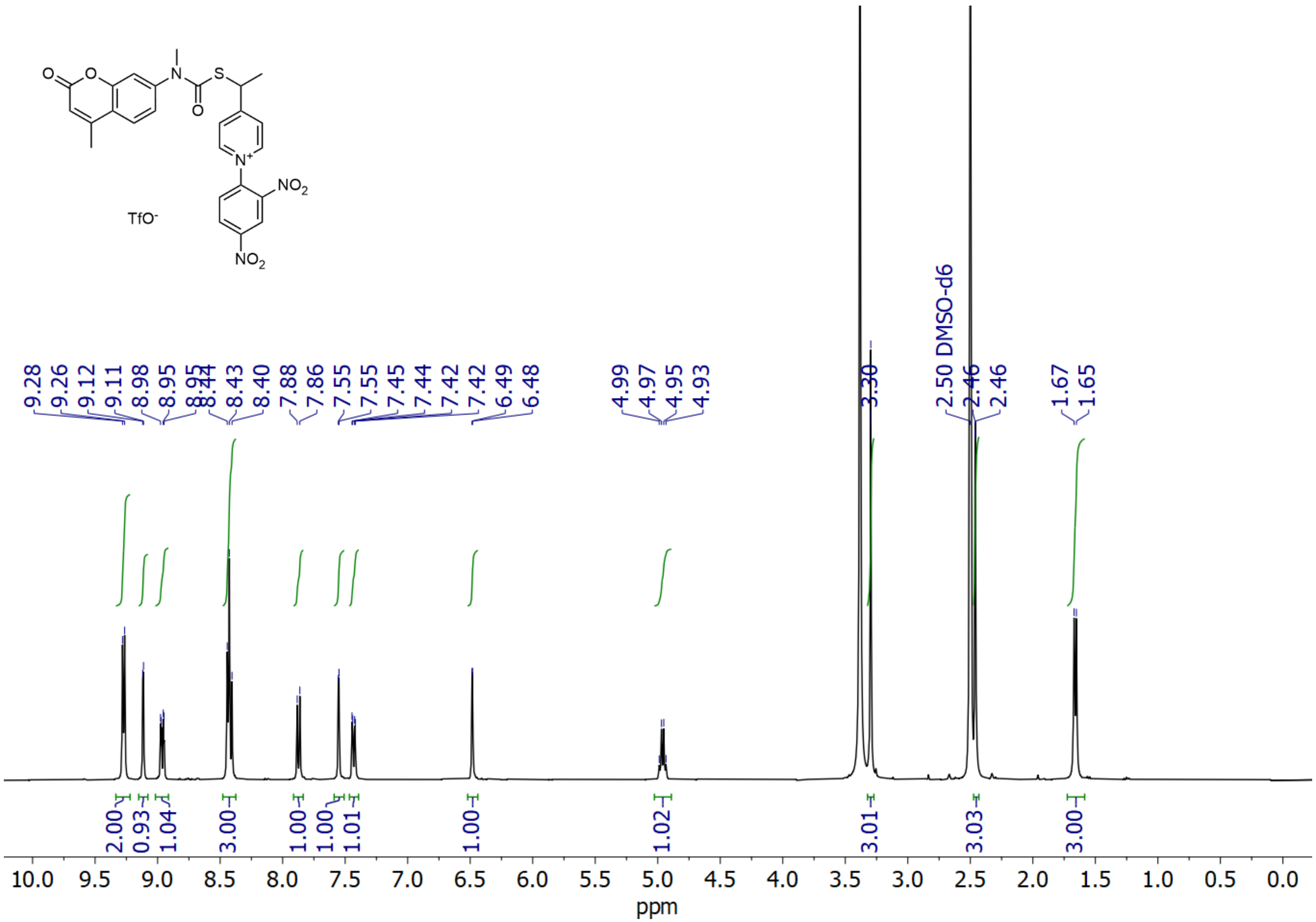

Figure S17. ^{1}H NMR (400 MHz, d_6-DMSO): **6b**

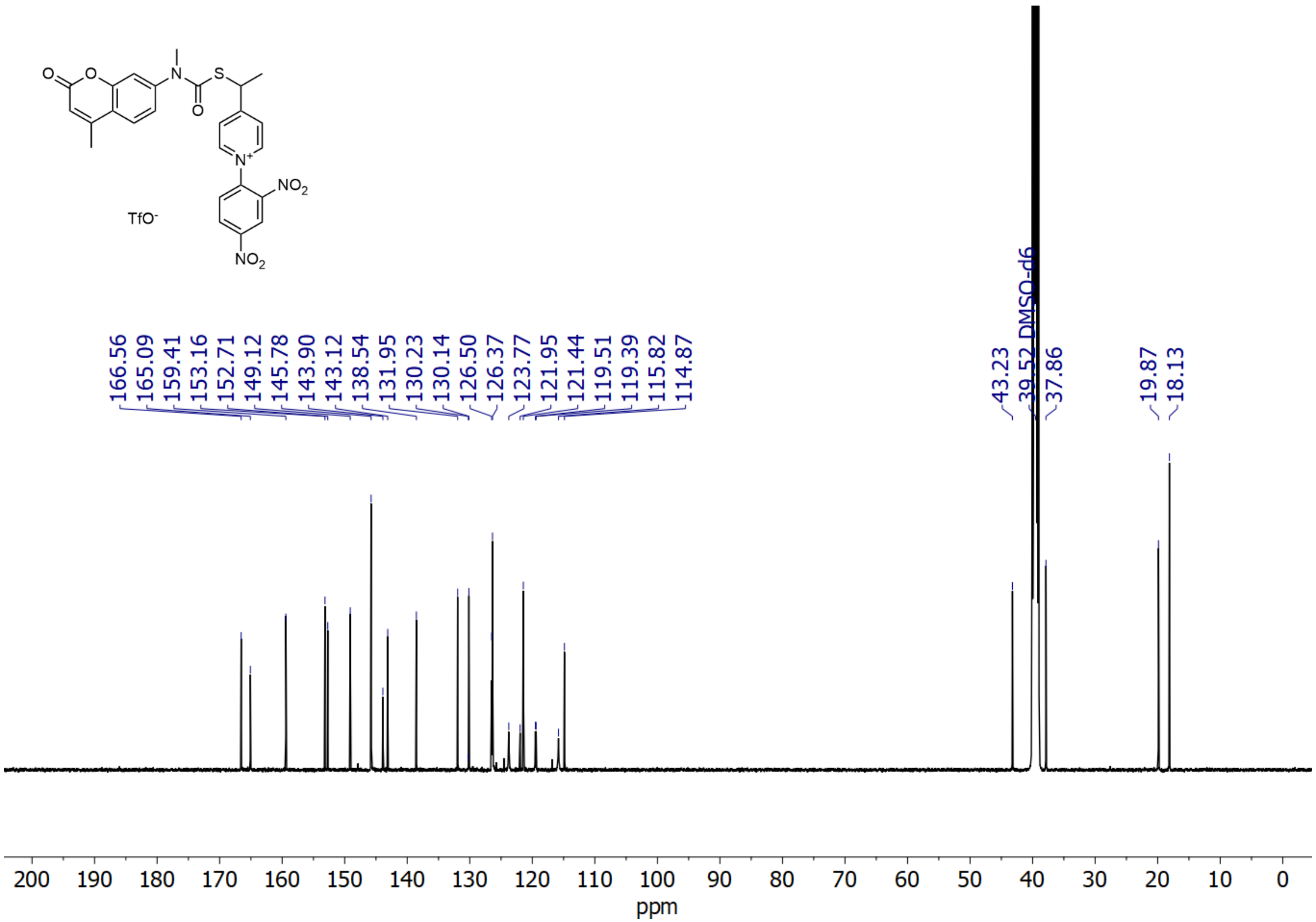

Figure S18. ^{13}C NMR (125 MHz, d_6-DMSO): **6b**

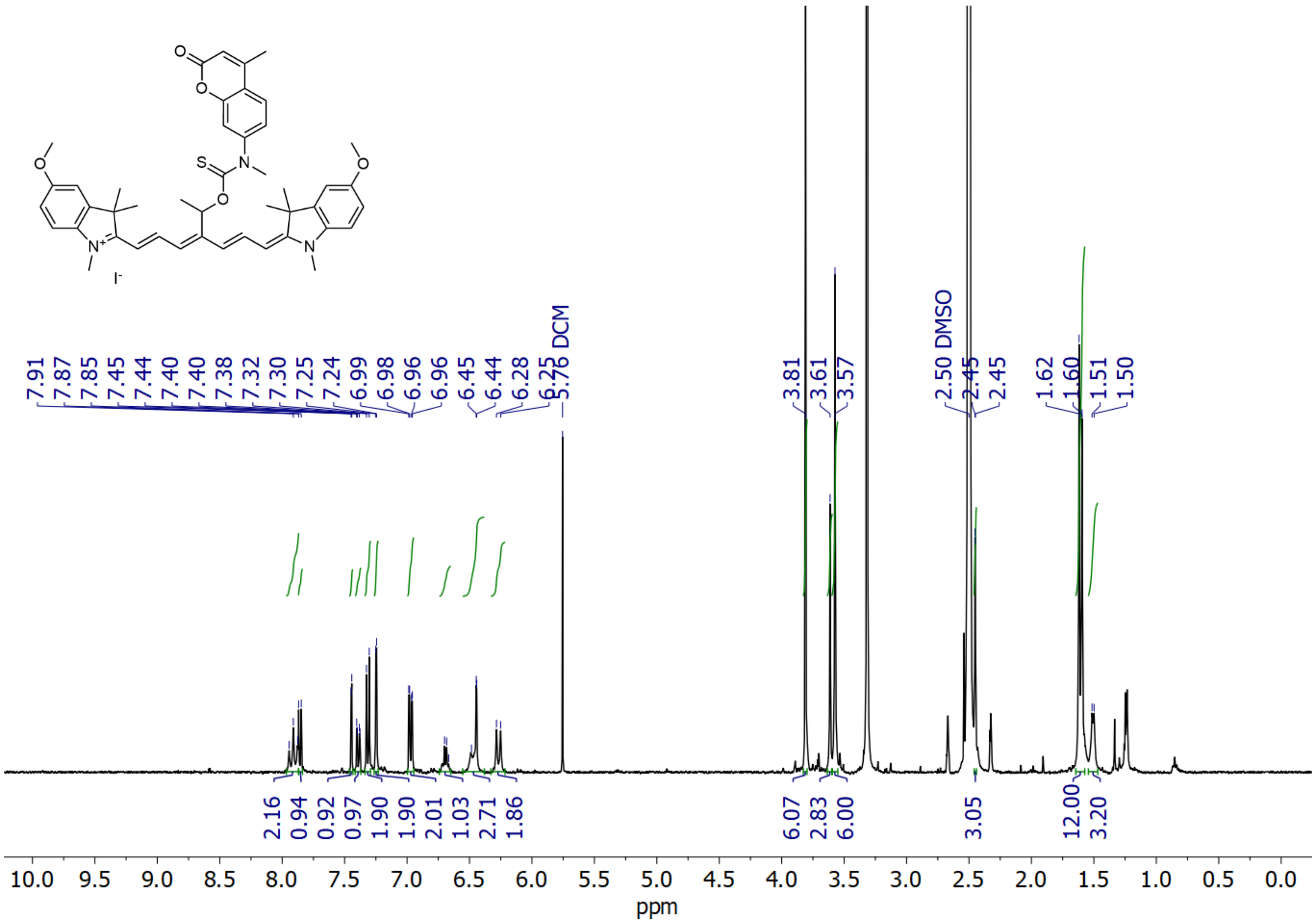

Figure S19. ^{1}H NMR (400 MHz, d_6-DMSO): **1a**

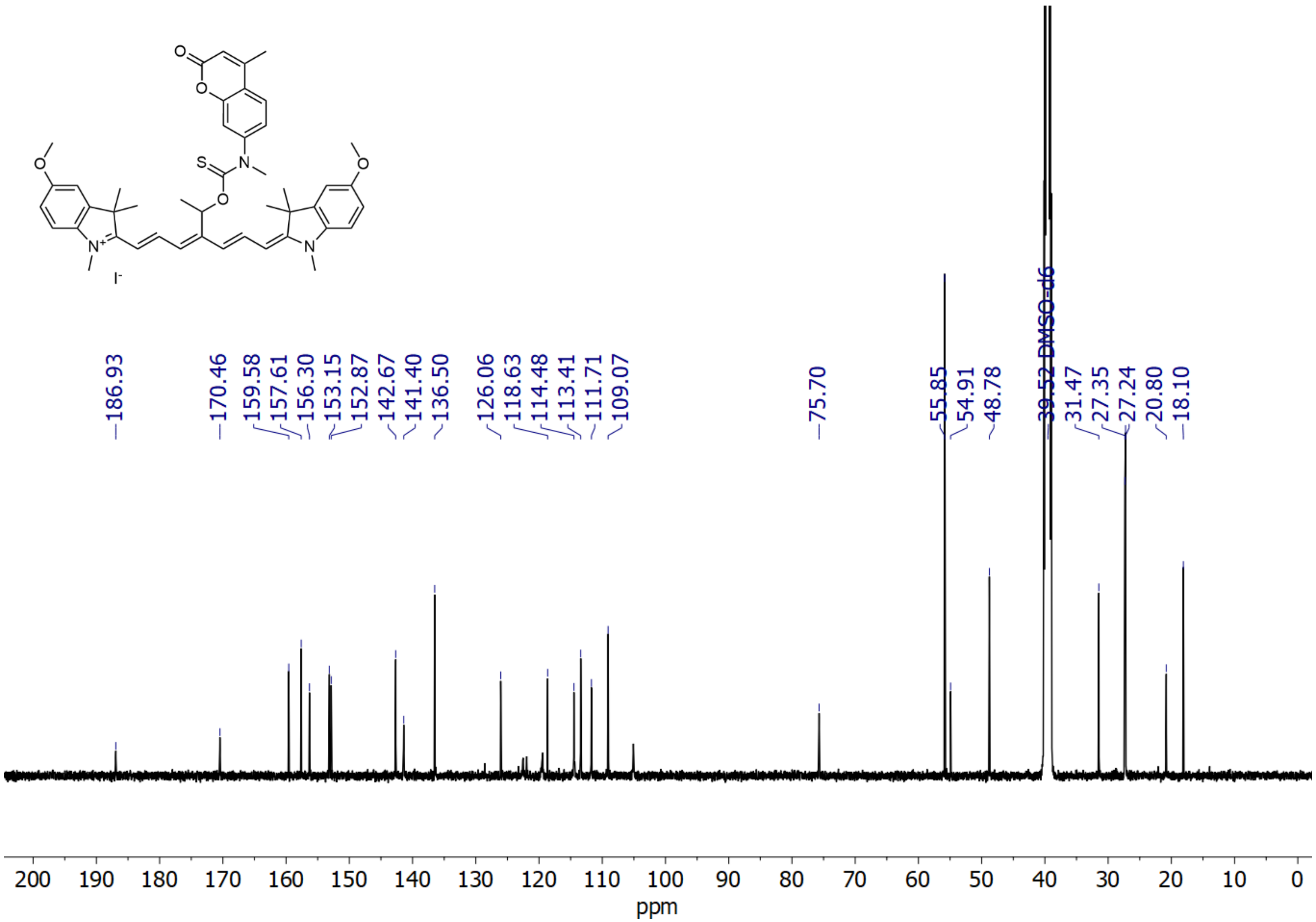

Figure S20. ^{13}C NMR (125 MHz, d_6-DMSO): **1a**

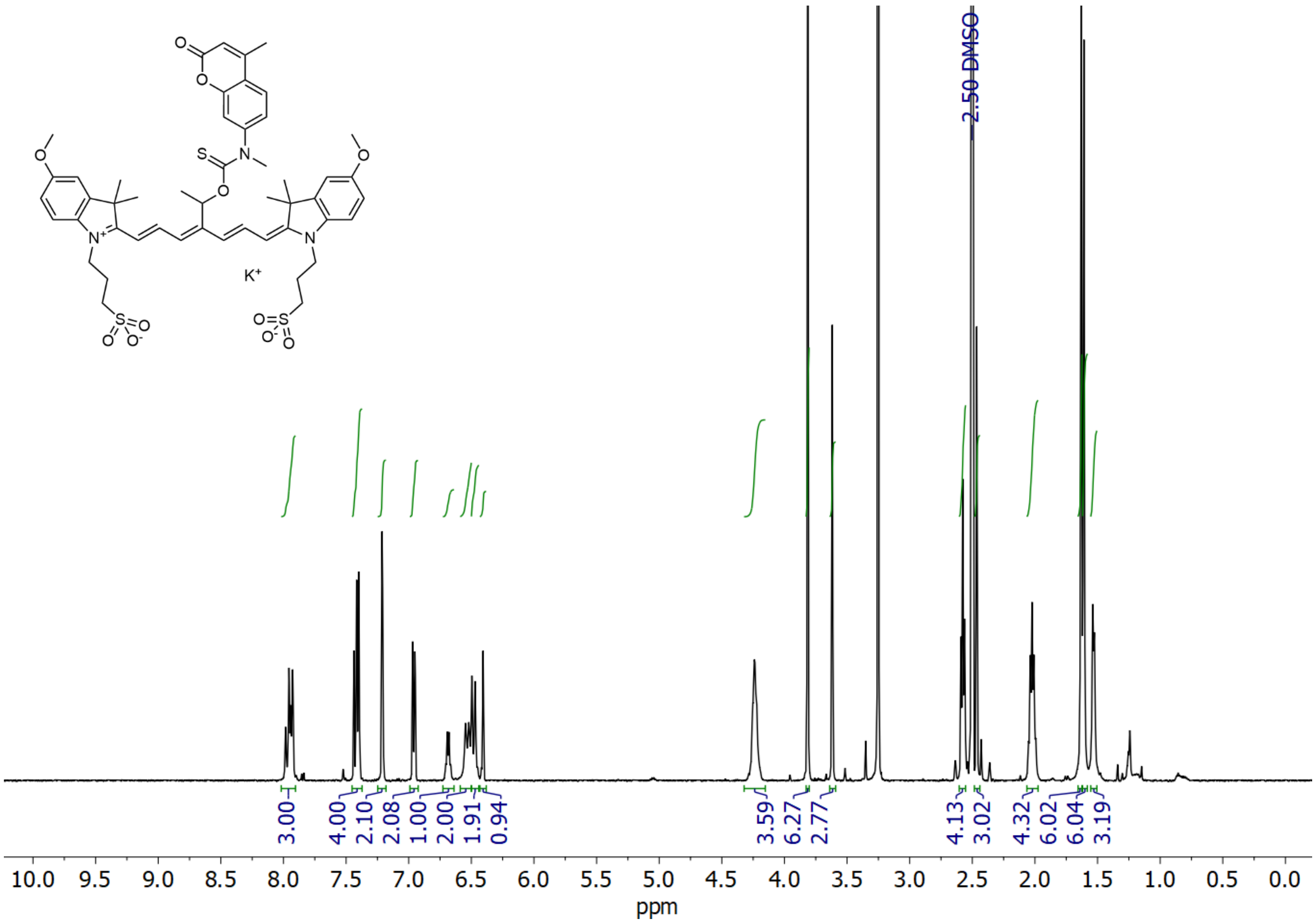

Figure S21. ^{1}H NMR (400 MHz, d_6-DMSO): **1c**

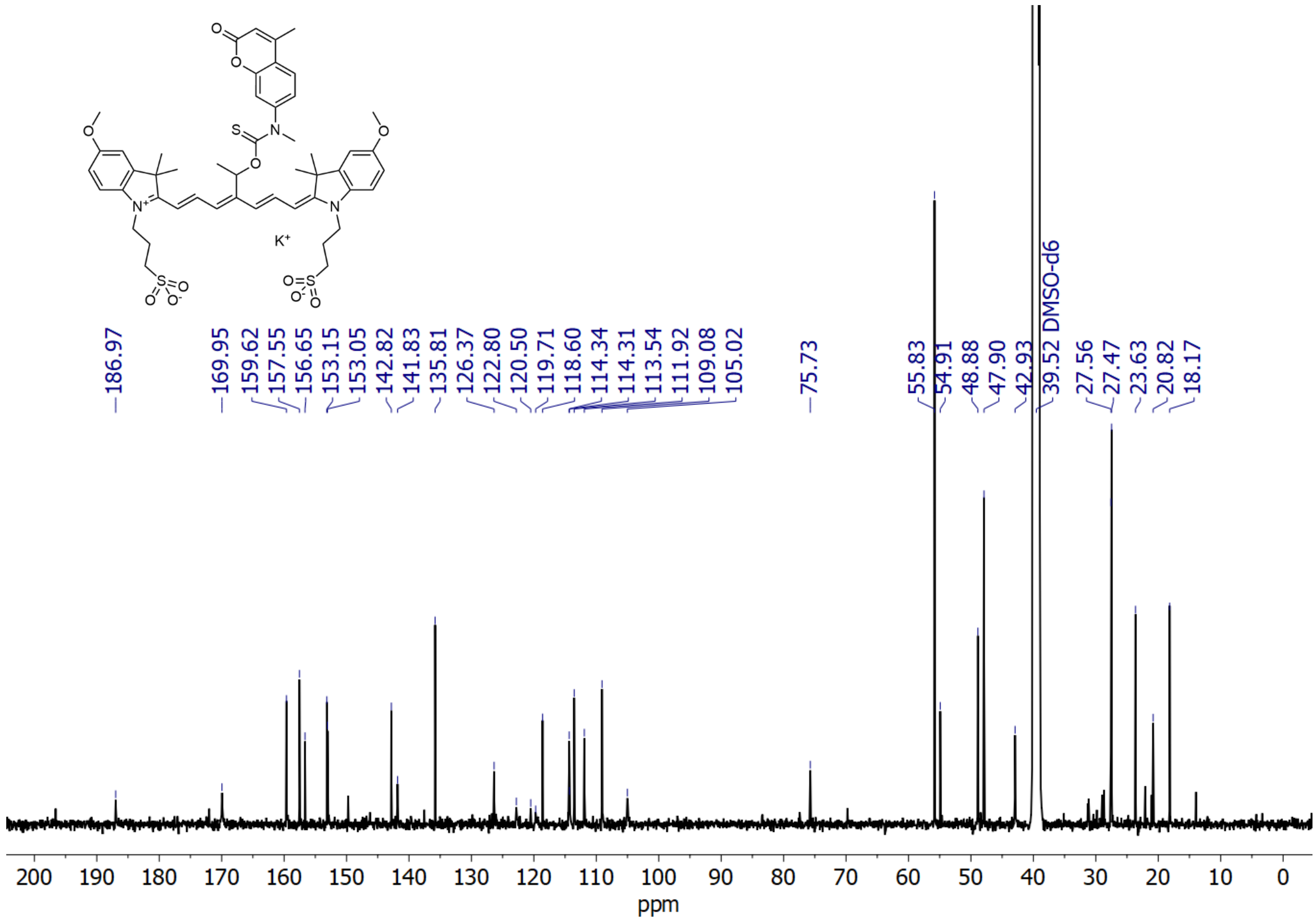

Figure S22. ^{13}C NMR (125 MHz, d_6-DMSO): **1c**

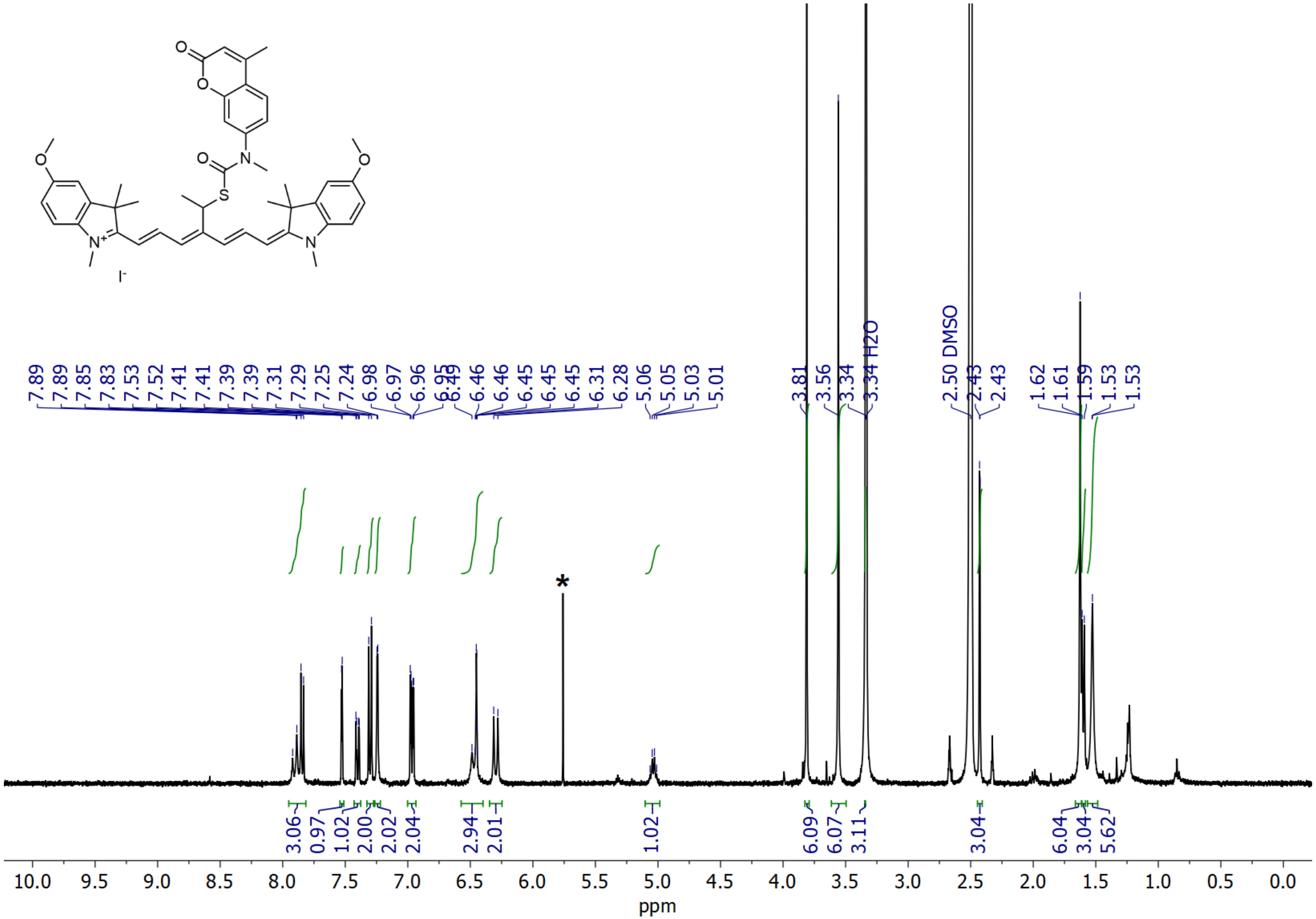

Figure S23. ^{1}H NMR (400 MHz, d_6-DMSO): **1b**, * CH_2Cl_2 δ (ppm) 5.76 (s).

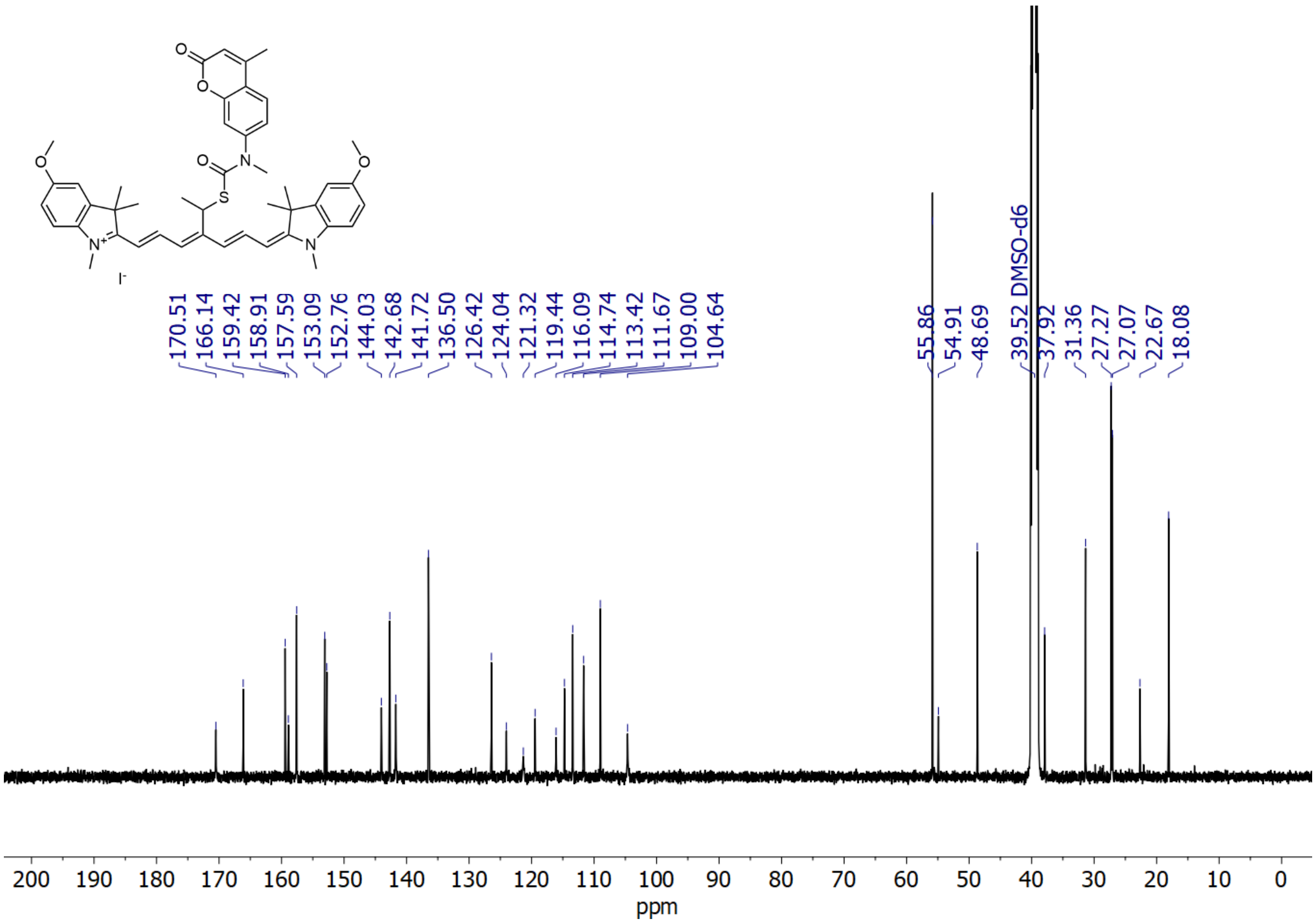

Figure S24. ^{13}C NMR (125 MHz, d_6-DMSO): **1b**

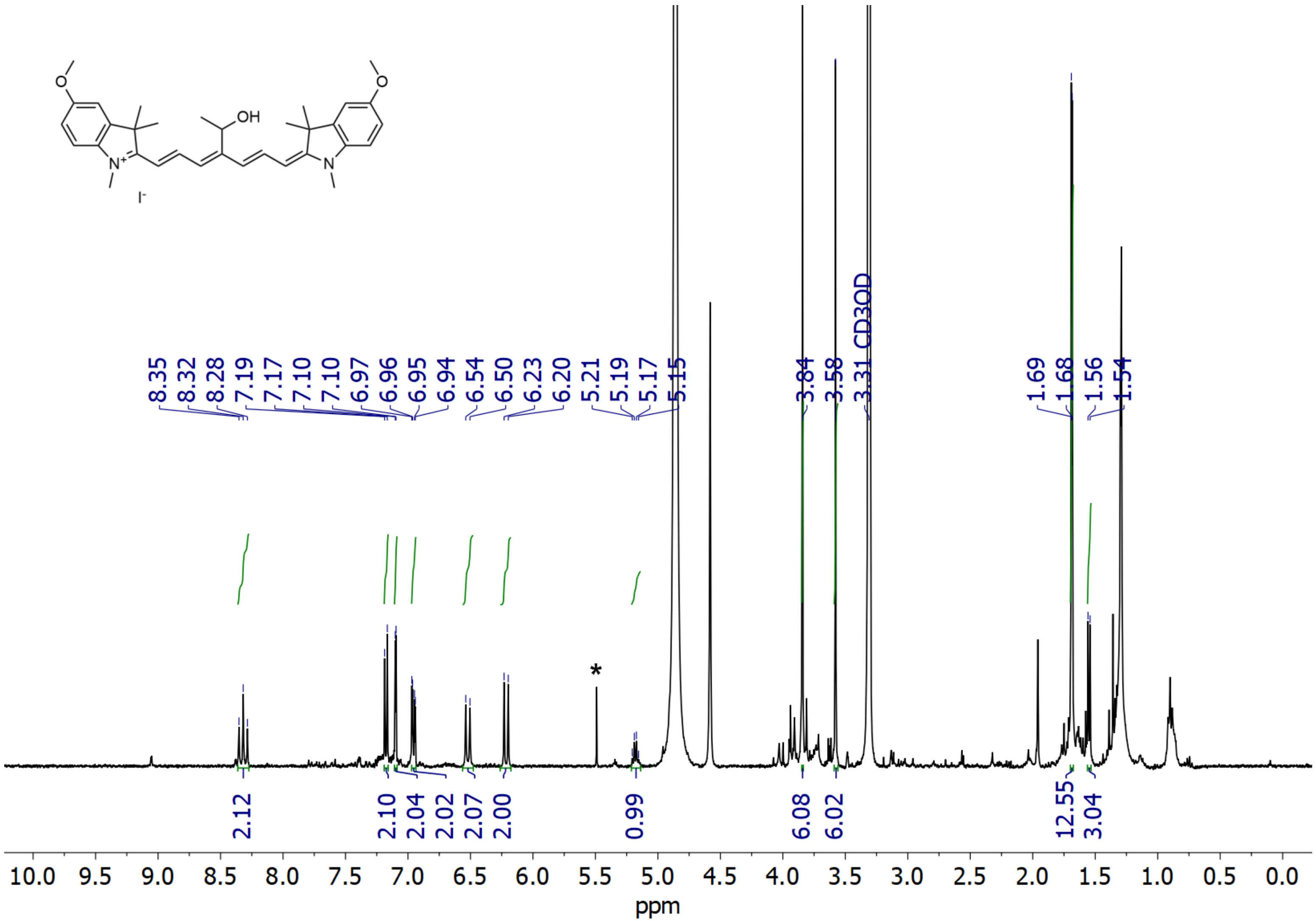

Figure S25. ^{1}H NMR (400 MHz, d_4-MeOH): **10,** * CH_2Cl_2 δ (ppm) 5.49 (s).

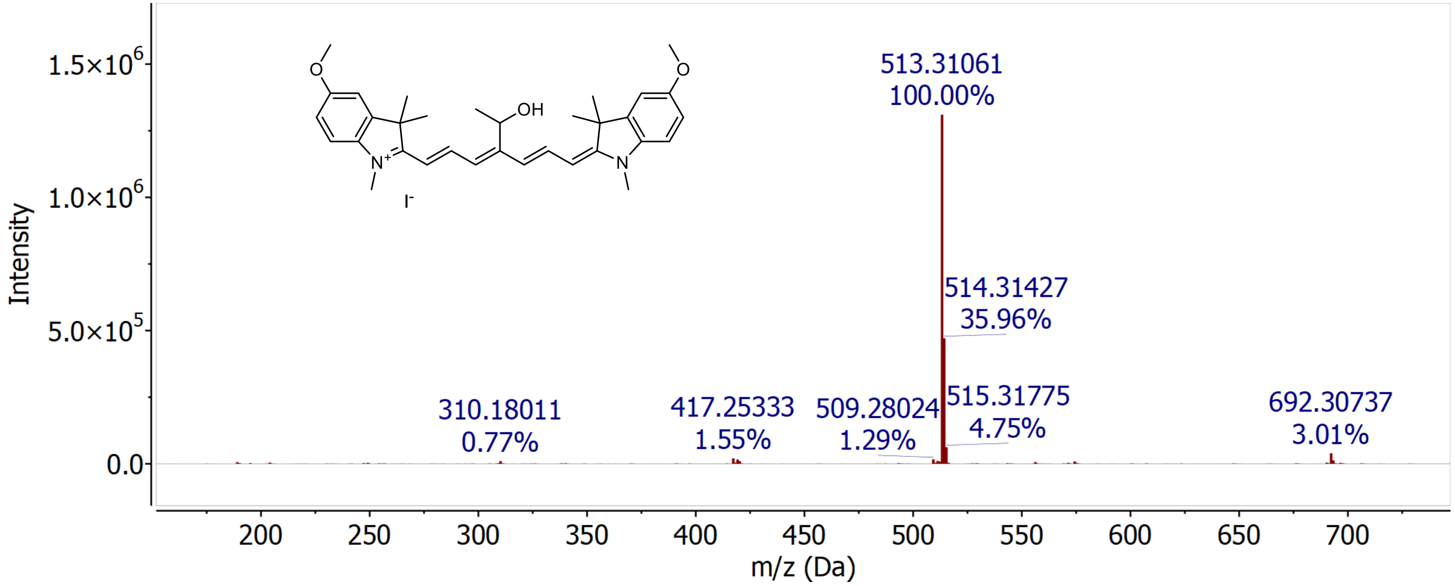

Figure S26. HRMS (ESI) spectrum of cyanine **10**.

UV-Vis Absorption and Emission Spectroscopy

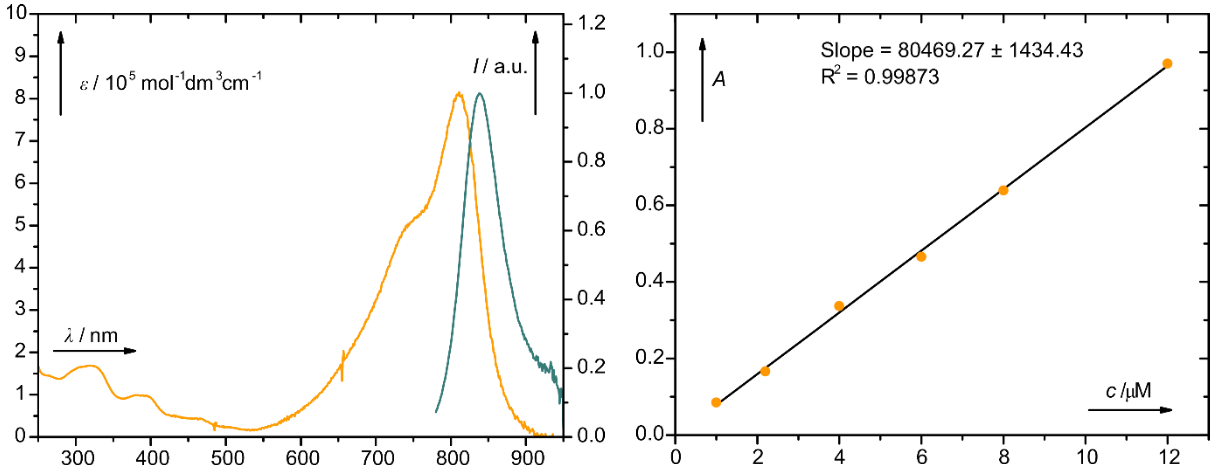

Figure S27. (left) UV-Vis absorption (red) and emission (blue) spectra of **1a** in HEPES (20 mM, pH = 7.4) with 10% of DMF. (right) Dependence of absorption at λ_{max} on the concentration of **1a** in HEPES (20 mM, pH = 7.4) with 10% of DMF.

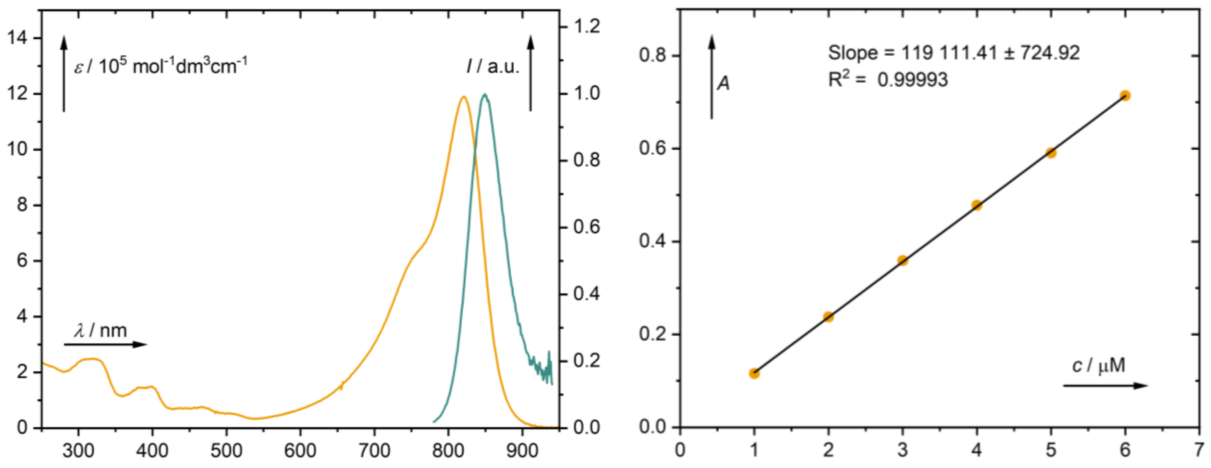

Figure S28. (left) UV-Vis absorption (red) and emission (blue) spectra of **1a** in MeOH. (right) Dependence of absorption at λ_{max} on the concentration of **1a** in MeOH.

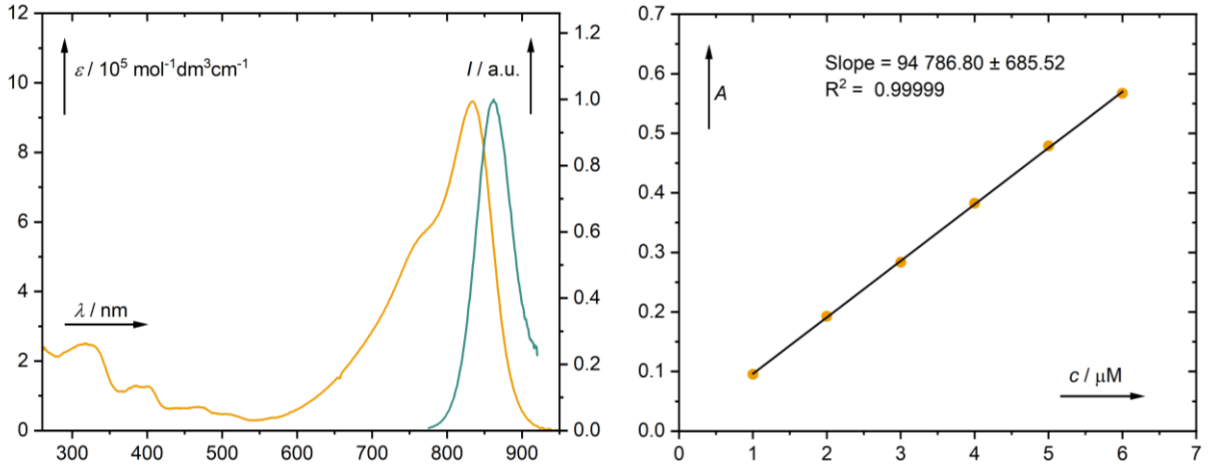

Figure S29. (left) UV-Vis absorption (red) and emission (blue) spectra of **1a** in DMSO. (right) Dependence of absorption at λ_{max} on the concentration of **1a** in DMSO.

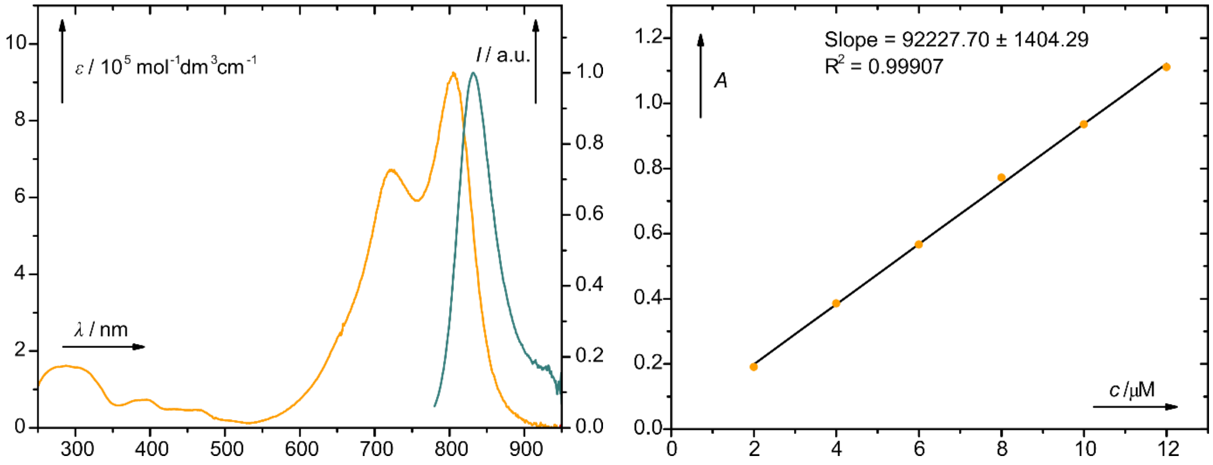

Figure S30. (left) UV-Vis absorption (red) and emission (blue) spectra of **1b** in HEPES (20 mM, pH = 7.4) with 10% of DMF. (right) Dependence of absorption at λ_{max} on the concentration of **1b** in HEPES (20 mM, pH = 7.4) with 10% of DMF.

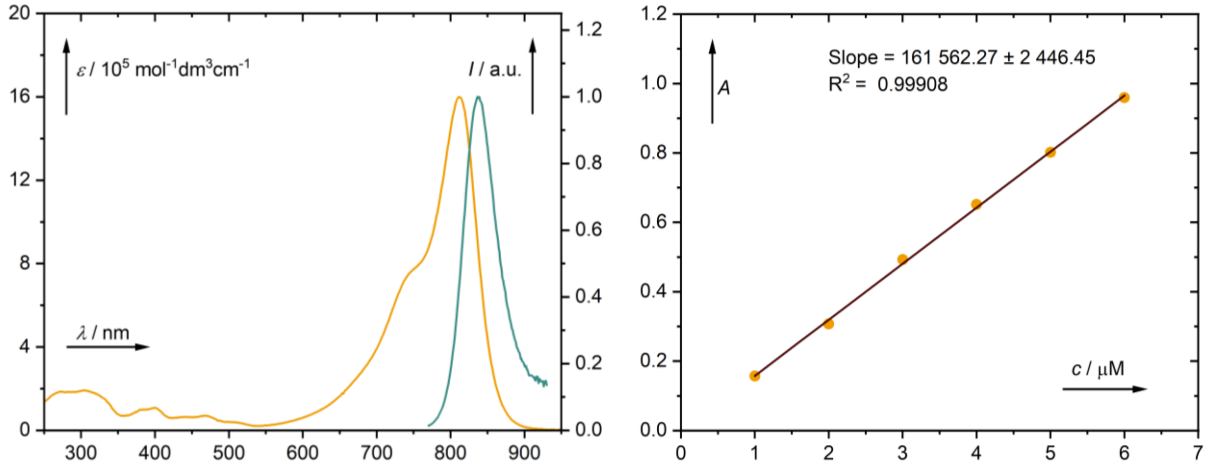

Figure S31. (left) UV-Vis absorption (red) and emission (blue) spectra of **1b** in MeOH. (right) Dependence of absorption at λ_{max} on the concentration of **1b** in MeOH.

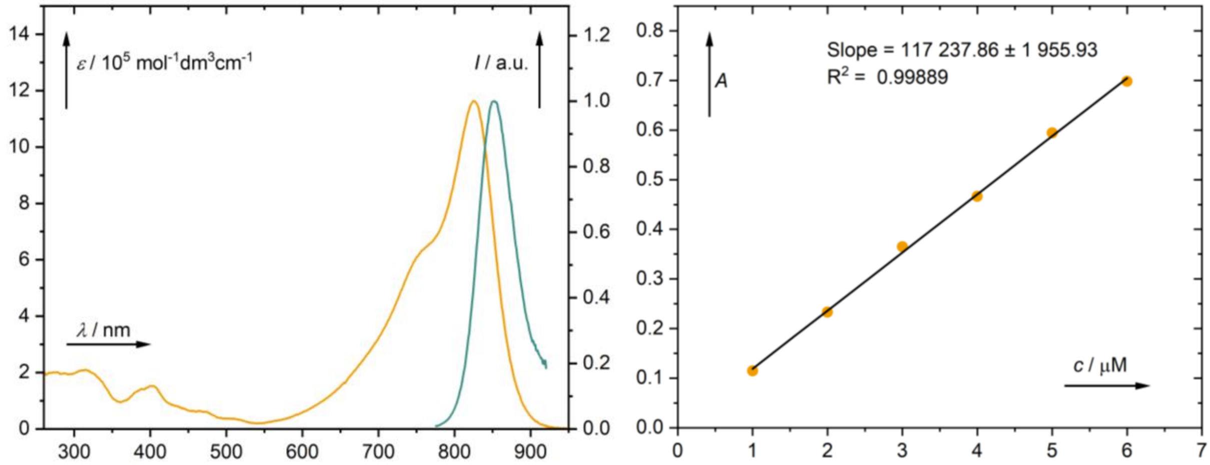

Figure S32. (left) UV-Vis absorption (red) and emission (blue) spectra of **1b** in DMSO. (right) Dependence of absorption at λ_{max} on the concentration of **1b** in DMSO.

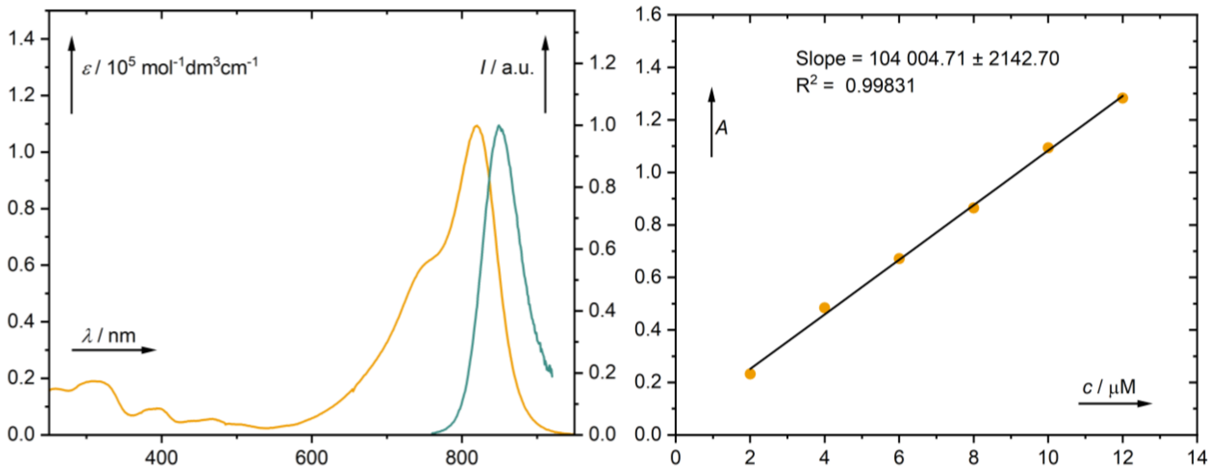

Figure S33. (left) UV-Vis absorption (red) and emission (blue) spectra of **1c** in HEPES (20 mM, pH = 7.4) with 10% of DMF. (right) Dependence of absorption at λ_{max} on the concentration of **1c** in HEPES (20 mM, pH = 7.4) with 10% of DMF.

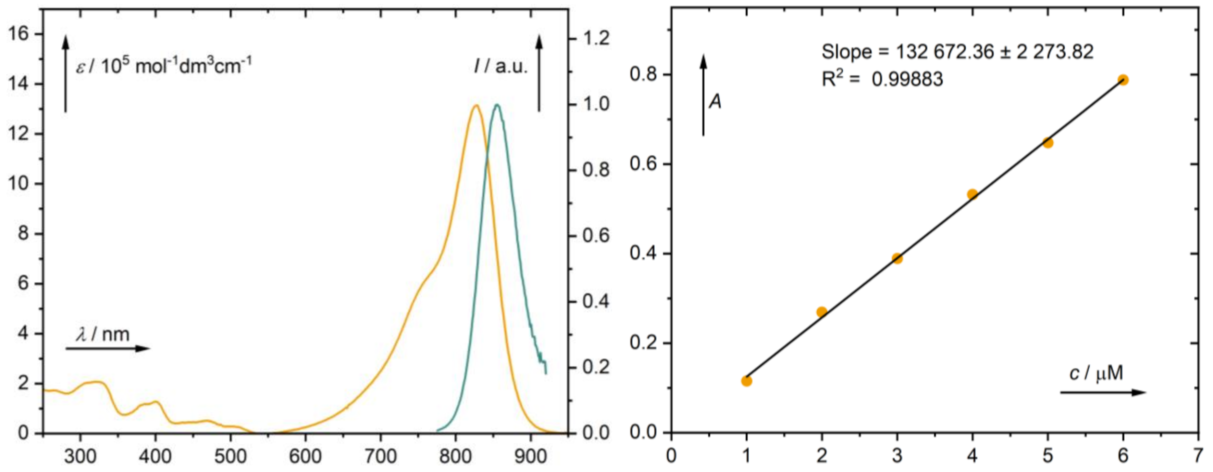

Figure S34. (left) UV-Vis absorption (red) and emission (blue) spectra of **1c** in MeOH. (right) Dependence of absorption at λ_{max} on the concentration of **1c** in MeOH.

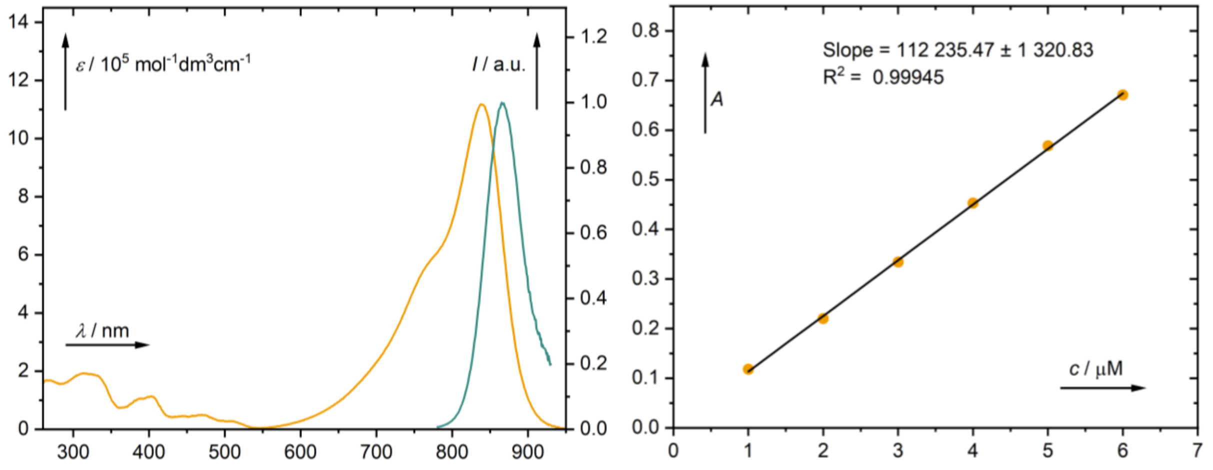

Figure S35. (left) UV-Vis absorption (red) and emission (blue) spectra of **1c** in DMSO. (right) Dependence of absorption at λ_{max} on the concentration of **1c** in DMSO.

Table S1. Summary table of the photophysical properties of photocages **1a-1c**.

	Solvent	λ_{abs}/nm	λ_{em}/nm	Stokes shift/nm	ε^{a}	Φ_F^{b}/%
	HEPES, 10% DMF	812	838	26	80 469	
**1a**	MeOH	820	849	29	119 111	<2
	DMSO	833	862	29	94 781	
	HEPES, 10% DMF	805	832	27	92 228	
**1b**	MeOH	812	836	24	161 562	<2
	DMSO	826	853	27	117 238	
	HEPES, 10% DMF	820	849	29	104 005	
**1c**	MeOH	828	855	27	132 672	<2
	DMSO	839	865	26	112 235	

[a]The molar absorption coefficient, ε_{max}/mol^{-1} dm^{3} cm^{-1}. [b]Quantum yield of fluorescence.

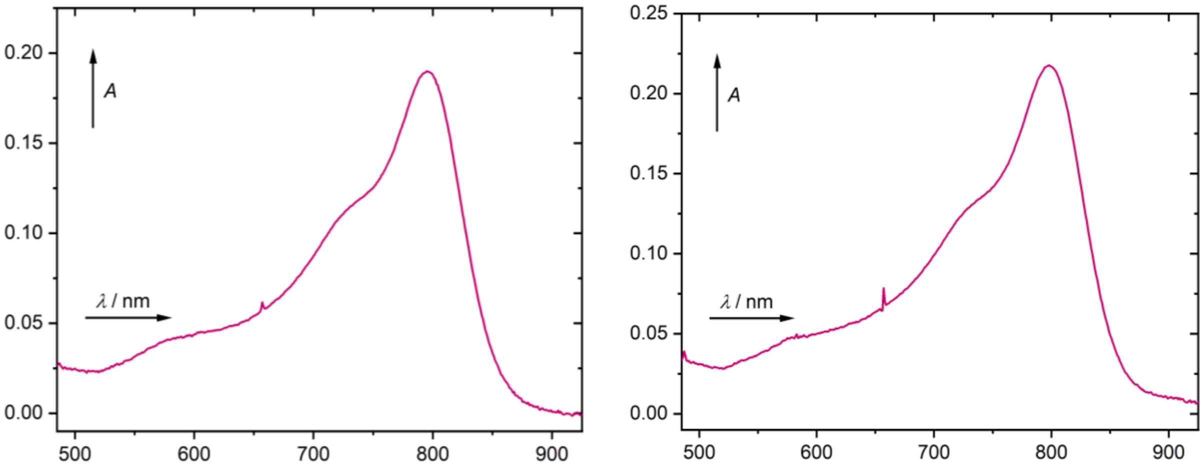

Figure S36. UV-Vis absorption spectra of **10** in HEPES (with 10% DMF) (left) and UV-Vis absorption spectra of **10** in DMEM (with 10% DMF).

Irradiation experiments

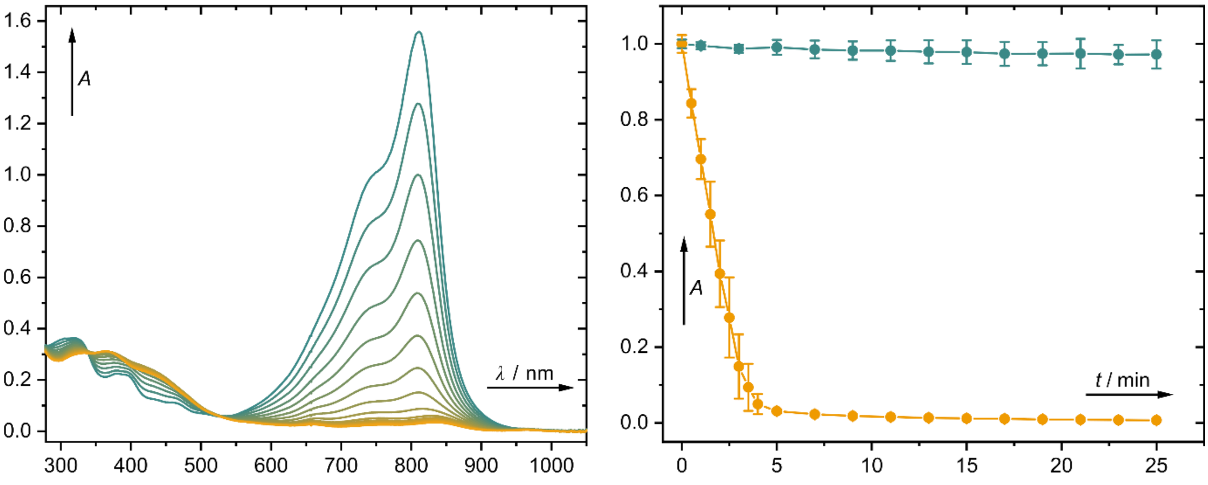

Figure S37. (left) Irradiation of **1a** (c ~ 20 × 10^{-6} M) at 820 nm in aerated HEPES (20 mM, pH = 7.4) with 10% of DMF followed by UV-vis spectroscopy in 30-s intervals for the first 5 min then 1-min intervals fo the subsequent 25 min (blue to red). (right) Kinetic traces measured at

absorption maxima for **1a** in dark (blue) and under irradiation at 820 nm (red) HEPES (20 mM, pH = 7.4) with 10% of DMF. Normalized to A = 1.0 at t = 0 min. The error bars represent standard deviation of the mean from three independent samples.

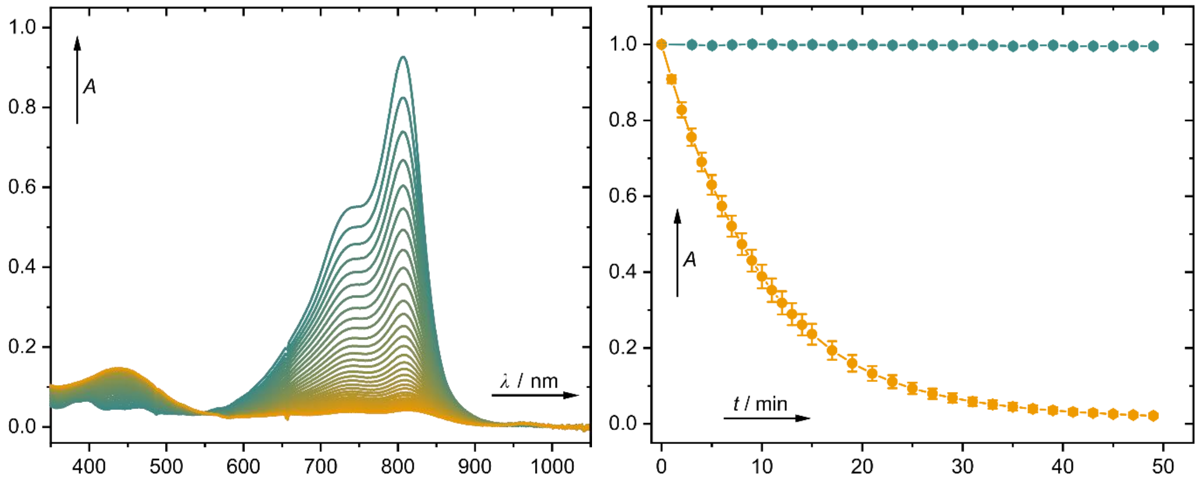

Figure S38. (left) Irradiation of **1b** (c ~ 18 × 10^{-6} M) at 820 nm in aerated HEPES (20 mM, pH = 7.4) with 15% of DMF followed by UV-vis spectroscopy in 1-min intervals (blue to red). (right) Kinetic traces measured at absorption maxima for **1a** in dark (blue) and under irradiation at 820 nm (red) HEPES (20 mM, pH = 7.4) with 15% of DMF. Normalized to A = 1.0 at t = 0 min. The error bars represent standard deviation of the mean from three independent samples.

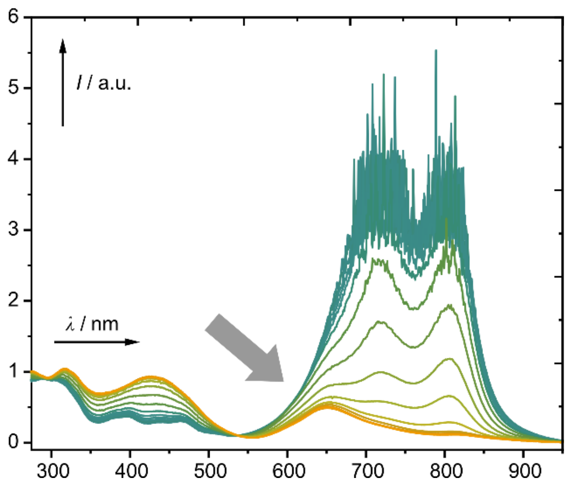

Figure S39. Irradiation of **1b** at high concentration (c ~ 45 × 10^{-6} M) at 820 nm in aerated HEPES (20 mM, pH = 7.4) with 10% of DMF followed by UV-vis spectroscopy in 60-s intervals. The arrow denotes formation of another species, presumably phototruncated Cy5.

Determination of Chemical Yield of Cargo Release

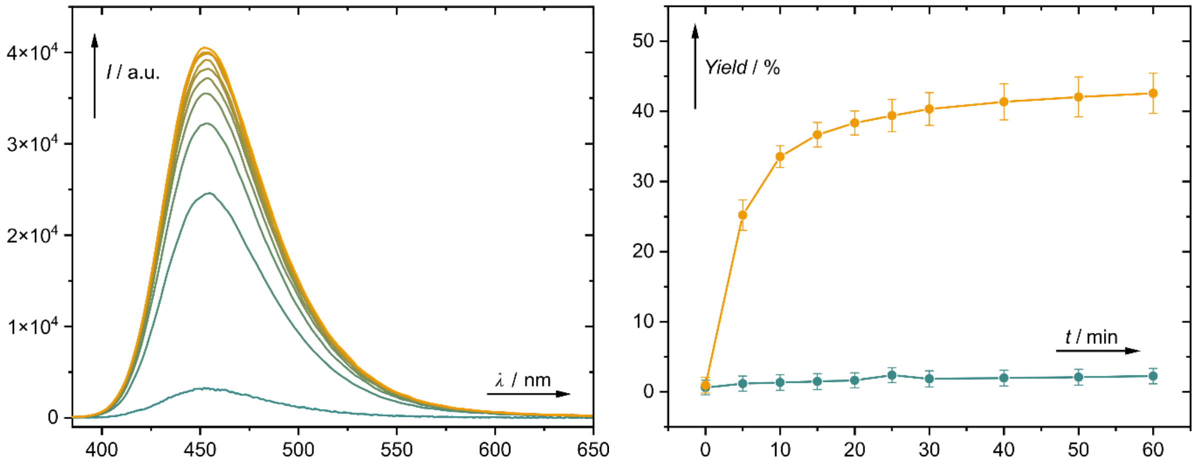

Figure S40. (left) Emission spectra of **1a** (c ~ 1.8 × 10^{-6} M) irradiated at 820 nm with λ_{exc} = 365 nm in aerated HEPES (20 mM, pH = 7.4 with 10% of DMF) in 5-min intervals for the first 30 minutes and then in 10-min intervals for the subsequent 30 minutes (blue to red). (right) Kinetic traces measured at λ = 365 nm for **1a** in dark (blue) and under irradiation at 820 nm (red) HEPES (20 mM, pH = 7.4) with 10% of DMF. The error bars represent standard deviation of the mean from three independent samples

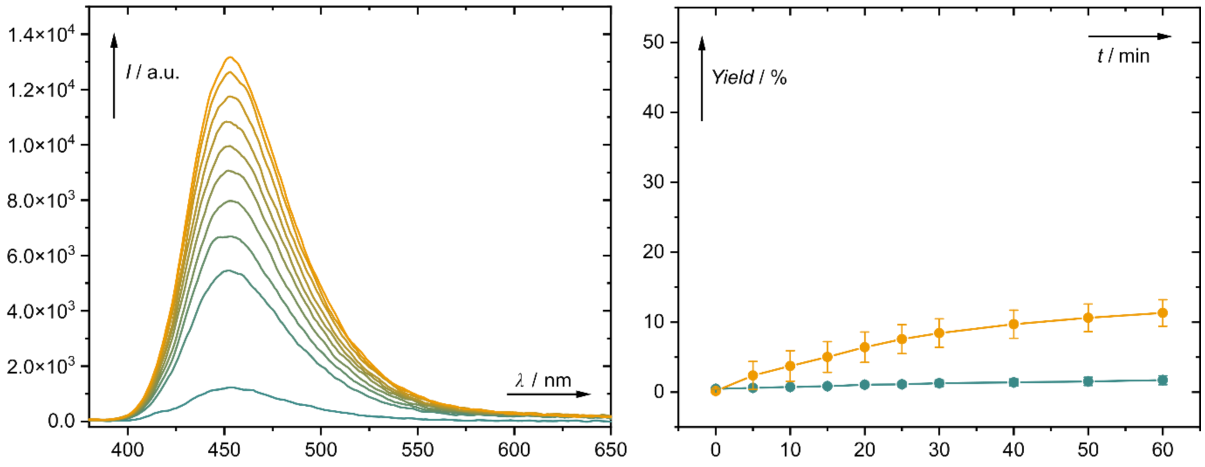

Figure S41. (left) Emission spectra of **13** (c ~ 1.6 × 10^{-6} M) irradiated at 820 nm with λ_{exc} = 365 nm in aerated HEPES (20 mM, pH = 7.4 with 10% of DMF) in 5-min intervals for the first 30 minutes and then in 10-min intervals for the subsequent 30 minutes (blue to red). (right) (right) Kinetic traces measured at λ = 365 nm for **1a** in dark (blue) and under irradiation at 820 nm (red) HEPES (20 mM, pH = 7.4) with 10% of DMF. The error bars represent standard deviation of the mean from three independent samples

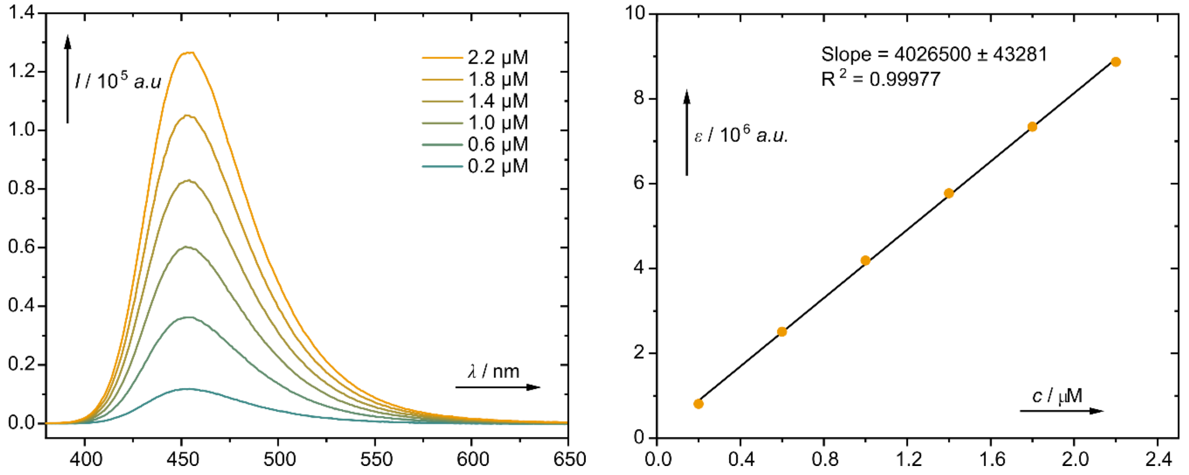

Figure S42. (right) Stacked emission spectra of **2** with concentrations from c~0.2 × 10^{-6} M to ~ 2.2 × 10^{-6} M. (left) Representative calibration curve of **2** in aerated HEPES (20 mM, pH = 7.4 with 10% of DMF).

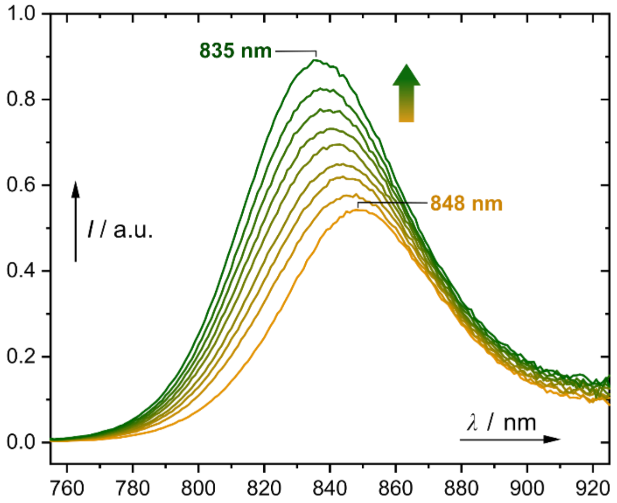

Figure S43. Emission spectra of **1a** (c ~ 1.8 × 10^{-6} M) irradiated at 810 nm with λ_{exc} = 747 nm in MeOH purged with Ar for 30 minutes (orange to green).

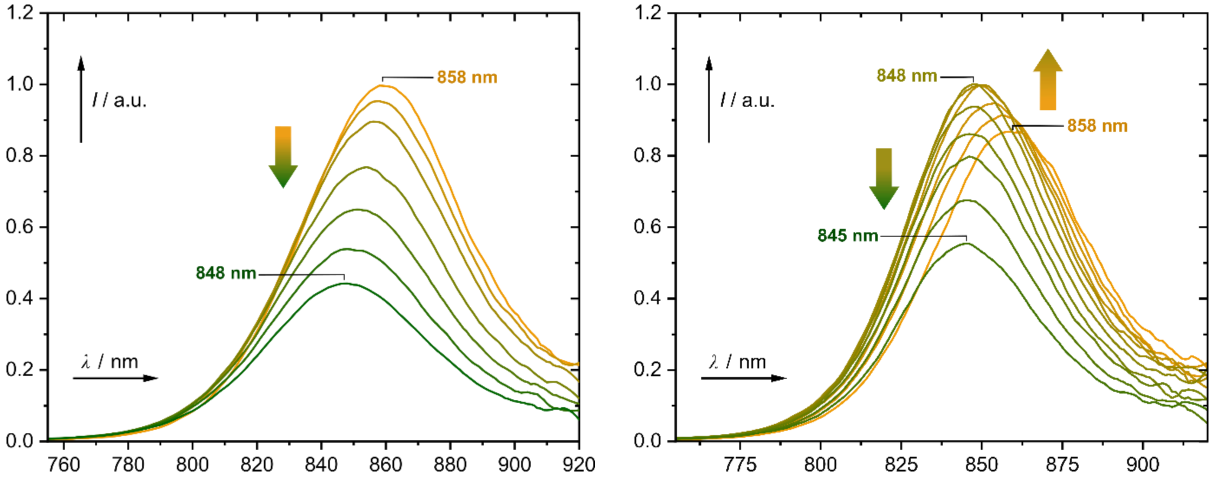

Figure S44. Emission spectra (λ_{exc} = 747 nm) of **1a** (c ~ 1.8 × 10^{-6} M) irradiated at 810 nm in DMEM (with 10% DMF) aerated (left) and purged with Ar (right) for 10 minutes (orange to green).

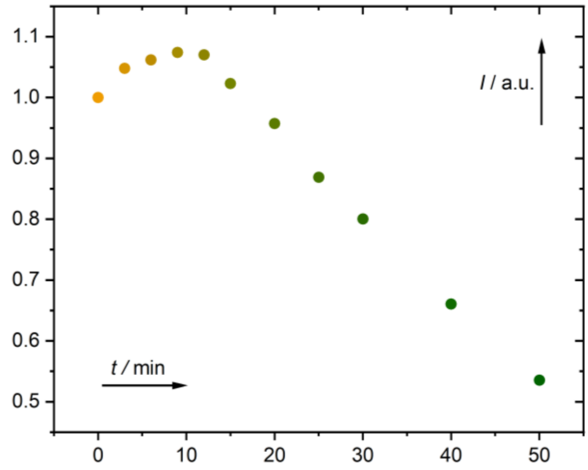

Figure S45. Trace of emission maxima (λ_{exc} = 747 nm) of **1a** (c ~ 1.8 × 10^{-6} M) irradiated at 810 nm in DMEM (with 10% DMF) purged with Ar for 10 minutes (orange to green); same experiment as Figure S63. Initial increase followed by adecrease of intensity can be observed.

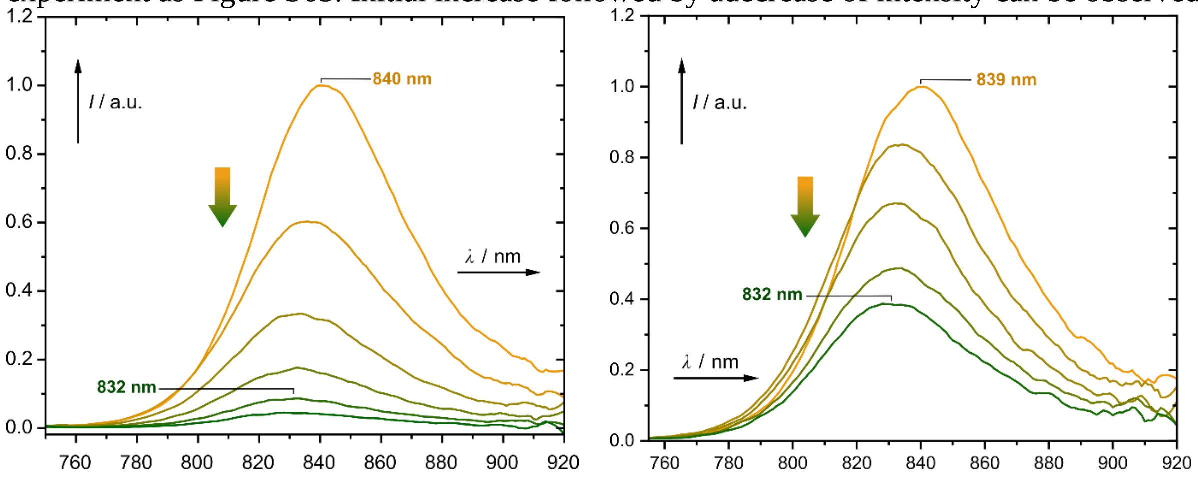

Figure S46. Emission spectra of **1a** (c ~ 1.8 × 10^{-6} M) irradiated at 810 nm with λ_{exc} = 747 nm in HEPES (with 10% DMF) aerated (left) and purged with Ar (right) for 10 minutes (orange to green).

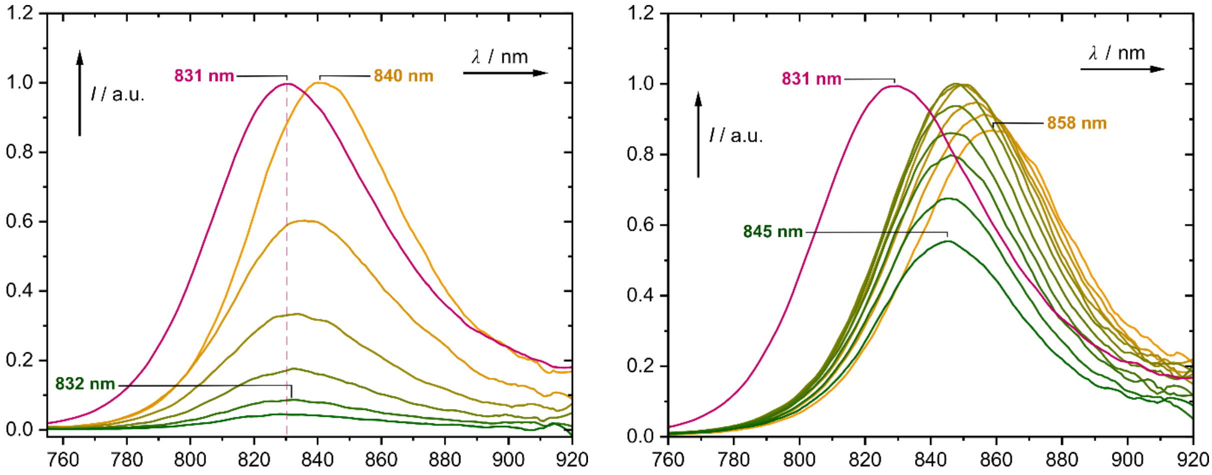

Figure S47. Emission spectra of **1a** (c ~ 1.8 × 10^{-6} M) irradiated at 810 nm with λ_{exc} = 747 nm in HEPES (with 10% DMF) aerated (orange to green) overlapped with emission spectra of **10** in HEPES (with 10% DMF) (magenta) (left) and **e**mission spectra of **1a** (c ~ 1.8 × 10^{-6} M) irradiated at 810 nm with λ_{exc} = 747 nm in DMEM (with 10% DMF) purged with Ar for 10 minutes (orange to green) overlapped with emission spectra of **10** in DMEM (with 10% DMF) (magenta) (right).

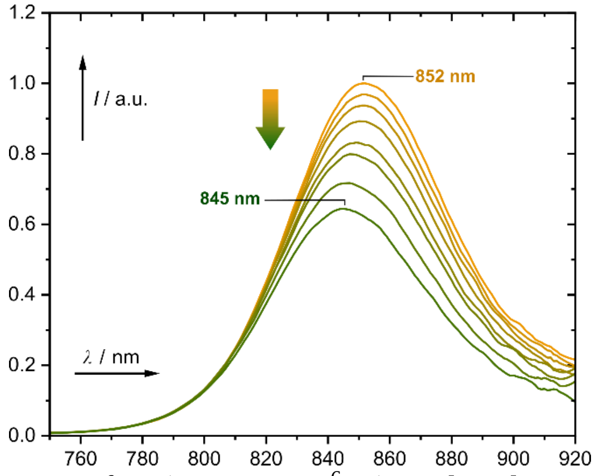

Figure S48. Emission spectra of **1a** (c ~ 1.8 × 10^{-6} M) irradiated at 810 nm with λ_{exc} = 747 nm in HEPES with BSA (30 mg mL^{-1}), with 10% DMF as co-solvent) purged with Ar (right) for 10 minutes (orange to green).

NMR Irradiation Experiments

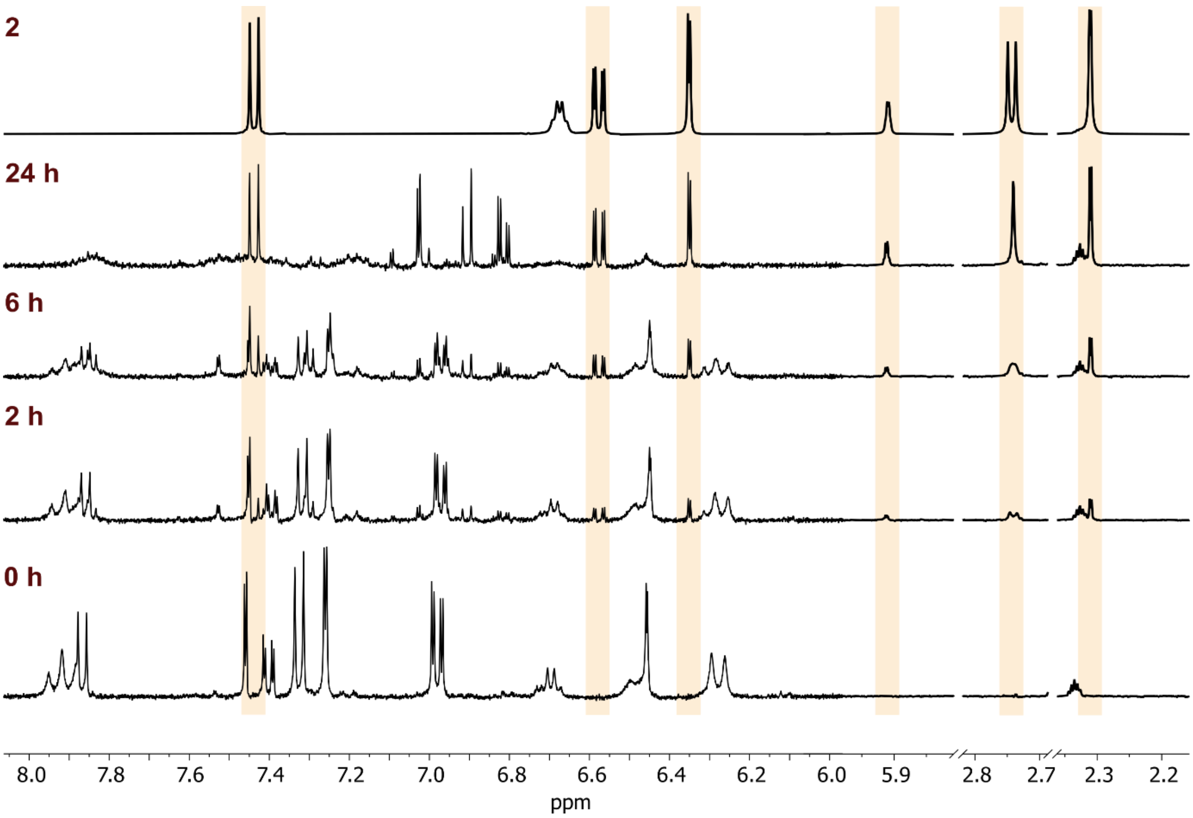

Figure S49. ^{1}H NMR (400 MHz, d_6-DMSO) of photocage **1a** irradiated in d_6-DMSO at 820 nm for indicated time, and comparison with reference payload **2**.

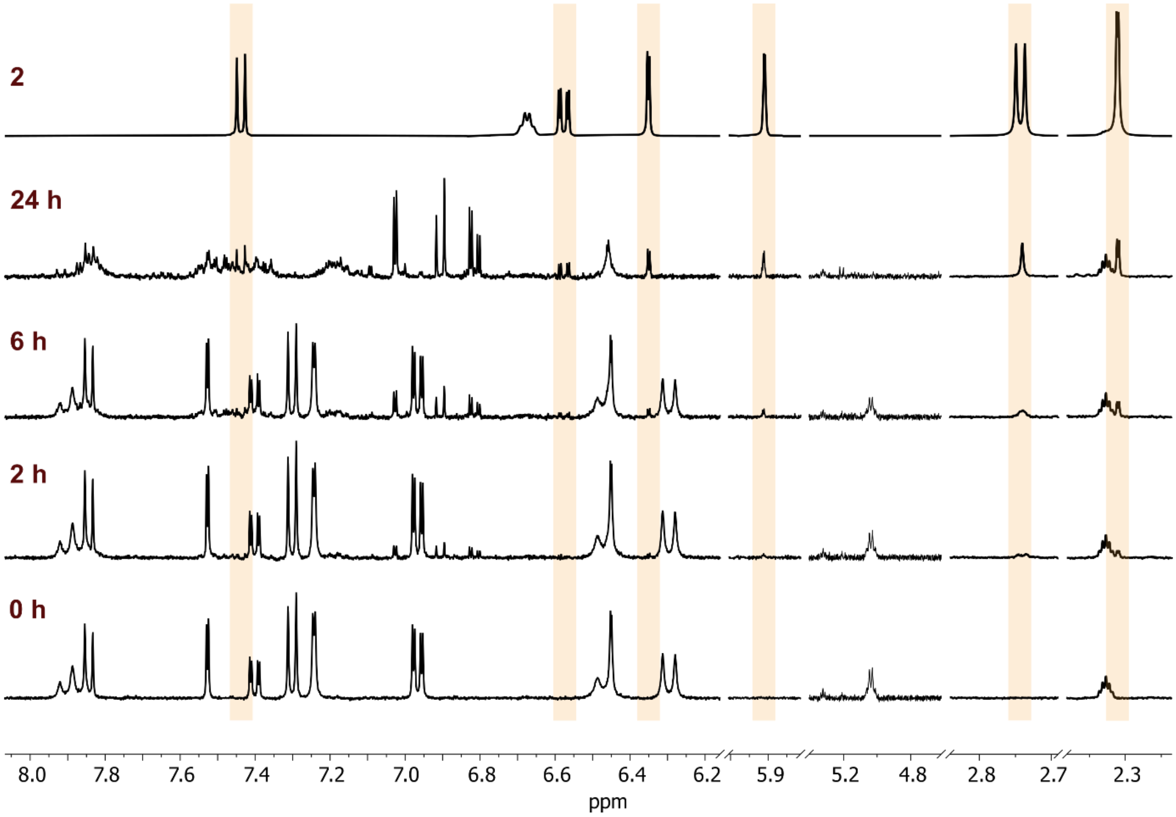

Figure S50. ^{1}H NMR (400 MHz, d_6-DMSO) and photocage **1b** irradiated in d_6-DMSO at 820 nm for indicated time, and comparison with reference payload **2**.

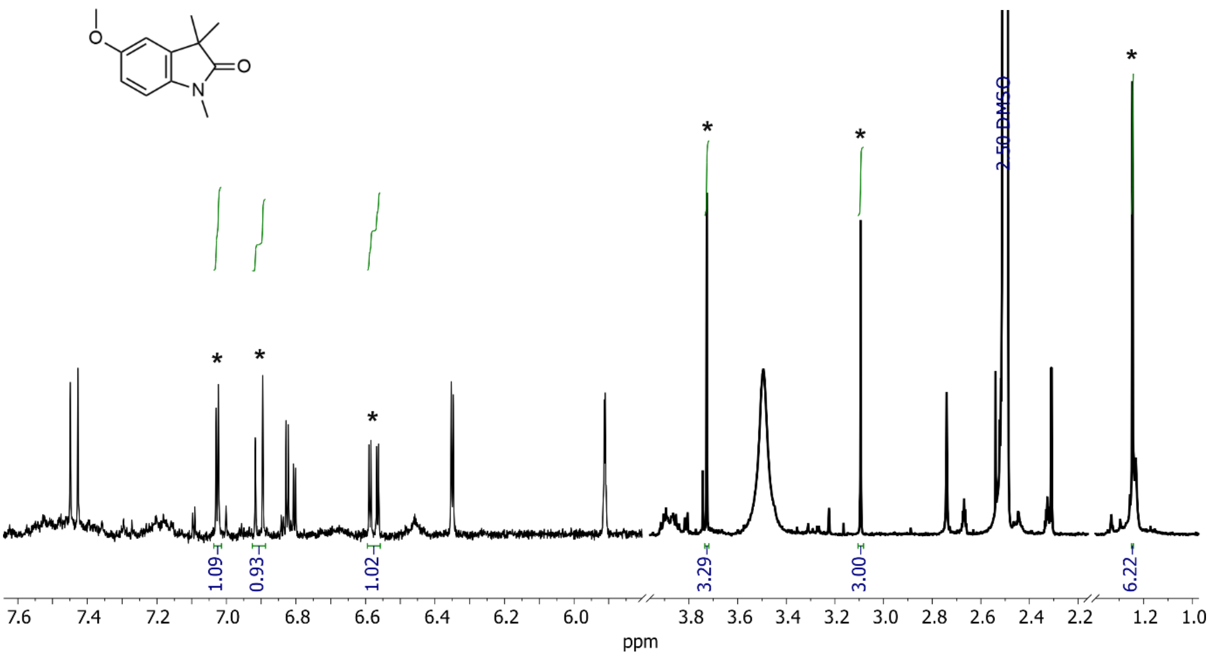

Figure S51. ^{1}H NMR (400 MHz, d_6-DMSO) of **1a** irradiated at 820 nm (40 mW) in d_6-DMSO for 24 h. Asterisks denote signals corresponding to ketone photoproduct of the photooxidation process which are in agreement with the literature values: ^{1}H NMR (400 MHz, d_6-DMSO) δ (ppm) 7.03 (d, J = 2.6 Hz, 1H), 6.91 (d, J = 8.4, 1.0 Hz, 1H), 6.81 (dd, J = 8.4, 2.6 Hz, 1H), 3.73 (s, 3H), 3.09 (s, 3H), 1.25 (s, 6H).[7]

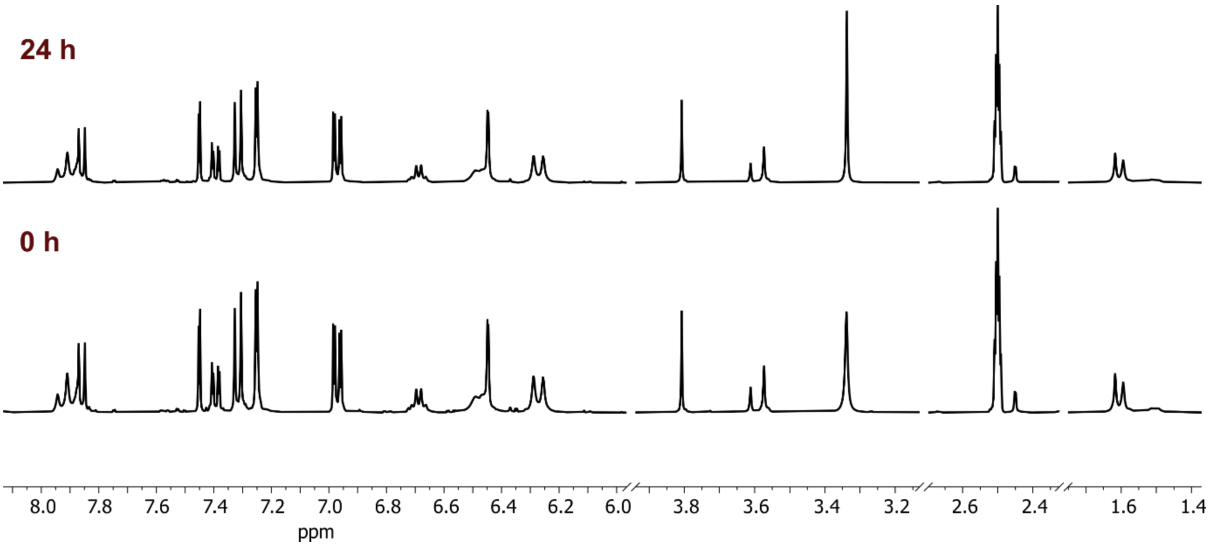

Figure S52. ^{1}H NMR (400 MHz, d_6-DMSO) of photocage **1a** d_6-DMSO kept in dark for 24 h.

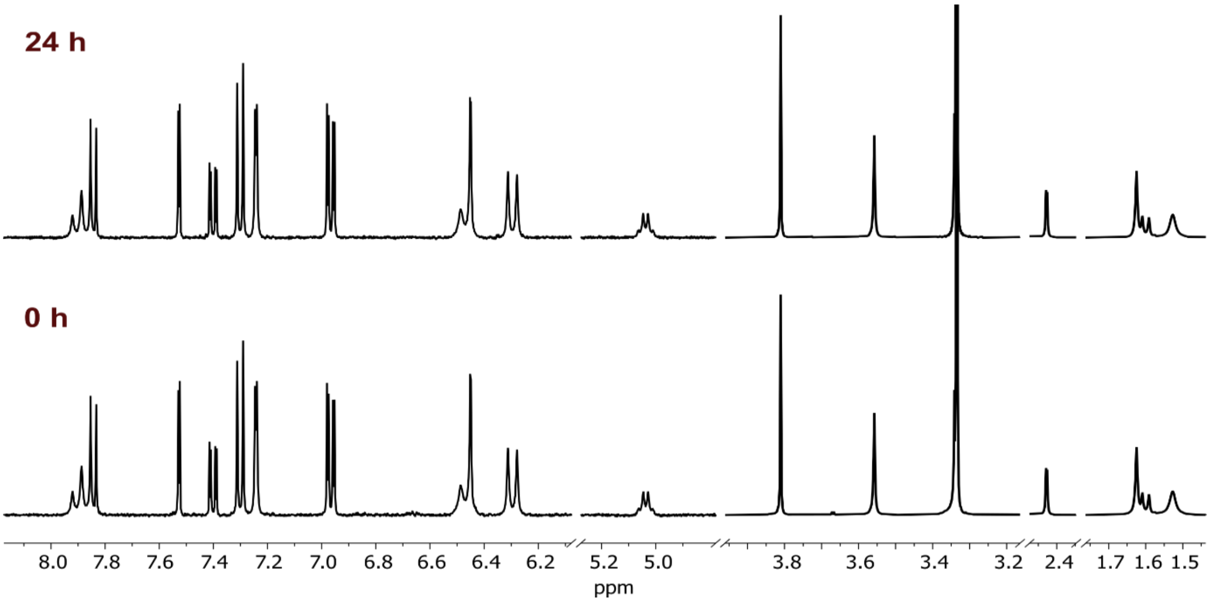

Figure S53. ^{1}H NMR (400 MHz, d_6-DMSO) of photocage **1b** d_6-DMSO kept in dark for indicated time.

Irradiation Experiments Under Oxygen-Free Conditions

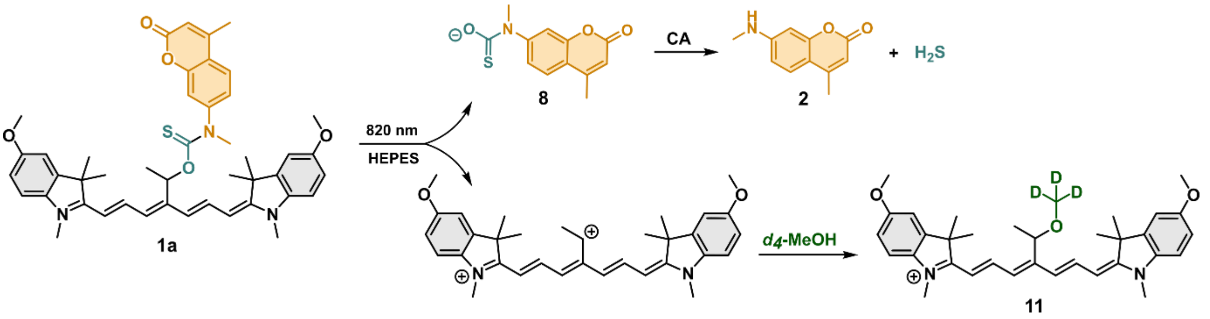

Scheme S3. Schematic representation of uncaging of **2** and H_2S from **1a** through the thiocarbamate 8 intermediate and formation of a trapped cation **11**.

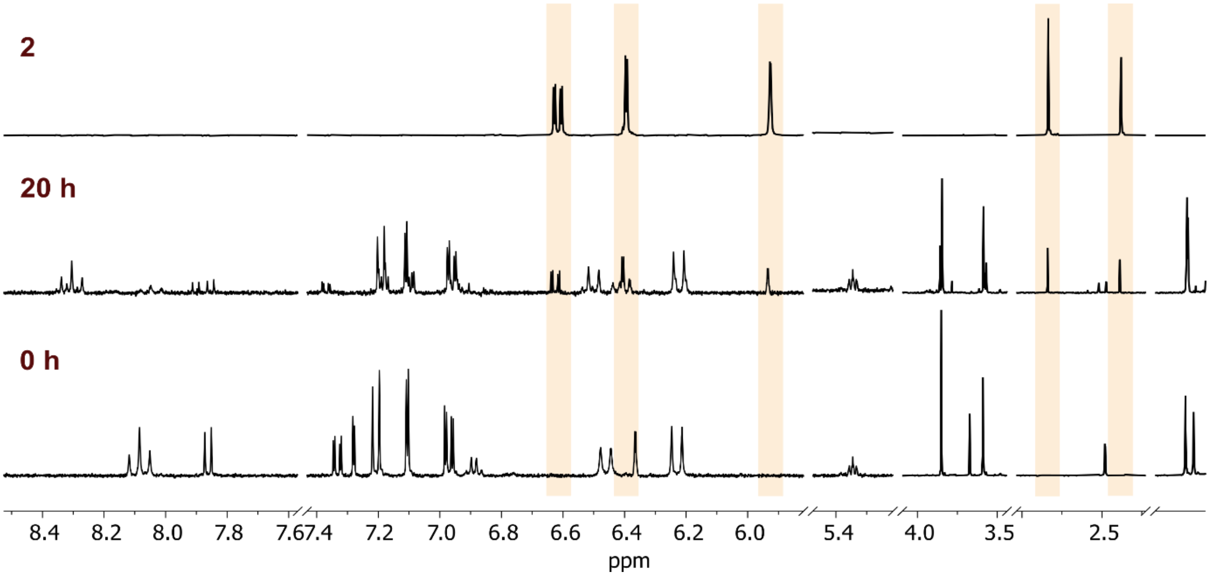

Figure S54. ^{1}H NMR (400 MHz, d_4-MeOH) photocage **1a** irradiated at 810 nm in degassed d_4-CD_3OD for 20 h, and comparison with the reference payload **2**.

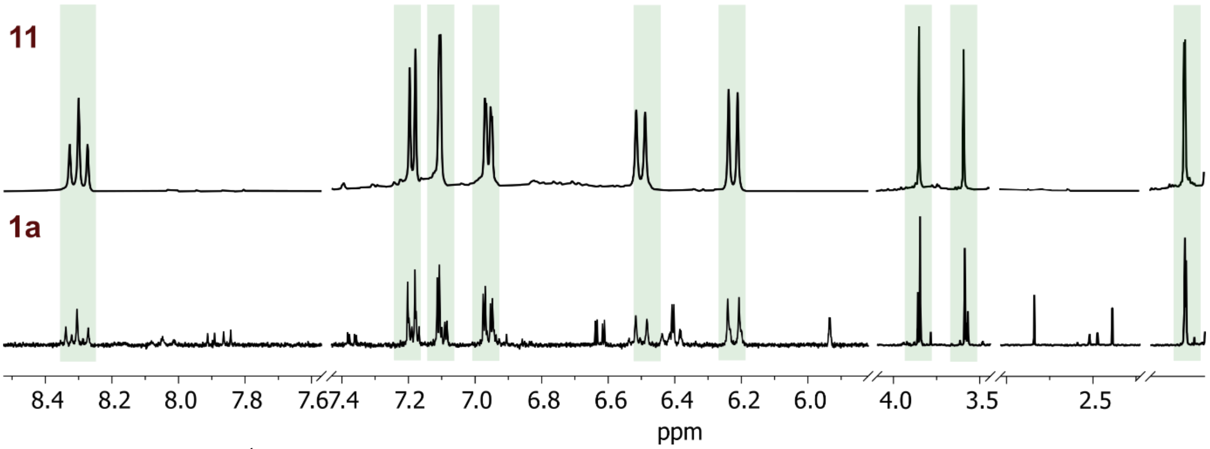

Figure S55. (top) ^{1}H NMR (400 MHz, d_4-MeOH) of a reference solvent trapped cation **11**. (bottom) ^{1}H NMR (400 MHz, d_4-MeOH) of photocage **1a** irradiated at 810 nm in d_4-CD_3OD for 20 h in oxygen-free conditions.

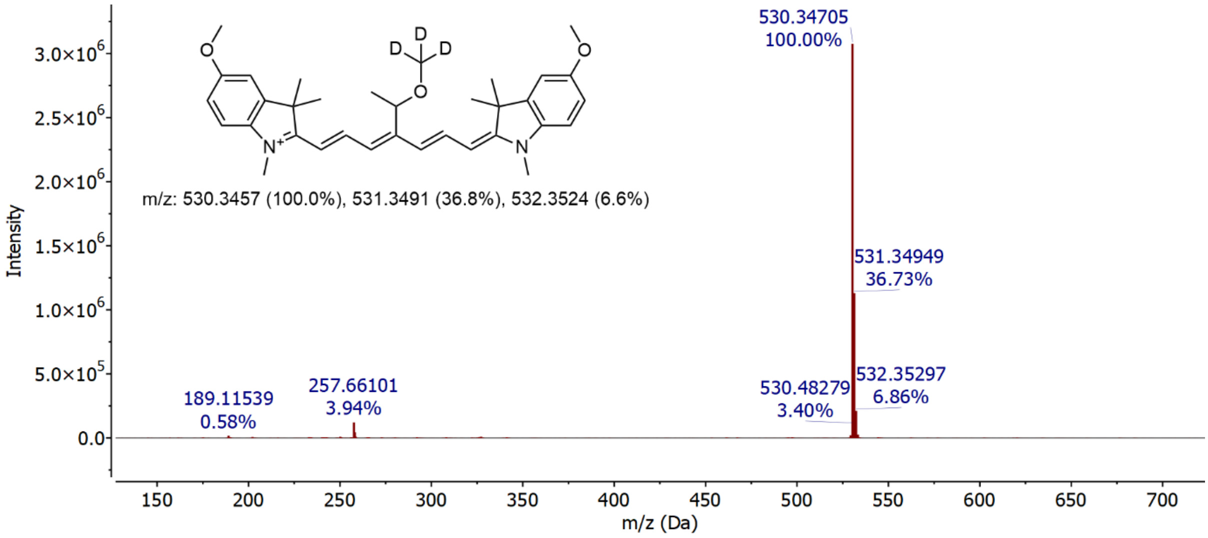

Figure S56. HRMS (ESI) spectrum of the sample of photocage **1a** irradiated in d_4-CD_3OD for 20 h under the exclusion of oxygen.

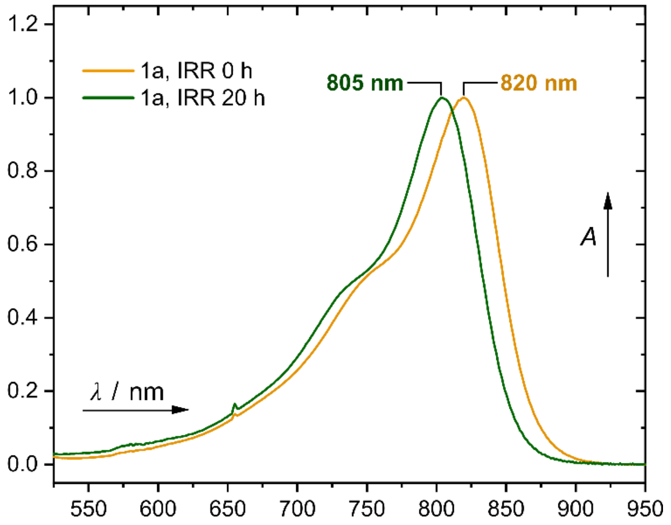

Figure S57. UV-vis spectrum sample of photocage **1a** irradiated in d_4-CD_3OD for 20 h under the exclusion of oxygen (diluted after irradiation with MeOH).

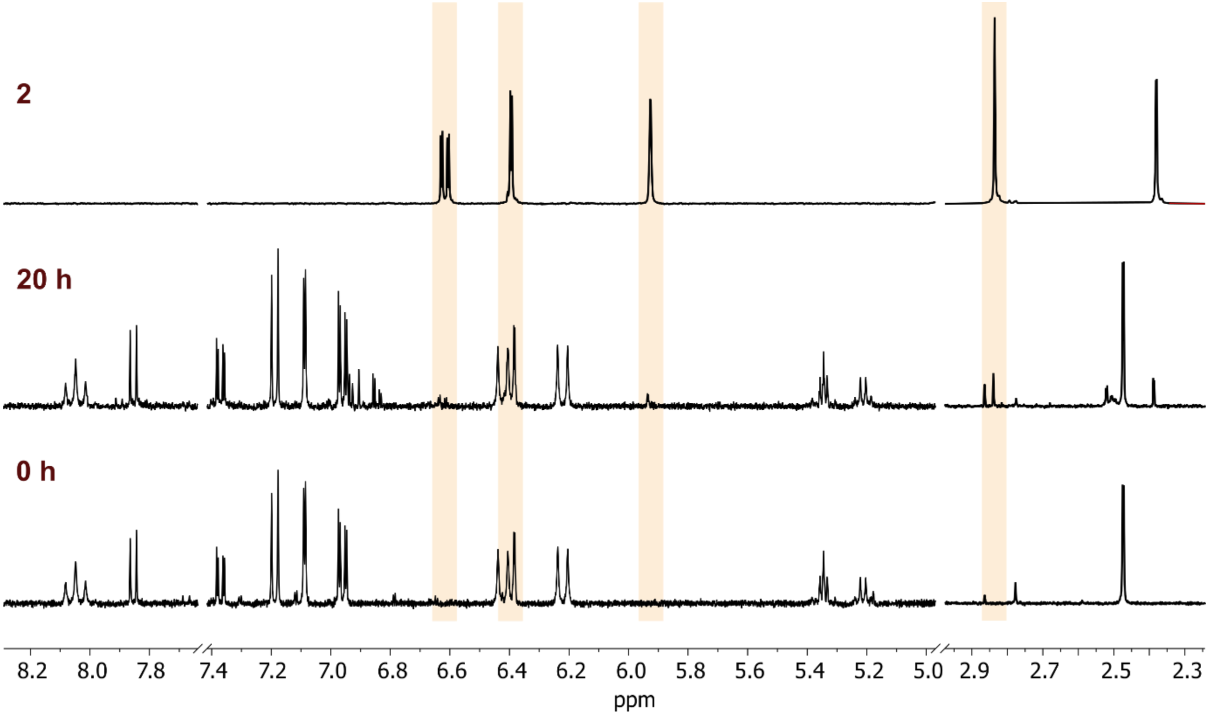

Figure S58. ^{1}H NMR (400 MHz, d_4-MeOH) of **2** and photocage **1b** irradiated in d_4-CD_3OD at 810 nm for indicated time in oxygen-free conditions.

Table S2. Summary of the integrals from Figures **S35–36**, **S40** and **S44** corresponding to the release of coumarin from **1a** and **1b**. Residual peak of d_6-DMSO and d_4-MeOH were used as the internal standards for quantification.

		**+ O_2**			
**Photocage**	**Time [h]**	**Int. d_6-DMSO**	**Int. Photocage**	**Int. Coumarin**	**Yield [%]**
**1a**	0	(δ 2.56 ppm) 124.6H	(δ 6.98 ppm) 2H	-	-
	24		-	(δ 6.58 ppm) 0.52 H	52
**1b**	0	(δ 2.56 ppm) 104.42H	(δ 6.97 ppm) 2H	-	-
	24		-	(δ 6.35 ppm) 0.23 H	12
		**- O_2**			
**Photocage**	**Time [h]**	**Int. d_4-MeOH**	**Int. Photocage**	**Int. Coumarin**	**Yield [%]**
**1a**	0	(δ 0.25 ppm) 8.41H	(δ 6.23 ppm) 2H	-	-
	20		-	(δ 2.39 ppm) 1.42	47
**1b**	0		-	-	-
	20		-	-	<2

* Traces of oxygen were present in the sample despite degassing. Signals corresponding to the ketone product of the photoxidation could be identified, signals corresponding to released cargo contribute to oxygen-dependent uncaging process.

Determination of Yield od H_2S Release with Methylene Blue Assay

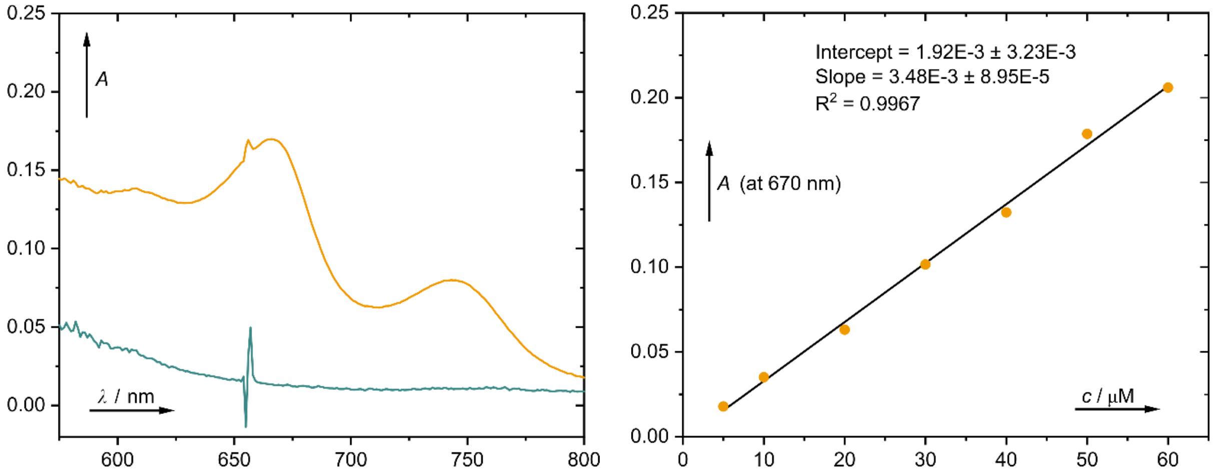

Figure S59. (left) UV-Vis absorption spectra of a MB assay of **1a** (c ~ 150 × 10^{-6} M, 3 mL) in HEPES (pH 7.4, 20 mM, with 1% of MeOH) after irradiation (red) and the same sample kept in the dark (blue). (right) Calibration curve of NaHS in HEPES (pH 7.4, 20 mM with 1% of MeOH).

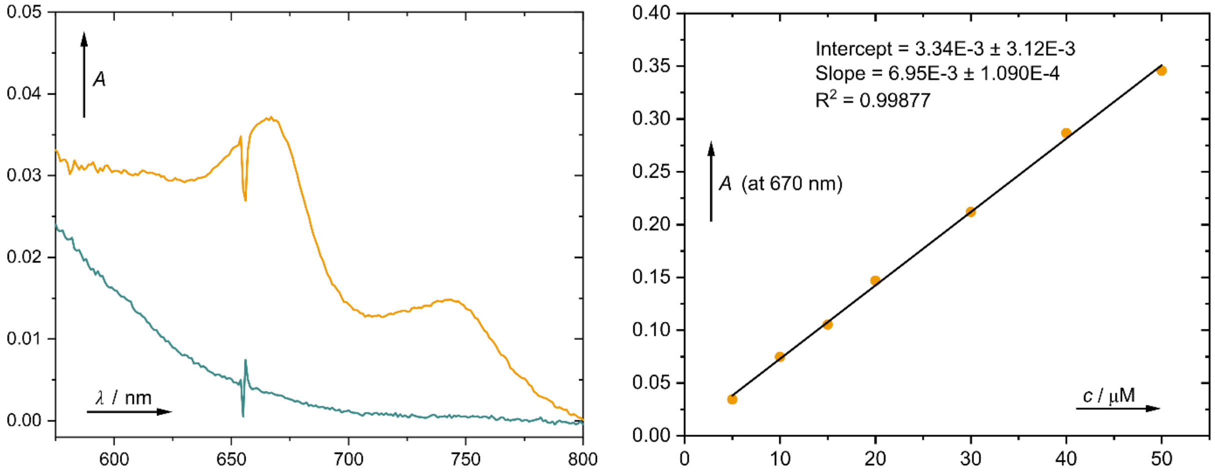

Figure S60. (left) UV-vis absorption spectra of a MB assay of **1b** (c ~ 50 × 10^{-6} M, 3 mL) in HEPES (pH 7.4, 20 mM, with 5% of MeCN) after irradiation (red) and the same sample kept in the dark (blue). (right) Calibration curve of NaHS in HEPES (pH 7.4, 20 mM with 5% of MeCN).

Scheme S4. Suggested competing photooxidative (left) and direct uncaging (right) mechanisms that operate in **1a.**

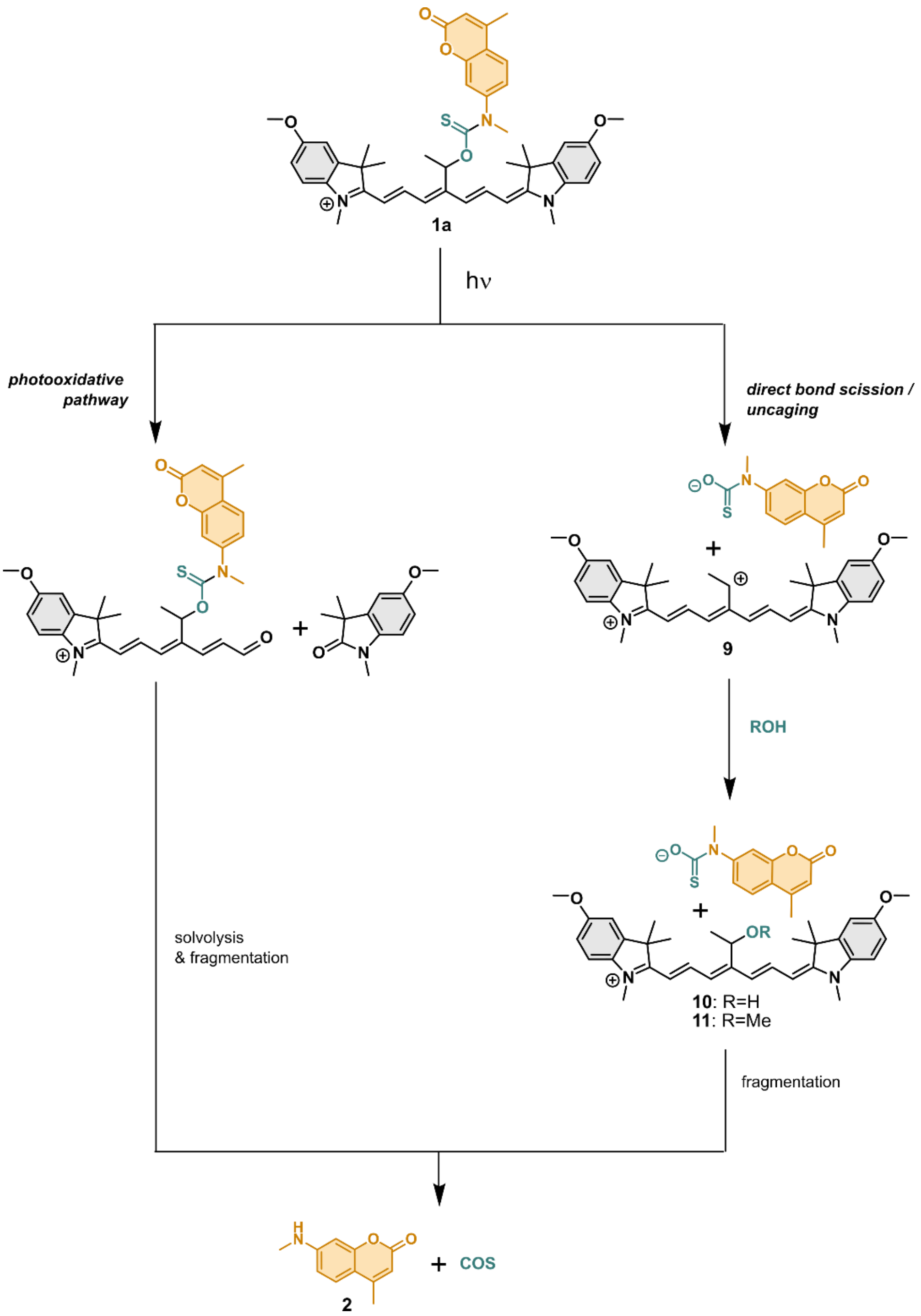

Biological Experiments

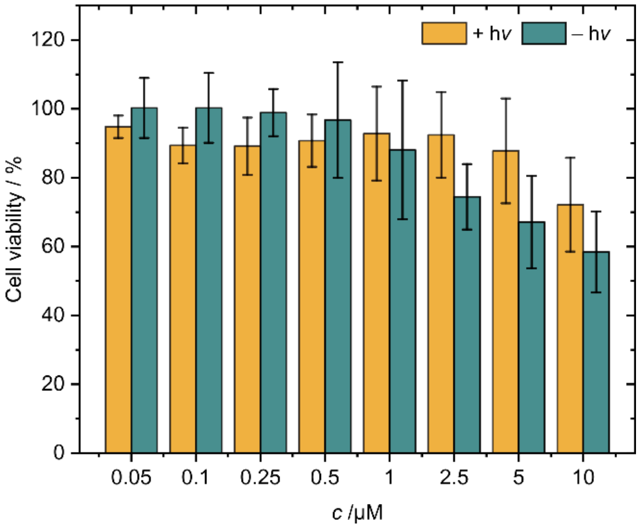

Figure S61. Cell viability after 24h expesure of HeLa cells to **1a** (blue) and photoproducts of **1a** (orange)**.**

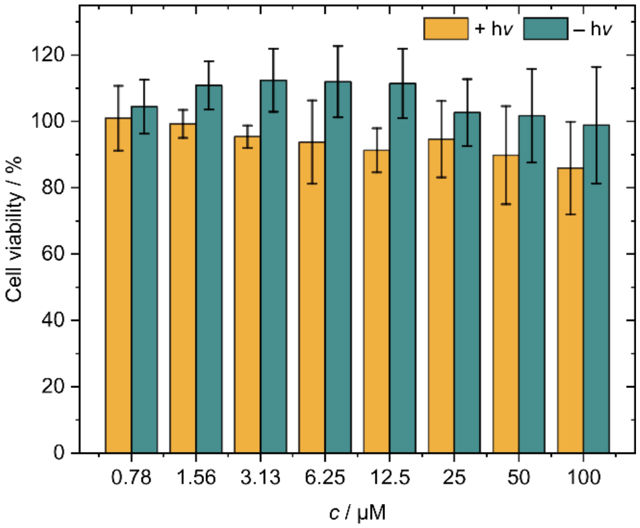

Figure S62. Cell viability after 72h expesure of HeLa cells to **1c** (blue) and photoproducts of **1c** (orange).

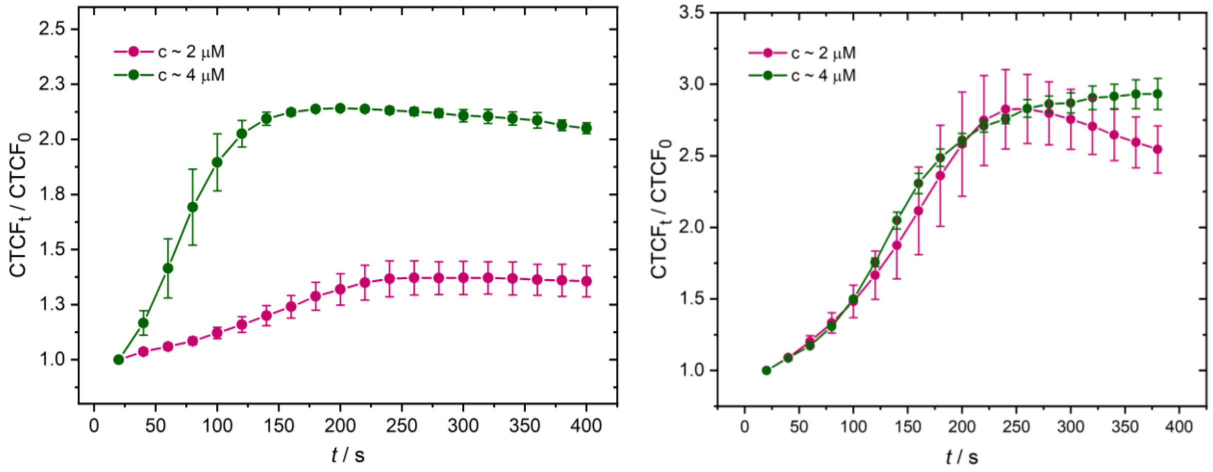

Figure S63. Plot of CTCF enhancement in HeLa cells for the photocage **1a** (c ~ 2 or 4 μM) irradiated at 747 nm; the payload **2** channel (left) and Mito-HS channel (right).

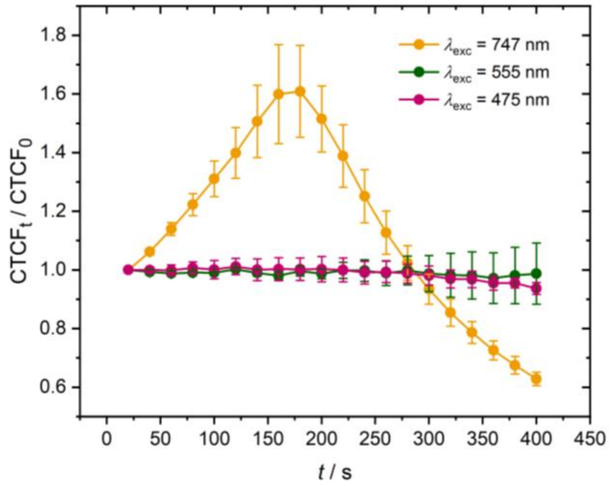

Fgure S64. Plot of CTCF enhancement in HeLa cells incubated with **1a** (c ~ 2 μM) irradiated 475 nm, 555 nm and 747 nm; cyanine (photocage) channel.

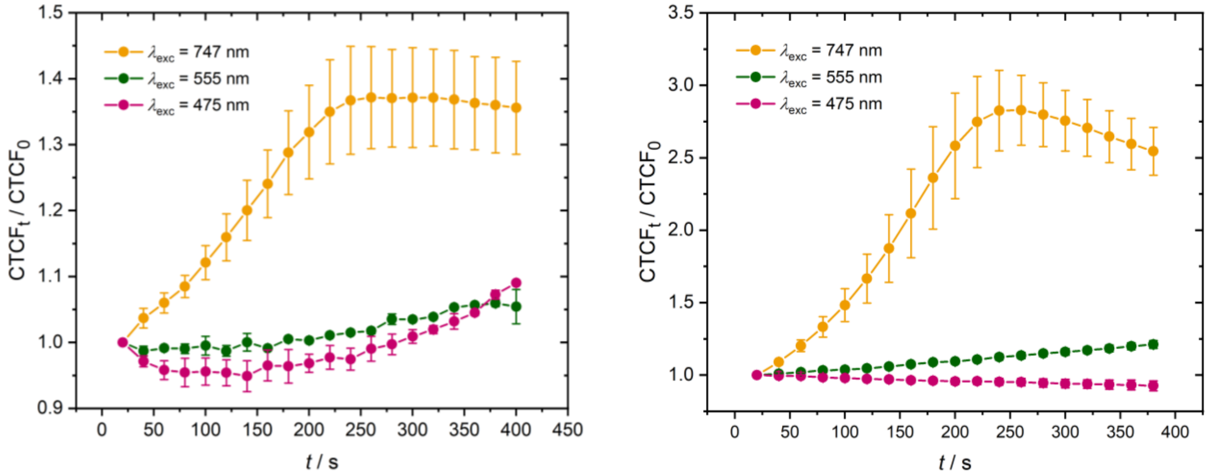

Figure S65. Plot of CTCF enhancement in HeLa cells incubated with **1a** (c ~ 2 μM) irradiated 475 nm, 555 nm and 747 nm; the payload **2** channel (left) and Mito-HS channel (right).

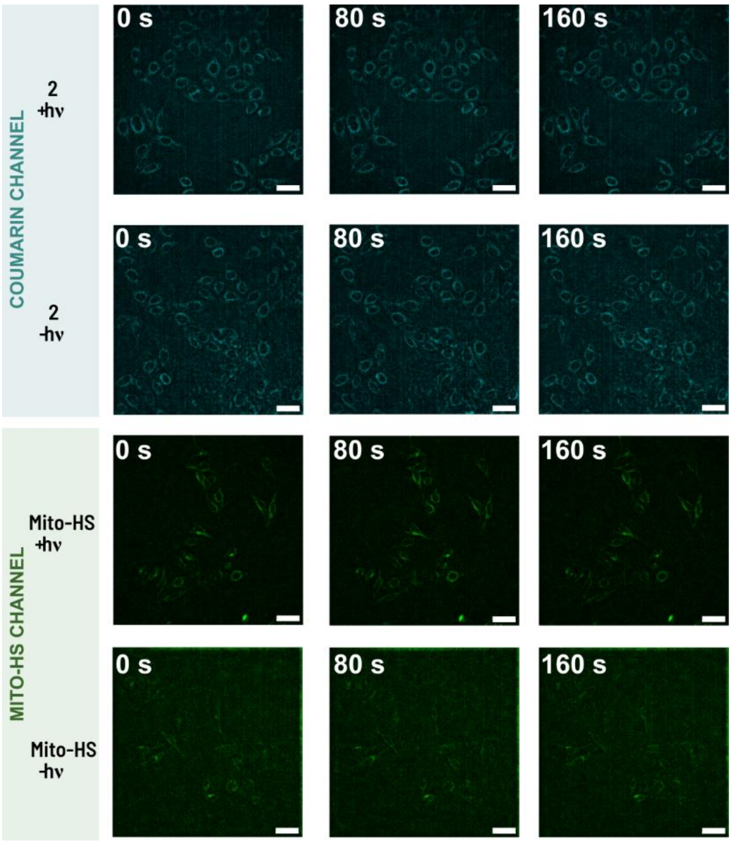

Figure S66. Representative fluorescence microscopy images (40× magnification) of HeLa cells incubated with **2** (top) or Mito-HS (bottom) irradiated with 747 nm light or kept in dark. Images processed by ImageJ after deconvolution and background correction are shown. Scale bar represents 40 μm.

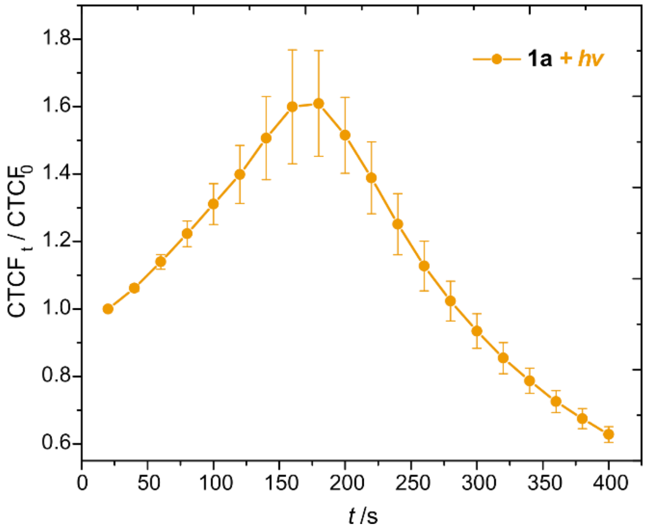

Figure S67. Plot of CTCF enhancement in HeLa cell in the presence of 747 nm irradiation in the photocage channel. The cells were not incubated with the MitoTracker Deep Red in this case.

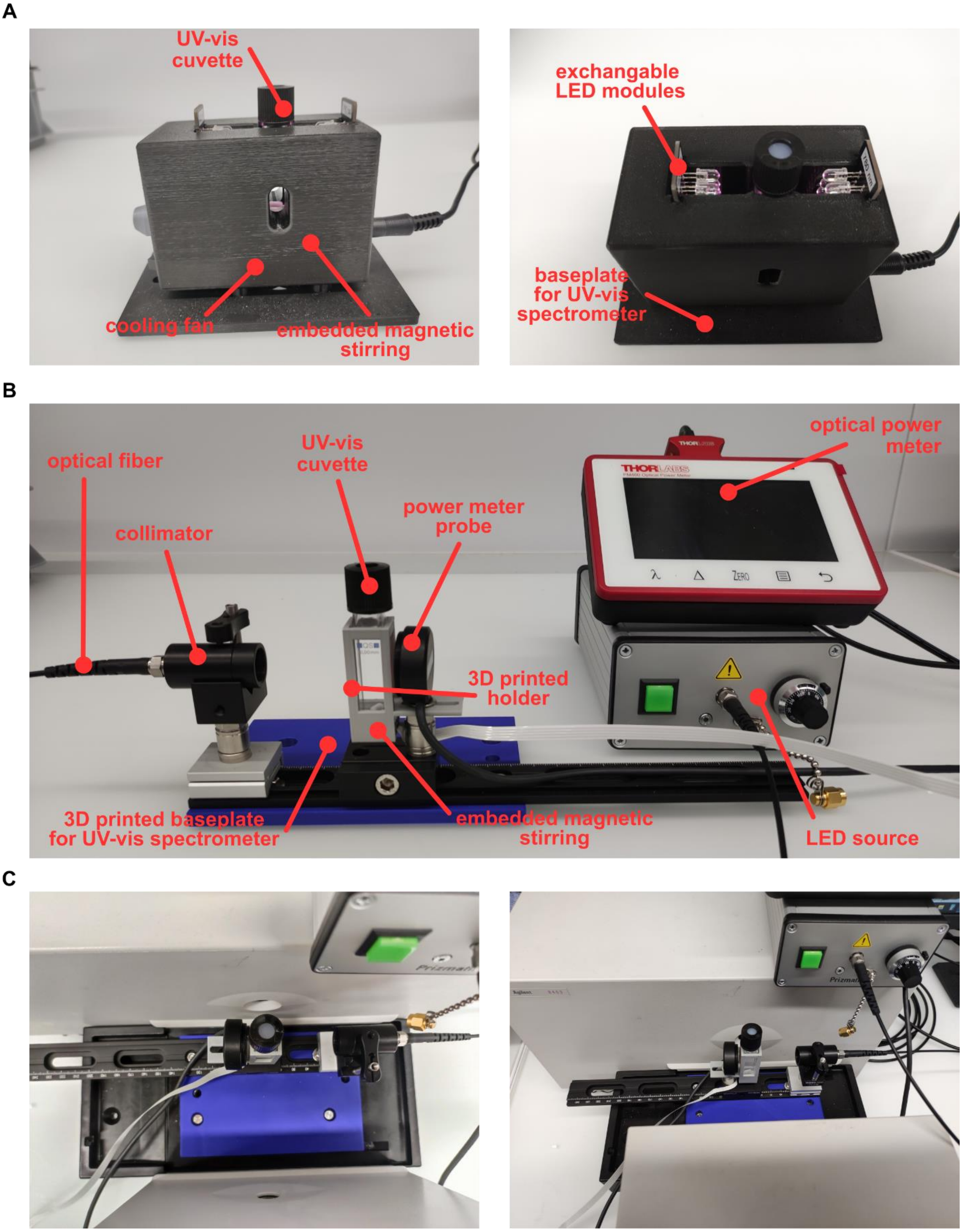

Figure S68. A) Irradiation module with magnetic stirring and exchangeable LED modules. B) In-house built irradiation setup with collimated light beam (780 nm) with magnetic stirring and coupled to optical power meter. C) Setup from (B) mounted inside a UV-vis spectrometer.

References

[1] H. Janeková, M. Russo, U. Ziegler, P. Štacko, *Angew. Chem. Int. Ed.* **2022**, e202204391.

[2] S.M. Hickey, S.O. Nitschke, M.J. Sweetman, C.J. Sumby, D.A. Brooks, S.E. Plush, T.D. Ashton, *J. Org. Chem*. **2020**, 85, 12, 7986–7999.

[3] Commercially available; Compound and spectra (1H and 13C NMR) available at Biosynth ®.

[4] Wu Z., Liang, D., Tang, X. *Anal. Chem.* **2016**, *88*, 9213–9218.

[5] D. Sage, L. Donati, F. Soulez, D. Fortun, G. Schmit, A. Seitz, R. Guiet, C. Vonesch, M. Unser, *Methods* **2017**, *115*, 28–41.

[6] H. Kirshner, F. Aguet, D. Sage, M. Unser, *J. Microscopy.* **2013**, *249*, 13–25.

[7] Y. Zhang, X. Zong, M. Ji, *J. Chem. Res.,* **2019**, 43 (11-12), 542–547.
